# Phylogenetic Molecular Species Delimitations Unravel Potential New Species in the Pest Genus *Spodoptera* Guenée, 1852 (Lepidoptera, Noctuidae)

**DOI:** 10.1371/journal.pone.0122407

**Published:** 2015-04-08

**Authors:** Pascaline Dumas, Jérôme Barbut, Bruno Le Ru, Jean-François Silvain, Anne-Laure Clamens, Emmanuelle d’Alençon, Gael J. Kergoat

**Affiliations:** 1 Institute for Biodiversity and Ecosystem Dynamics, University of Amsterdam, Amsterdam, The Netherlands; 2 Direction des collections—USM 602, Muséum National d’Histoire Naturelle, Paris, France; 3 IRD/CNRS, Laboratoire Evolution Génomes Spéciation, Avenue de la terrasse, Gif-sur-Yvette, France and Université Paris-Sud 11, Orsay, France; 4 Unité de Recherche IRD 072, African Insect Science for Food and Health (icipe), Nairobi, Kenya; 5 INRA—UMR 1062 CBGP (INRA, IRD, CIRAD, Montpellier SupAgro), Montferrier-sur-Lez, France; 6 UM2—UMR 1333 DGIMI, Université Montpellier 2, Montpellier, France; 7 INRA—UMR 1333 DGIMI, Université Montpellier 2, Montpellier, France; Consiglio Nazionale delle Ricerche (CNR), ITALY

## Abstract

Nowadays molecular species delimitation methods promote the identification of species boundaries within complex taxonomic groups by adopting innovative species concepts and theories (e.g. branching patterns, coalescence). As some of them can efficiently deal with large single-locus datasets, they could speed up the process of species discovery compared to more time consuming molecular methods, and benefit from the existence of large public datasets; these methods can also particularly favour scientific research and actions dealing with threatened or economically important taxa. In this study we aim to investigate and clarify the status of economically important moths species belonging to the genus *Spodoptera* (Lepidoptera, Noctuidae), a complex group in which previous phylogenetic analyses and integrative approaches already suggested the possible occurrence of cryptic species and taxonomic ambiguities. In this work, the effectiveness of innovative (and faster) species delimitation approaches to infer putative species boundaries has been successfully tested in *Spodoptera*, by processing the most comprehensive dataset (in terms of number of species and specimens) ever achieved; results are congruent and reliable, irrespective of the set of parameters and phylogenetic models applied. Our analyses confirm the existence of three potential new species clusters (for *S*. *exigua* (Hübner, 1808), *S*. *frugiperda* (J.E. Smith, 1797) and *S*. *mauritia* (Boisduval, 1833)) and support the synonymy of *S*. *marima* (Schaus, 1904) with *S*. *ornithogalli* (Guenée, 1852). They also highlight the ambiguity of the status of *S*. *cosmiodes* (Walker, 1858) and *S*. *descoinsi* Lalanne-Cassou & Silvain, 1994. This case study highlights the interest of molecular species delimitation methods as valuable tools for species discovery and to emphasize taxonomic ambiguities.

## Introduction

Though millions of species remain to be discovered and described [1–4], the discipline of finding, describing and naming species—Taxonomy—currently faces an important deficit in term of funding and manpower [5]. This ‘taxonomy crisis’ is a major issue because the speed at which species are being lost is much faster than the required time to find, describe and potentially protect them [6,7]. The problem is also aggravated by the fact that traditional morphological studies are usually extremely time consuming, especially when dealing with hyper diverse groups such as insects or plants, or when assessing species complexes with superficially indistinguishable morphological attributes [8–13]. Our lack of knowledge is even more problematic when facing species complexes that threaten public health [14,15] or target important crops [16–19]. In this context, some hopes came from the use of molecular data in studies on taxonomy and systematics. Thanks to the increased availability of molecular data over the last decades, DNA-based taxonomy has been proposed as an elegant and effective solution to speed up species identifications and discoveries [20–27]. However, this initiative was quickly criticized on both practical [28–32] and theoretical [33–39] grounds, with numerous studies underlining the fact that molecular data alone (especially molecular barcodes) cannot solve the current taxonomic crisis [40–43]. The general consensus that emerged from these debates was that faster and more accurate species identifications or delimitations can be achieved through the use of multiple complementary sources of information (e.g. behavioural, ecological, molecular, morphological) [17,44,45], hence underlining the need for a more ‘integrative’ [46,47] or ‘iterative’ taxonomy [48].

Because of their ever-increasing accessibility and decreasing cost, it is no wonder that molecular data are now routinely incorporated into taxonomy studies [7,17,45,49–52]. Of particular interest is the fact that multiple analytic procedures have been explicitly developed to delimit species boundaries using molecular data [53–57]. A first class of approaches—referred to as species discovery procedures—assigns samples to putative species clusters without *a priori* information [58]. A second class of approaches—referred to as species validation procedures—requires an *a priori* assignation of samples to putative species clusters, which can be later validated [58]. Both species discovery and species validation approaches can rely on molecular phylogenies, which are increasingly used to help identifying or delimiting species [17,54–56,59–60]. All tree-based species delimitation methods usually rely on the ‘phylogenetic species concept’ (*sensu* De Queiroz [61]), within a coalescent framework [55,62–63], whose central aim is to identify independently evolving lineages without gene flow, in which selection and drift operate, each representing a putative species [64]. Tree-based species delimitation methods can operate with single or multi locus data; the use of multi locus data is mandatory for species validation procedures and optional for species discovery approaches [58]. For species discovery procedures, the use of multi locus data is usually advocated because of its superior power to resolve complex evolutionary histories resulting from recent episodes of radiation, incomplete lineage sorting, gene introgression or species hybridization [58,65,66]. All these processes usually generate discordance between gene trees and species trees [67], which can biases the inference of putative species clusters. Nonetheless, single locus data have been proved useful to provide robust inferences of evolutionary histories [68] and quick assessments of species boundaries and diversity [26,69–72]. More recently they have been also associated with concise morphological descriptions to produce rapid descriptions of new species, an approach referred to as turbo-taxonomy [73–75]. Finally the use of single locus data also benefits from the fact that several genes (e.g. 16S ribosomal RNA) are well represented in large public databases such as GenBank (http://www.ncbi.nlm.nih.gov/genbank/) or the Barcode of Life Database (BOLD; http://www.boldsystems.org/; for the mitochondrial cytochrome c oxidase subunit I gene (COI)).

In this study, we explore the potential interest and efficiency of species discovery approaches to assess species status in moths belonging to the genus *Spodoptera* (Lepidoptera, Noctuidae). This genus encompasses 30 species [76], and includes several species whose status has been questioned in the past based on biological [77–79], morphological [80,81] and molecular evidence [82–85]. Clarifying *Spodoptera* species boundaries is of prime importance because half of known *Spodoptera* species are pests of economic importance that target major crops such as beans, cotton, maize, millet, rice, sorghum, soybean or tomatoes [76,86,87]. A particular focus will be made on the fall armyworm (FAW) *Spodoptera frugiperda*, whose identity is particularly debated [88–91]. The fall armyworm is a widespread and well-known agricultural pest in the Western hemisphere [76,87]. It is an extremely polyphagous species, which is known to develop on about 100 different plants species from 27 plant families [76]. Several features of the biology and the ecology of fall armyworm indicate that this species is composed of two morphologically undistinguishable ecological races (‘corn form’ and ‘rice form’ *sensu* Juarez *et al*., [91]), with distinct host-preferences [92–94]. The use of diagnostic molecular markers [95–99] have indicated that both strains are generally found in sympatry [92,100–101], hence leading some authors to argue that they could constitute a potential example of ongoing or sympatric speciation [78,88]. Both strains also exhibit a high level of genetic differentiation [85]. In addition to the case of *S*. *frugiperda*, we will also pay attention to five other *Spodoptera* species, whose species status is still disputed [81]. It is the case of *S*. *marima* and *S*. *ornithogalli*, which are morphologically related and possibly correspond to geographic races, as they have allopatric distributions and only differ by the absence of sexual dimorphism in *S*. *marima* males [76,81]. These two species were not found reciprocally monophyletic in a recent multilocus phylogenetic study [85], so we expect additional clarifications with the molecular species delimitation procedures. Another species pair (*S*. *cosmiodes* and *S*. *descoinsi*) was also recovered paraphyletic with a strong support in the study of Kergoat *et al*., [85], but similarly to the case of *S*. *frugiperda*, additional evidences (differences in pheromone blends, allochronism of females) led to think that these two entities could possibly constitute valid species [76,77]. Finally, the last case that remains to be investigated is the status of Western populations (from East Africa, Mauritius, the Malagasy Republic, and the Reunion Island) and Eastern populations (from the Indomalayan and Australasian regions) of *S*. *mauritia*, which may constitute valid species of their own [80]. Here we will explore *Spodoptera* species boundaries by using two different tree-based approaches that have drawn much attention in recent years [102–110]: the general mixed Yule-coalescent (GMYC) model [54,108] and the Poisson-Tree-Processes (PTP) [60]. The GMYC model uses the distinct branching patterns between a Yule model (modelling interspecific speciation events) and a coalescent model (modelling expected coalescent times of genes at an intraspecific level) to distinguish between species and population processes. This model is fitted to an ultrametric tree (a tree where all the path-lengths from the root to the tips are equal) and optimized under maximum likelihood to find the threshold time (*T*), which corresponds to the transition between the two distinct branching patterns. It is possible to visualise this transition with a lineage-through-time plot, in which a sharp increase in branching rates is expected at *T*. Potential species clusters are then determined by identifying the clades (or single lineages) that originate after this putative threshold. The PTP is a recently developed method, which has the advantage of not requiring an ultrametric tree (inferring an ultrametric tree is usually a time-consuming and potentially error-prone process [103,110]); instead the PTP model uses branch lengths to estimate the mean expected number of substitutions per site between two branching events. The model assumes that each substitution has a small probability of generating a speciation event; hence the number of substitutions between species is expected to be significantly higher than those within species [60]. The model then implements two independent classes of Poisson processes (one describing speciation and the other describing within species branching events) and searches for transition points between interspecific and intraspecific branching patterns. Potential species clusters are then determined by identifying the clades (or single lineages) that originate after these transition points. Because both methods efficiently deal with large single-locus datasets [110], they can readily benefit from the mass of sequence data generated through the Barcode of Life (BoL) initiative or deposited on public database such as GenBank. Here we combine *Spodoptera* sequences generated from previous studies that are available on GenBank or BOLD with newly generated sequences for the COI gene. The resulting dataset will be used to: (*i*) infer putative species boundaries in the genus *Spodoptera* by applying species discovery methods particularly suitable to deal with large single-locus datasets; (*ii*) to test the reliability and the usefulness of these species discovery methods by comparing our results with other sources of information.

## Material and Methods

### Sampling design

The sampling for this study covers 27 of the 30 known *Spodoptera* species [76]. We were unable to acquire sequence data for three species: *S*. *compta* (Walker, 1869) (from Peru), *S*. *malagasy* Viette, 1967 (from Madagascar) and *S*. *roseae* (Schaus, 1923) (from the Galapagos Islands). Both *S*. *compta* and *S*. *roseae* are extremely rare in collections (especially *S*. *compta* which is only known from three specimens collected in the 19^th^ century). As for *S*. *malagasy*, previous sequencing attempts on several old museum specimens have been unsuccessful [85]. For this study we generated 86 new sequences for specimens obtained from the Muséum National d’Histoire Naturelle in Paris (MNHN). In addition we used 118 sequences from the study of Kergoat *et al*., [85], 463 additional sequences from GenBank and five sequences from BOLD. The resulting dataset encompasses 672 *Spodoptera* specimens, with a mean number of 24 individuals per species (from two individuals for *S*. *apertura* (Walker, 1865), *S*. *pecten* Guenée, 1852, *S*. *pulchella* (Herrich-Schäffer, 1868) and *S*. *triturata* (Walker, 1857) to 101 in *S*. *frugiperda*; S1 Table). All specimens, their geographic origin and the corresponding sequence accession number are listed in a supplementary table (S1 Table). For outgroup choice we used the results of the study of Mitchell *et al*., [111] to select the following seven noctuid taxa: *Agrotis ipsilon* (Hufnagel, 1766) (Noctuinae), *Helicoverpa armigera* (Hübner, 1805) (Heliothinae), *Heliothis virescens* (Fabricius, 1777) (Heliothinae), *Mythimna unipuncta* (Haworth, 1809) (Hadeninae), *Noctua atlantica* (Warren, 1905) (Noctuinae), *Psychomorpha epimenis* (Drury, 1782) (Agaristinae), *Sesamia inferens* (Walker, 1856) (Xyleninae). Based on Mitchell *et al*., [111] all trees were rooted with *Psychomorpha epimenis*, which belongs to a clade that is sister to the clade including both *Spodoptera* spp. and the subfamilies Hadeninae, Heliothinae, Noctuinae and Xyleninae.

### DNA extraction, Polymerase chain reactions and Sequencing

Total genomic DNA of new samples was extracted by grinding up hind legs using DNAeasy Blood and Tissue Kit (Qiagen). Partial fragments of the COI gene were amplified following Kergoat *et al*., [86]. The polymerase chain reaction (PCR) mix (25 μL) consisted of 1xPCR buffer, 1.5mM MgCl2, 0.1mM of each desoxynucleotide triphosphates, 1μM of each primer, 1 unit of Taq DNA polymerase (Qiagen) and 2 μL (diluted to tenth) of extracted DNA and nuclease-free water to 25 μL. PCR cycling conditions were the following: initial denaturation step at 95°C for 3 min followed by 35 cycles including a denaturation step at 92°C for 1 min, an annealing step at 48°C for 1 min, and an elongation step at 72°C for 1 min and a final elongation step at 72°C for 10 min. The resulting PCR products were processed by Eurofins MWG Synthesis GmbH (Ebersberg, Germany). Both strands were sequenced for all specimens to minimize PCR artifacts and ambiguities.

Sequences of complementary strands were edited and reconciled using Geneious v5.1 software (available at: www.geneious.com/). The alignment of all sequences was carried out using MAFFT 7 [112] with default option settings. No gap was present in the corresponding alignment. We then used Mesquite v3.0 [113] to check the coding frame for possible errors or stop codons. Additional sequences generated for the study were deposited to GenBank under numbers KF854152 to KF854234. The final dataset encompasses 679 individuals and 658 aligned characters.

### Gene tree inference

Phylogenetic analyses were conducted using Maximum likelihood (ML) and Bayesian inference (BI). For both methods we carried out partitioned analyses to improve phylogenetic accuracy [114]. Partitions and substitution models were determined using PartitionFinder v1.1.1 [115]. The corrected Akaike information criterion (AICc; [116]) was used as a metric for ML analyses whereas the Bayesian information criterion (BIC) was used for BI analyses.

Maximum Likelihood analyses were performed using RAxML v8 [117]. Based on the AICc results (S2 Table) we used only one partition with a General time reversible (GTR) model. Instead of using a standard GTR+G+I model we used the GTR CAT approximation model that accommodate searches incorporating rate heterogeneity with faster inference times and better maximum likelihood values [118]. The best ML tree was obtained using a heuristic search implementing 100 random-addition replicates. Clade support was then assessed using a non-parametric bootstrap procedure (1,000 replicates were used). Nodes supported by bootstrap values (BV) ≥ 70% were considered as strongly supported following Hillis and Bull [119].

Bayesian inference analyses were carried out using MrBayes 3.2.2 [120] and BEAST 1.8 [121]. Based on the BIC results (S2 Table) we used three partitions. For MrBayes analyses, instead of using a specific model for each partition we used the *mixed* model option. The latter allows to sample across the substitution model space in the Bayesian Markov Chains Monte Carlo (MCMC) analysis itself, removing the need for *a priori* model testing [122]. We conducted two independent runs with four MCMC (one cold and three incrementally heated) that ran for 50 million generations, with trees sampled every 1,000 generations. A conservative burn-in of 25% was then applied after checking for stability on the log-likelihood curves and the split-frequencies of the runs under MrBayes (a threshold of 0.05 was used). For the BEAST analyses, we implemented the models selected by PartitionFinder (S2 Table). Following Tang *et al*., [110] we also relied on distinct sets of models to infer trees with branch lengths proportional to time. To limit the size of parameter space the majority-rule consensus topology from MrBayes analyses was used as a guide tree. We alternatively used a strict clock model or an uncorrelated lognormal (UCLN) relaxed clock as clock priors, and a bird-death (BD) model as tree prior. The corresponding analyses were carried out using two independent runs of 50 million generations, with trees sampled every 1,000 generations. A conservative burn-in of 25% was then applied after checking for the effective sampling sizes (ESS) of parameters (a threshold of 200 was used). Support of nodes for both MrBayes and BEAST analyses was provided by clade posterior probabilities (PP) as directly estimated from the majority-rule consensus topology. Nodes supported by PP ≥ 0.95 were considered as strongly supported following Erixon *et al*., [123].

### Molecular species delimitations

We followed the recommendations of Tang *et al*., [110], who advocated the use of model-based gene trees (generated with programs such as MrBayes or RAxML) for PTP analyses and the use of ultrametric trees generated with BEAST for the GMYC analyses. Poisson-Tree-Processes molecular species delimitations were conducted on the Web server of the Exelixis Lab (http://species.h-its.org/ptp/), with default settings. The corresponding analyses were carried out using trees either the best tree from the ML analyses or the majority-rule consensus topology resulting from the BI analyses. To avoid biases that may arise if some of the outgroup taxa are too distantly related from ingroup taxa, outgroups were pruned before conducting the PTP analyses for both ML and BI trees. General mixed Yule-coalescent analyses were performed under R (R Development Core Team 2010) using the latest implementation of the GMYC procedure [108], which relies on the following packages: *splits* [124], *APE* [125] and *apTreeshape* [126]. All corresponding analyses were carried out using a single threshold with a confidence interval falling within 2 log-likelihood units of the ML solution [54].

## Results

### Gene tree inference

**Fig 1:**
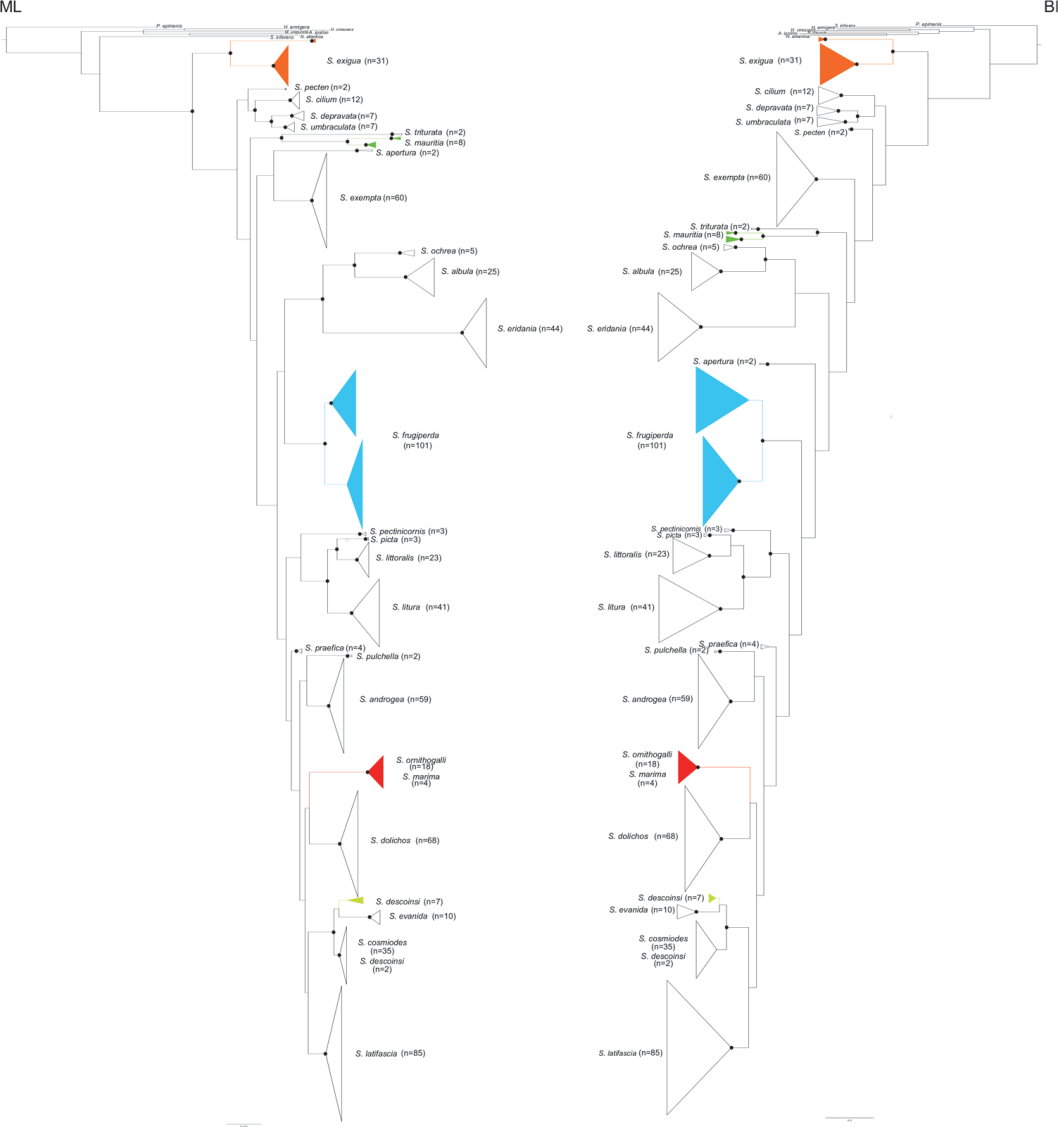
COI-based phylogenetic relationships of *Spodoptera* species. The tree on the left summarizes the results of ML analyses while the tree on the right summarizes the results of BI analyses. Black dots indicate nodes supported by bootstrap values ≥ 70% (ML tree) or supported by posterior probability ≥ 0.95 (BI tree).

The phylogenetic analyses in ML and BI show similar results (Fig 1; see also S1 and S2 Figs for details), which only differ by the position of some outgroups and the placement of two *Spodoptera* species (*S*. *apertura* and *S*. *pecten*). In both ML and BI analyses, the genus *Spodoptera* is recovered monophyletic. Support for *Spodoptera* monophyly was higher for the ML tree (BV of 95%) in comparison to the BI tree (PP of 0.94). The phylogenies are overall not resolved, except for the terminal nodes. Support values are equally high for the nodes leading to morphospecies (BV ≥ 70% for 23 out of the 27 corresponding nodes under ML; PP ≥ 0.95 for 21 out of the 27 corresponding nodes under BI).

Four of the 27 *Spodoptera* morphospecies (*S*. *cosmiodes*, *S*. *descoinsi*, *S*. *marima* and *S*. *ornithogalli*) are consistently recovered paraphyletic in all analyses. Out of the nine sampled *S*. *descoinsi* individuals (all from French Guiana), seven of them constitute a well-supported clade, sister to *S*. *evanida* Schaus, 1914 (specimens of *S*. *evanida* were collected in French Guiana and Peru). The two remaining specimens identified as *S*. *descoinsi* (voucher codes B19 and LSU30) are grouped in a moderately to highly supported clade (BV of 88%, PP of 0.87) that includes all sampled *S*. *cosmiodes* individuals (from Brazil and French Guiana). Regarding *S*. *marima* and *S*. *ornithogalli*, the four sampled specimens of *S*. *marima* (from French Guiana and Venezuela) are mixed with the 18 sampled specimens of *S*. *ornithogalli* (from Canada, Dominican Republic, Guatemala, French Guiana and USA) in a well-supported clade (BV of 100%, PP of 1.0).

Two distinct and well-supported clades were inferred for *S*. *frugiperda* (BV of 100 and PP of 1.0). A first clade (58 specimens) includes all the specimens that have been assigned to the ‘rice form’ in the studies of Nagoshi *et al*., [127] and Kergoat *et al*., [85] whereas the other one (43 specimens) includes all the specimens previously assigned to the ‘corn form’. Two distinct and well-supported clades were also recovered for *S*. *mauritia*. A first clade (BV of 96%, PP of 1.0) groups three specimens collected in the Réunion Island that belong to the nominate form (*S*. *m*. *mauritia*) distributed in Madagascar and neighboring islands (Comoro, Mauritius and Réunion). A second clade (BV of 98%, PP of 1.0) groups five specimens collected in Australia (KF389305), Japan (AB733407, AB733408 and AB733409) and Papua New Guinea (voucher code B152) that belong to *S*. *m*. *acronyctoides*, which is distributed in the Oriental, Indo-Australian and Pacific regions.

### Molecular species delimitations

The results of phylogenetic molecular species delimitations were compared to morphological assignations that follow the checklist of species provided by Pogue [76].

**Fig 2:**
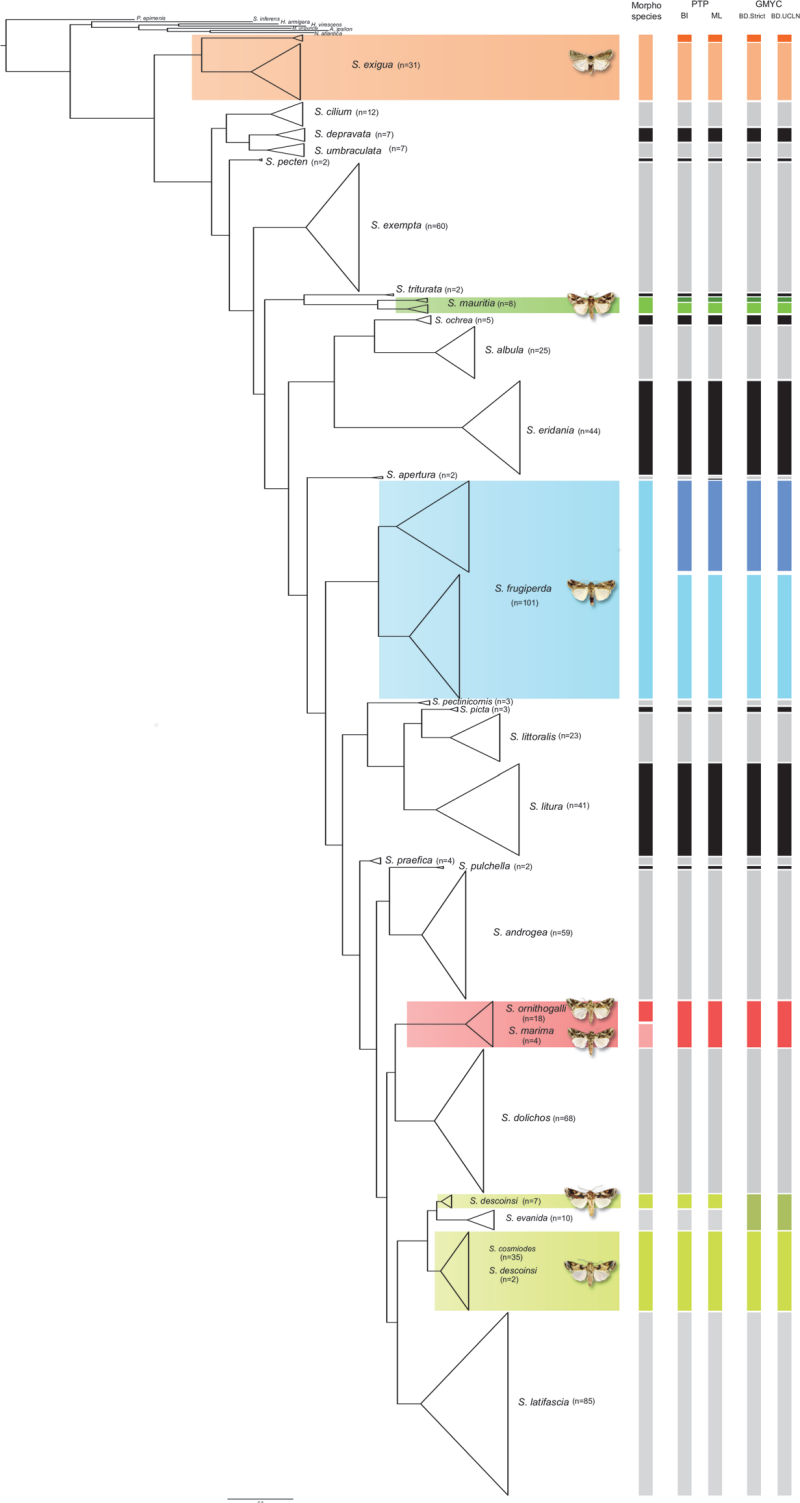
Putative species clusters corresponding to: (i) morphological delineations (column 1); (ii) PTP analyses using either the majority-rule consensus topology resulting from the BI analyses (column 2) or the best tree from the ML analyses (column 3); (iii) GMYC analyses based either on a strict clock and a BD model (column 4) or on a UCLN clock and a BD model (column 5). The reference tree used here corresponds to the tree resulting from the BI analyses carried out with MrBayes.

Regarding the PTP analyses, at least 29 putative species clusters were recovered for the 67 specimens sampled from 27 *Spodoptera* morphospecies (Fig 2, S3 and S4 Figs and S3 Table). 30 putative species clusters were inferred for the analyses based on the best-fit ML tree (S3 Fig) while 29 putative species clusters were inferred for the analyses based on the BI majority-rule consensus topology (S4 Fig). Two distinct putative species clusters were consistently found for the following three species: *S*. *exigua*, *S*. *frugiperda* and *S*. *mauritia*. In addition the two individuals of *S*. *apertura* were also recovered as distinct putative species in the analyses based on the ML tree. Regarding *S*. *exigua*, a putative species cluster groups the four sampled Australian specimens (KF522648, KF522649, KF522650 and KF522651) whereas a second one groups the remaining 27 *S*. *exigua* specimens. For *S*. *frugiperda* the two putative species clusters consist of 43 and 58 specimens, respectively. The smaller FAW cluster groups all individuals that have been assigned to the ‘corn form’ by previous studies [85,127] whereas the bigger FAW cluster groups all individuals previously assigned to the ‘rice form’ [85,127]. For *S*. *mauritia* the two putative species clusters distinguish the specimens belonging to *S*. *m*. *mauritia* (three individuals collected in the Réunion Island) with the specimens belong to *S*. *m*. *acronyctoides* (five individuals collected in Australian, Japan an Papua New Guinea). All individuals belonging to *S*. *marima* and *S*. *ornithogalli* are lumped in one putative species cluster encompassing 22 specimens (18 for *S*. *ornithogalli* and four *S*. *marima*). Concerning *S*. *cosmiodes* and *S*. *descoinsi*, the PTP species delimitation recovered two putative species clusters (see S4 Fig). A first potential species cluster is made of seven *S*. *descoinsi* individuals, which are sister to the clade corresponding to *S*. *evanida*. A second cluster encompasses all *S*. *cosmiodes* individuals plus two *S*. *descoinsi* specimens.

For the GMYC procedure, the same putative species clusters were inferred for the analyses based either on a strict clock and BD model or on a UCLN clock and BD model (see Fig 2, S5 and S6 Figs and S3 Table). 28 putative species clusters were recovered, with a confidence interval of 27–31 for the tree based on a strict clock and BD model and a confidence interval of 27–30 for the tree based on a UCLN clock and BD model. In comparison with the PTP analyses, the exact same putative species clusters were inferred with the exception of a larger cluster which groups a clade of seven *S*. *descoinsi* individuals with the clade corresponding to *S*. *evanida* (see Fig 2 and S3 Table). When examining the taxa for which changes of status are suggested we found out that most changes are statistically supported (see S5 and S6 Figs). It is the case of the grouping of *S*. *marima* with S. *ornithogalli* and of the splits of *S*. *exigua*, *S*. *frugiperda* and *S*. *mauritia*. The only exception is the case of the grouping of the seven *S*. *descoinsi* individuals with the clade corresponding to *S*. *evanida*, which is not statistically supported.

## Discussion

### A cautionary tale of consistency

The results of our phylogenetic species delimitations analyses are remarkably congruent, as they recover the same species clusters (except for the case of *S*. *apertura* in one of the PTP analyses and the case of *S*. *descoinsi* and *S*. *evanida* in one of the GMYC analyses) whatever the set of parameters or methods used. The number of species clusters inferred using PTP procedures also falls well within the confidence interval of the GMYC analyses (27–31 and 27–30 for the strict clock/BD model and UCLN clock/BD model associations, respectively). Although our study may give the impression that the results of species discovery procedures are expected to be always consistent whatever the models and sets of parameters used, we would like to insist on the fact that reliable and consistent results cannot be always achieved. Several studies have clearly highlighted the fact that most molecular species delimitation approaches (including tree-based species delimitation procedures) are highly susceptible to multiple biases or to the way the analyses are parameterized (see the review of Carstens *et al*., [58]). For instance, model performances are known to be extremely sensitive to biases such as insufficient or unbalanced sampling [31,34], heterogeneous rates of gene evolution [128], incomplete lineage sorting, or hybridization after introgression events [129]. Though rarely look upon, tree building procedures also matter, as different methods and sets of parameters can potentially yield very distinct topologies, which will likely generate contrasting putative species clusters. For example, a potential source of discrepancy is the estimation of branch lengths, as branch length estimates are known to be sensitive to the choice of substitution models [130–132] or partitioning strategies [133,134]. It is especially the case in phylogenetic analyses that use multiple partitions and/or complex models, for which over-parameterization can potentially bias branch length estimates [134,135]. In addition, it has been recently demonstrated [136] that branch length priors (within a Bayesian framework) can significantly impact branch length estimation, with shorter priors generating shorter branch lengths and longer priors generating longer branch lengths. Another potential source of discrepancy is the choice and parameterization of the ultrametrization procedures for methods (such as GMYC) that require ultrametric trees as input trees. In the study of Astrin *et al*., [103] on cryptorhynchine weevils (Coleoptera) the authors used 21 distinct sets of parameters and methods. They showed that the number of putative species clusters inferred using GMYC could vary drastically with numbers varying from 5 to 564 depending on the choice of methods and parameters. Similarly Tang *et al*., [110] highlighted the fact that the branch smoothing procedures are a potential source of biases for the approaches that require ultrametric trees.

In summary all these studies underline that molecular species delimitation approaches should not be used blindly. They nonetheless constitute powerful tools that can be effectively used in a concerted way to assess species boundaries (e.g. [137]). In our study, a high level of consistency was likely achieved because of the size of our dataset and because we carefully followed some of the recommendations of Tang *et al*., [110]. Because the focus of our study was to reassess *Spodoptera* species boundaries, and not to explore the impact of tree building procedures on molecular species delimitations, we feel that our analytical pipeline is appropriate and allows us to discuss with some confidence the ambiguous species status of several taxa.

### *Spodoptera* species boundaries, a reappraisal

A clear pattern is evidenced for the species that is the main focus of our study, *Spodoptera frugiperda*. Individuals are separated into two clusters that likely correspond to the two known host-forms (rice and corn) of *S*. *frugiperda*. Both PTP and GMYC analyses indicate that the level of genetic differentiation exhibited by the two host-forms is compatible with a potential sister-species status, with a significant statistical support under the GMYC procedure. This result is in agreement with the results of several studies that have highlighted the existence of prezygotic and postzygotic isolation between the two host-forms (Table 1). Different prezygotic isolation mechanisms have been found, including differences in the composition of females' pheromones [78,138], allochronism in mating activity between the two strains [79,139] and oviposition preferences [94,140]. Regarding postzygotic isolation, the success of mating between the two strains remains highly controversial. On the one hand Pashley and Martin [141] and Dumas *et al*., [142] have observed unidirectional mating between corn females and rice males while the reciprocal cross gave viable offsprings. Furthermore, in backcrosses the hybrid females mated with low success with their brothers but not at all with males of either parental strain [140,141,143]. On the other hand, another study has shown that the two strains crossed successfully in both directions [144]. The latter is supported by the fact that a high proportion of hybrid has been evidenced is several surveys of natural populations [90,145]. Finally, a postzygotic isolation for several life-history traits in both host-forms has been also evidenced by Velasquez-Velez *et al*., [146] who identified a reduction of the number of hybrid females and a reduction in hybrid fertility in *S*. *frugiperda*. Taken altogether all these studies suggest that both host-forms are situated somewhere in between a ‘continuum’ of speciation [147]. In this context, the results of our study tend to favour the hypothesis that the two host-forms are well beyond the early steps of speciation as they present a level of genetic differentiation which is comparable with those observed between other distinct *Spodoptera* species.

**Table 1:** List of evidences arguing in favor of a high level of differentiation between the two FAW strains.

	Suitability	References
**Pre-zygotic barrier**		
Habitat isolation	+ / -	Whitford *et al*., [140] Meagher *et al*., [94] Juárez *et al*., [91]
Temporal isolation	++	Schöfl *et al*., [79,139]
Behavioral isolation	++	Groot *et al*., [78] Lima and McNeil [138]
Unidirectional mating	+/-	Pashley and Martin [141] Dumas *et al*., [142]
**Post-zygotic barrier**		
Reduction of hybrid fertility and reduction of hybrid female numbers	+	Velásquez-Vélez *et al*., [146]
**Genetic differentiation**		
Phylogenetic analyses	++	Kergoat *et al*., [85]

As indicated by the combination of pros and cons some results are more or less contradictory.

In the case of *Spodoptera mauritia*, the results of all phylogenetic species delimitation procedures suggested that Western (*S*. *m*. *mauritia* sensu Fletcher [148]) and Eastern (*S*. *m*. *acronyctoides* sensu Fletcher [148]) populations should be considered as distinct species. Though we cannot exclude the hypothesis of a possible analytical bias resulting from our limited sampling (only three individuals from La Reunion for *S*. *m*. *mauritia* and five individuals from Australia, Japan and Papua New Guinea), the suggested split is nonetheless statistically supported. From a morphological point of view, several authors (e.g. Fletcher [148]) noted differences in forewing coloration, where the conspicuous white areas of the forewing of Western populations are reduced in extent in Eastern populations. However the examination of long series of specimens from the two distribution areas by Brown and Dewhurst [80] did not reveal marked differences between putative subspecies. Additional studies are thus definitely required here to precise the status of both entities, such as comparisons of genitalia or pheromone compounds in female.

Regarding *Spodoptera marima* and *Spodoptera ornithogalli*, for which only few morphological (hindwing coloration and the lack of sexual dimorphism in *S*. *marima* [81]; differences exist, both analyses merge them into a single species cluster (with a significant statistical support under GMYC), hence providing more support to put these taxa into synonymy [85]. Because the two species occur in different geographic areas—*S*. *marima* is only found in the Western and Eastern Neotropics whereas *S*. *ornithogalli* is found in the Northern Neotropics and in the South, Western and Eastern Nearctic [76]—they could be considered as distinct subspecies of *S*. *ornithogalli* (due to priority, as *S*. *ornithogalli* was described before *S*. *marima*).

Interestingly our analyses suggest that *Spodoptera exigua*, a taxon with no ambiguous status, seems well differentiated, and may correspond to two distinct species. 27 of the 31 sampled specimens (including specimens from Canada, Egypt, Germany, Kenya, India, Japan, Thailand and United States of America) constitute a first putative species cluster whereas the four specimens from Australia constitute another one (note that no Australian samples were included in the study of Kergoat *et al*., [85]). This result is also statistically supported under GMYC and does not seem artefactual given the marked level of genetic differentiation between the two clades. It is also supported by additional researches on the Barcoding of life database (BOLD; available at http://www.boldsystems.org/); 81 *S*. *exigua* specimens from Australia are grouped into a distinct barcode cluster (BOLD:AAA6645) distinct from another barcode cluster (BOLD:AAA6644) grouping all remaining *S*. *exigua* individuals (n = 281). Obviously this result requires more investigation in order to determine whether these specimens correspond to a new species with marked morphological differences or to a case of cryptic species complex.

For *Spodoptera apertura*, only one of the four distinct sets of analyses (PTP using the ML tree; S3 Fig) recovers two distinct species clusters for the two sampled specimens of the species. We suspect that this result is artefactual; it possibly results from an artefact in the estimation of branch lengths under ML, where the two terminal branches leading to both individuals are really unbalanced (S3 Fig) whereas there are balanced under BI (S4 Fig). Insufficient sampling (only two specimens from Australia were sequenced for this species) could also have caused this likely error.

The situation is more complex in the case of *Spodoptera cosmiodes* and *Spodoptera descoinsi*, two taxa for which marked morphological and biological differences exist [76,77]. Both phylogenetic species delimitation procedures suggested the existence of two distinct species clusters. Analyses following the GMYC procedure also included representatives of *S*. *evanida* in one of the two clusters (the one with seven specimens *S*. *descoinsi*) but this grouping seems artefactual as the two species are morphologically not related [76]; in addition a closer examination of the GMYC output indicates that this grouping is not statistically supported. If we consider the remaining specimens, we can distinguish two species clusters, one that groups seven *S*. *descoinsi* and another that groups all (n = 35) *S*. *cosmiodes* individuals plus two representatives of *S*. *descoinsi*. To precise the status of these two individuals, additional studies are likely required; we advocate for the use of species validation procedures (with packages such as BPP [55] or SpedeSTEM [56]), using more specimens and a multi locus dataset. By doing so, it will be possible to better estimate the actual evolutionary lineages that compose the two species.

## Conclusions

This study constitutes another case study that underlines the interest and effectiveness of fast species discovery procedures; the achieved results are congruent and reliable, irrespective of the set of parameters and phylogenetic models applied, even though we only relied on a single locus dataset. Our results unravel at least three potential new species clusters (for *S*. *exigua*, *S*. *frugiperda* and *S*. *mauritia*) and also support the synonymy of *S*. *marima* with *S*. *ornithogalli*. Though these redefined species boundaries have to be considered cautiously, they nonetheless constitute a new line of evidence that may fuel the debate on *Spodoptera* species boundaries and inspire future studies on species such as the beet armyworm *S*. *exigua*, for which a potential cryptic diversity was not expected. In contrast, the case of *S*. *descoinsi* is clearly more complex as illustrated by the inconsistencies between the results of PTP and GMYC procedures. Here further studies relying on additional markers and species validation procedures will be necessary to precise the status of this species.

## Supporting Information

S1 Fig Phylogram (Best-fit maximum likelihood tree) resulting from the RAxML analyses. Support values are provided on major nodes (only BV ≥ 50% are shown).
(PDF)

S2 Fig Phylogram resulting from the MrBayes analyses. Support values are provided on major nodes (only PP ≥ 0.5 are shown).
(PDF)

S3 Fig Results of the PTP analyses based on the ML topology. Putative species clusters are indicated using transitions between black-coloured to red-coloured branches.
(PDF)

S4 Fig Results of the PTP analyses based on the BI topology. Putative species clusters are indicated using transitions between black-coloured to red-coloured branches.
(PDF)

S5 Fig Results of the GMYC analyses based on BEAST analyses with a strict clock model. Putative species clusters are indicated using transitions between black-coloured to red-coloured branches. The inter- and intraspecific portions of the tree are divided with a dotted line (95% confidence intervals are figured using thinner dotted lines).
(PDF)

S6 Fig Results of the GMYC analyses based on BEAST analyses with an UCLN clock model. Putative species clusters are indicated using transitions between black-coloured to red-coloured branches. The inter- and intraspecific portions of the tree are divided with a dotted line (95% confidence intervals are figured using thinner dotted lines).
(PDF)

S1 Table Taxon sampling. Sequences generated in a previous study of our research group are highlighted using ‘*’ whereas newly generated sequences are highlighted using ‘**’. The Democratic Republic of the Congo was abbreviated using DRC. When the country of origin is known we also provide the corresponding ISO 3166 alpha-3 code. We used the following abbreviations for the repository institutes and collections: **ACG**: Área de Conservación Guanacaste—Centro de Investigación y Estaciones Biológicas Programa de Educación Biológica, Costa Rica; **ANIC**: Australian National Insect Collection, Australia; **BIO**: Biodiversity Institute of Ontario, Canada; **BSCZ**: Bavarian State Collection of Zoology, Munich, Germany; **CBGP**: INRA—Centre de Biologie pour la Gestion des Populations, Montferrier/Lez, France; **CLS**: College of Life Science, Capital Normal University, Beijing, China; **CIRN**: CIRN, University of the Azores, Portugal; **CTAG**: Center for Theoretical and Applied Genetics, New Brunswick, USA; **EMBRAPA**: Embrapa National Soybean Research Center, Brazil; **FERA**: Fond and Environment Research Agency, York, UK; **GSA**: Graduate School of Agriculture, Kita-Ku, Japan; **HUT**: Hefei University of Technology, China; **LEC**: Lancaster Environment Centre, UK; **MNHN**: Muséum National d'Histoire Naturelle, Paris, France; **NIAES**: National Institute for Agro-Environmental Sciences, Tsukuba, Japan; **NIBGE**: National Institute for Biotechnology and Genetic Engineering, Pakistan; **NIMBB**: National Institute of Molecular Biology and Biotechnology, Los Banos, Philippines; **NRIC**: Natural Resources Inventory Center, Tsukuba, Japan; **PAU**: Punjab Agricultural University, India; **PSCAS**: Penn State College of Agricultural Sciences, USA; **RCG**: Research Collection of Theo Gruenewald, Germany; **RCH**: Research Collection of Alfred Haslberger, Munich, Germany; **RCJH**: Research Collection of D.H. Janzen & W. Hallwachs, USA; **RCW**: Research Collection of Jeremy deWaard, Guelph, Canada. **SI**: Smithsonian Institution, Washington, USA; **TAU**: Tamilnadu Agricultural University, India; **UBC**: University of British Columbia, Vancouver, Canada; **UFP**: Universidade Federal do Parana, Curitiba, Brazil; **UM**: University of Maryland, USA; **UR**: Université de Rouen, Rouen, France; **USDA**: USDA ARS, Gainesville, USA; **YU**: Yangzhou University, China; **ZIQ**: Zuhai Inspection and Quarantine bureau, China.
(DOCX)

S2 Table Results of PartitionFinder analyses for the COI dataset. Three partitions were specified (one per codon position). Selected models and partitions based on the AICc are figured on the left whereas selected models and partitions based on the BIC are figured on the right.
(DOCX)

S3 Table Putative species clusters. The geographic origin and assignment to putative species clusters is provided for each *Spodoptera* specimen. Similarly to the sidebars presented on Fig 2, we have used one column per set of analyses (PTP with RAxML, PTP with MrBayes, GMYC with BEAST (strict clock) and GMYC with BEAST (UCLN clock). For more clarity, putative species clusters also are highlighted using a combination of numbers and distinct shades of grey.
(DOCX)
