# Supplementary figures and images for: Phylogenetic Molecular Species Delimitations Unravel Potential New Species in the Pest Genus Spodoptera Guenée, 1852 (Lepidoptera, Noctuidae)
Source: PLoS One. 2015 Apr 8;10(4):e0122407. doi: 10.1371/journal.pone.0122407 (PMC4390195; doi:10.1371/journal.pone.0122407)

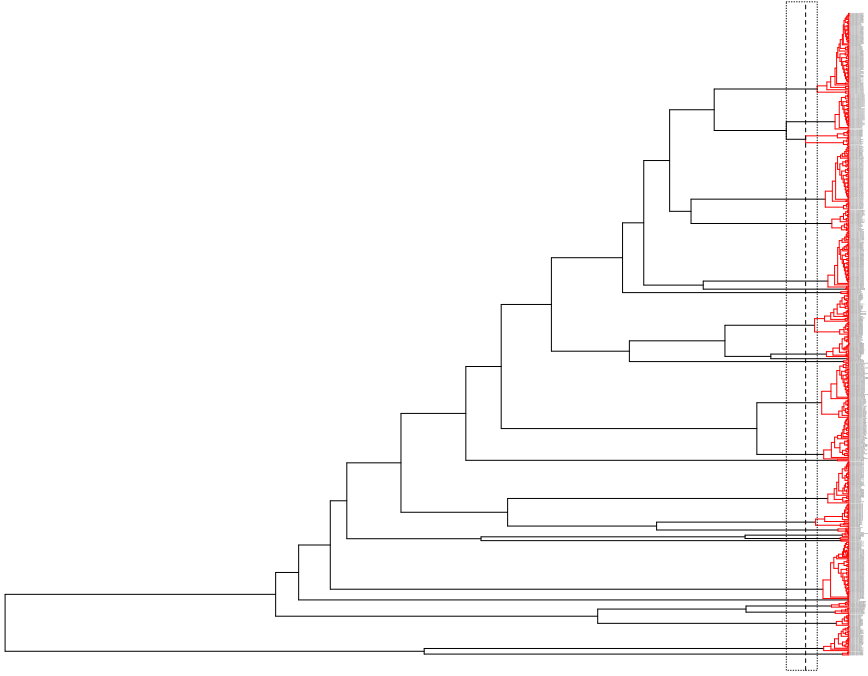

Supplement: S5 Fig — Putative species clusters are indicated using transitions between black-coloured to red-coloured branches. The inter- and intraspecific portions of the tree are divided with a dotted line (95% confidence intervals are figured using thinner dotted lines). (PDF) [file pone.0122407.s005.pdf]

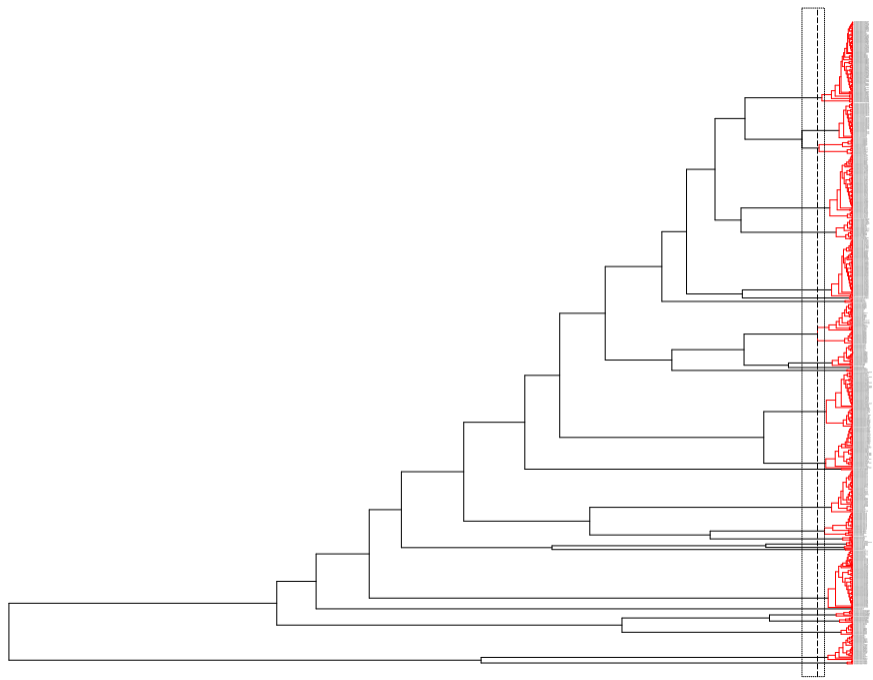

Supplement: S6 Fig — Putative species clusters are indicated using transitions between black-coloured to red-coloured branches. The inter- and intraspecific portions of the tree are divided with a dotted line (95% confidence intervals are figured using thinner dotted lines). (PDF) [file pone.0122407.s006.pdf]
