# Supplementary material for: Phylogenetic Molecular Species Delimitations Unravel Potential New Species in the Pest Genus Spodoptera Guenée, 1852 (Lepidoptera, Noctuidae)
Source: PLoS One. 2015 Apr 8;10(4):e0122407. doi: 10.1371/journal.pone.0122407 (PMC4390195; doi:10.1371/journal.pone.0122407)
Supplement: S1 Table — Sequences generated in a previous study of our research group are highlighted using ‘*’ whereas newly generated sequences are highlighted using ‘**’. The Democratic Republic of the Congo was abbreviated using DRC. When the country of origin is known we also provide the corresponding ISO 3166 alpha-3 code. We used the following abbreviations for the repository institutes and collections: ACG: Área de Conservación Guanacaste—Centro de Investigación y Estaciones Biológicas Programa de Educación Biológica, Costa Rica; ANIC: Australian National Insect Collection, Australia; BIO: Biodiversity Institute of Ontario, Canada; BSCZ: Bavarian State Collection of Zoology, Munich, Germany; CBGP: INRA—Centre de Biologie pour la Gestion des Populations, Montferrier/Lez, France; CLS: College of Life Science, Capital Normal University, Beijing, China; CIRN: CIRN, University of the Azores, Portugal; CTAG: Center for Theoretical and Applied Genetics, New Brunswick, USA; EMBRAPA: Embrapa National Soybean Research Center, Brazil; FERA: Fond and Environment Research Agency, York, UK; GSA: Graduate School of Agriculture, Kita-Ku, Japan; HUT: Hefei University of Technology, China; LEC: Lancaster Environment Centre, UK; MNHN: Muséum National d'Histoire Naturelle, Paris, France; NIAES: National Institute for Agro-Environmental Sciences, Tsukuba, Japan; NIBGE: National Institute for Biotechnology and Genetic Engineering, Pakistan; NIMBB: National Institute of Molecular Biology and Biotechnology, Los Banos, Philippines; NRIC: Natural Resources Inventory Center, Tsukuba, Japan; PAU: Punjab Agricultural University, India; PSCAS: Penn State College of Agricultural Sciences, USA; RCG: Research Collection of Theo Gruenewald, Germany; RCH: Research Collection of Alfred Haslberger, Munich, Germany; RCJH: Research Collection of D.H. Janzen & W. Hallwachs, USA; RCW: Research Collection of Jeremy deWaard, Guelph, Canada. SI: Smithsonian Institution, Washington, USA; TAU: Tamilnadu Agricultural University [file pone.0122407.s007.docx]

| **Repository institute / Collection** | **Species** | **Specimens / Voucher no.** | **Accession no.** | **Origin** | **ISO code** | **Reference study** |
| --- | --- | --- | --- | --- | --- | --- |
|  |  |  |  |  |  |  |
| CBGP | *Spodoptera albula* (Walker) | B20* | HQ177285 | French Guiana | GUF | Kergoat *et al.* 2012 |
| CBGP | *Spodoptera albula* (Walker) | B53* | HQ177286 | Honduras | HND | Kergoat *et al.* 2012 |
| CBGP | *Spodoptera albula* (Walker) | LSU16* | HQ177288 | French Guiana | GUF | Kergoat *et al.* 2012 |
| CBGP | *Spodoptera albula* (Walker) | LSU24* | HQ177289 | USA - Louisiana | USA | Kergoat *et al*. 2012 |
| MNHN | *Spodoptera albula* (Walker) | MNHN26** | KF854152 | Venezuela | VEN | this study |
| MNHN | *Spodoptera albula* (Walker) | MNHN27** | KF854153 | Jamaica | JAM | this study |
| MNHN | *Spodoptera albula* (Walker) | MNHN30** | KF854154 | Panama | PAN | this study |
| EMBRAPA | *Spodoptera albula* (Walker) | GBGL12664-13 | KF261137 | Brazil | BRA | Gomez-Rolim *et al.* 2013 |
| EMBRAPA | *Spodoptera albula* (Walker) | GBGL12663-13 | KF261138 | Brazil | BRA | Gomez-Rolim *et al*. 2013 |
| EMBRAPA | *Spodoptera albula* (Walker) | GBGL12662-13 | KF261139 | Brazil | BRA | Gomez-Rolim *et al*. 2013 |
| EMBRAPA | *Spodoptera albula* (Walker) | GBGL12661-13 | KF261140 | Brazil | BRA | Gomez-Rolim *et al*. 2013 |
| EMBRAPA | *Spodoptera albula* (Walker) | GBGL12660-13 | KF261141 | Brazil | BRA | Gomez-Rolim *et al*. 2013 |
| EMBRAPA | *Spodoptera albula* (Walker) | GBGL12659-13 | KF261142 | Brazil | BRA | Gomez-Rolim *et al*. 2013 |
| EMBRAPA | *Spodoptera albula* (Walker) | GBGL12658-13 | KF261143 | Brazil | BRA | Gomez-Rolim *et al*. 2013 |
| EMBRAPA | *Spodoptera albula* (Walker) | GBGL12657-13 | KF261144 | Brazil | BRA | Gomez-Rolim *et al*. 2013 |
| EMBRAPA | *Spodoptera albula* (Walker) | GBGL12656-13 | KF261145 | Brazil | BRA | Gomez-Rolim *et al*. 2013 |
| EMBRAPA | *Spodoptera albula* (Walker) | GBGL12655-13 | KF261146 | Brazil | BRA | Gomez-Rolim *et al*. 2013 |
| EMBRAPA | *Spodoptera albula* (Walker) | GBGL12654-13 | KF261147 | Brazil | BRA | Gomez-Rolim *et al*. 2013 |
| EMBRAPA | *Spodoptera albula* (Walker) | GBGL12653-13 | KF261148 | Brazil | BRA | Gomez-Rolim *et al*. 2013 |
| EMBRAPA | *Spodoptera albula* (Walker) | GBGL12652-13 | KF261149 | Brazil | BRA | Gomez-Rolim *et al*. 2013 |
| EMBRAPA | *Spodoptera albula* (Walker) | GBGL12651-13 | KF261150 | Brazil | BRA | Gomez-Rolim *et al*. 2013 |
| EMBRAPA | *Spodoptera albula* (Walker) | GBGL12650-13 | KF261151 | Brazil | BRA | Gomez-Rolim *et al*. 2013 |
| EMBRAPA | *Spodoptera albula* (Walker) | GBGL12649-13 | KF261152 | Brazil | BRA | Gomez-Rolim *et al*. 2013 |
| EMBRAPA | *Spodoptera albula* (Walker) | GBGL12648-13 | KF261153 | Brazil | BRA | Gomez-Rolim *et al*. 2013 |
| EMBRAPA | *Spodoptera albula* (Walker) | GBGL12647-13 | KF261154 | Brazil | BRA | Gomez-Rolim *et al*. 2013 |
| CBGP | *Spodoptera androgea* (Stoll) | B1* | HQ177290 | Guadeloupe | GLP | Kergoat *et al*. 2012 |
| MNHN | *Spodoptera androgea* (Stoll) | MNHN20** | KF854155 | Dominican Republic | DOM | this study |
| MNHN | *Spodoptera androgea* (Stoll) | MNHN21** | KF854156 | Panama | PAN | this study |
| MNHN | *Spodoptera androgea* (Stoll) | MNHN22** | KF854157 | Ecuador | ECU | this study |
| MNHN | *Spodoptera androgea* (Stoll) | MNHN23** | KF854158 | Martinique | MTQ | this study |
| MNHN | *Spodoptera androgea* (Stoll) | MNHN24** | KF854159 | Peru | PER | this study |
| MNHN | *Spodoptera androgea* (Stoll) | MNHN66** | KF854160 | Dominican Republic | DOM | this study |
| PSCAS | *Spodoptera androgea* (Stoll) | MHAUB900-05 | GU159405 | Costa Rica | CRI |  |
| PSCAS | *Spodoptera androgea* (Stoll) | MHAUB901-05 | GU159406 | Costa Rica | CRI |  |
| PSCAS | *Spodoptera androgea* (Stoll) | MHAUB903-05 | GU159407 | Costa Rica | CRI |  |
| PSCAS | *Spodoptera androgea* (Stoll) | MHAUB907-05 | GU159408 | Costa Rica | CRI |  |
| PSCAS | *Spodoptera androgea* (Stoll) | MHAUB902-05 | GU159409 | Costa Rica | CRI |  |
| PSCAS | *Spodoptera androgea* (Stoll) | MHAUB905-05 | GU159410 | Costa Rica | CRI |  |
| PSCAS | *Spodoptera androgea* (Stoll) | MHAUB904-05 | GU159411 | Costa Rica | CRI |  |
| PSCAS | *Spodoptera androgea* (Stoll) | MHAUB906-05 | GU159412 | Costa Rica | CRI |  |
| PSCAS | *Spodoptera androgea* (Stoll) | MHAUB908-05 | GU159413 | Costa Rica | CRI |  |
| PSCAS | *Spodoptera androgea* (Stoll) | MHAUB909-05 | GU159414 | Costa Rica | CRI |  |
| PSCAS | *Spodoptera androgea* (Stoll) | MHAUF370-06 | GU163696 | Costa Rica | CRI |  |
| RCJH | *Spodoptera androgea* (Stoll) | MHMXV046-08 | JQ537729 | Costa Rica | CRI |  |
| ACG | *Spodoptera androgea* (Stoll) | BLPDA435-09 | JQ550995 | Costa Rica | CRI |  |
| ACG | *Spodoptera androgea* (Stoll) | BLPDA718-09 | JQ551244 | Costa Rica | CRI |  |
| ACG | *Spodoptera androgea* (Stoll) | BLPDC921-09 | JQ553063 | Costa Rica | CRI |  |
| ACG | *Spodoptera androgea* (Stoll) | BLPDD477-09 | JQ553521 | Costa Rica | CRI |  |
| ACG | *Spodoptera androgea* (Stoll) | BLPDD478-09 | JQ553522 | Costa Rica | CRI |  |
| ACG | *Spodoptera androgea* (Stoll) | BLPDD685-09 | JQ553704 | Costa Rica | CRI |  |
| ACG | *Spodoptera androgea* (Stoll) | BLPDD686-09 | JQ553705 | Costa Rica | CRI |  |
| ACG | *Spodoptera androgea* (Stoll) | BLPCI618-08 | JQ554809 | Costa Rica | CRI |  |
| ACG | *Spodoptera androgea* (Stoll) | BLPCJ205-08 | JQ555319 | Costa Rica | CRI |  |
| RCJH | *Spodoptera androgea* (Stoll) | BLPCL009-08 | JQ556959 | Costa Rica | CRI |  |
| RCJH | *Spodoptera androgea* (Stoll) | BLPCL010-08 | JQ556960 | Costa Rica | CRI |  |
| ACG | *Spodoptera androgea* (Stoll) | BLPCO066-08 | JQ559209 | Costa Rica | CRI |  |
| ACG | *Spodoptera androgea* (Stoll) | BLPCC488-08 | JQ559603 | Costa Rica | CRI |  |
| ACG | *Spodoptera androgea* (Stoll) | BLPCD471-08 | JQ560333 | Costa Rica | CRI |  |
| ACG | *Spodoptera androgea* (Stoll) | BLPCD475-08 | JQ560337 | Costa Rica | CRI |  |
| ACG | *Spodoptera androgea* (Stoll) | BLPCF454-08 | JQ562001 | Costa Rica | CRI |  |
| ACG | *Spodoptera androgea* (Stoll) | BLPCF455-08 | JQ562002 | Costa Rica | CRI |  |
| ACG | *Spodoptera androgea* (Stoll) | BLPCF456-08 | JQ562003 | Costa Rica | CRI |  |
| ACG | *Spodoptera androgea* (Stoll) | BLPBF046-07 | JQ565383 | Costa Rica | CRI |  |
| ACG | *Spodoptera androgea* (Stoll) | BLPBF047-07 | JQ565384 | Costa Rica | CRI |  |
| ACG | *Spodoptera androgea* (Stoll) | BLPBF048-07 | JQ565385 | Costa Rica | CRI |  |
| ACG | *Spodoptera androgea* (Stoll) | BLPBF049-07 | JQ565386 | Costa Rica | CRI |  |
| ACG | *Spodoptera androgea* (Stoll) | BLPCC003-08 | JQ569210 | Costa Rica | CRI |  |
| ACG | *Spodoptera androgea* (Stoll) | BLPCC004-08 | JQ569211 | Costa Rica | CRI |  |
| ACG | *Spodoptera androgea* (Stoll) | BLPAE060-06 | JQ569842 | Costa Rica | CRI |  |
| ACG | *Spodoptera androgea* (Stoll) | BLPAE061-06 | JQ569843 | Costa Rica | CRI |  |
| ACG | *Spodoptera androgea* (Stoll) | BLPAG449-07 | JQ571472 | Costa Rica | CRI |  |
| ACG | *Spodoptera androgea* (Stoll) | BLPBB638-07 | JQ573138 | Costa Rica | CRI |  |
| ACG | *Spodoptera androgea* (Stoll) | BLPBC143-07 | JQ573262 | Costa Rica | CRI |  |
| ACG | *Spodoptera androgea* (Stoll) | BLPBD224-07 | JQ574067 | Costa Rica | CRI |  |
| ACG | *Spodoptera androgea* (Stoll) | BLPAA821-06 | JQ577747 | Costa Rica | CRI |  |
| ACG | *Spodoptera androgea* (Stoll) | BLPAA925-06 | JQ577819 | Costa Rica | CRI |  |
| ACG | *Spodoptera androgea* (Stoll) | BLPAB812-06 | JQ578479 | Costa Rica | CRI |  |
| ACG | *Spodoptera androgea* (Stoll) | BLPAB894-06 | JQ578526 | Costa Rica | CRI |  |
| ACG | *Spodoptera androgea* (Stoll) | BLPAC245-06 | JQ578738 | Costa Rica | CRI |  |
| ACG | *Spodoptera androgea* (Stoll) | BLPAC246-06 | JQ578739 | Costa Rica | CRI |  |
| ACG | *Spodoptera androgea* (Stoll) | BLPAC327-06.COI | JQ578766 | Costa Rica | CRI |  |
| ACG | *Spodoptera androgea* (Stoll) | BLPAC726-06 | JQ579067 | Costa Rica | CRI |  |
| PSCAS | *Spodoptera androgea* (Stoll) | MHMXA727-06 | JQ602908 | Costa Rica | CRI |  |
| PSCAS | *Spodoptera androgea* (Stoll) | MHMXA730-06 | JQ602910 | Costa Rica | CRI |  |
| PSCAS | *Spodoptera androgea* (Stoll) | MHMXD719-06 | JQ604384 | Costa Rica | CRI |  |
| ANIC | *Spodoptera apertura* (Walker) | ANICK313-10 | KF388225 | Australia | AUS | Hebert *et al.* 2013 |
| ANIC | *Spodoptera apertura* (Walker) | ANICK315-10 | KF388292 | Australia | AUS | Hebert *et al.* 2013 |
| CBGP | *Spodoptera cilium* Guenée | GK123* | HQ177292 | Kenya | KEN | Kergoat *et al.* 2012 |
| CBGP | *Spodoptera cilium* Guenée | GK124* | HQ177293 | Kenya | KEN | Kergoat *et al.* 2012 |
| CBGP | *Spodoptera cilium* Guenée | GK125* | HQ177294 | Kenya | KEN | Kergoat *et al*. 2012 |
| NIAES | *Spodoptera cilium* Guenée | NIAES:LEPID:173 | AB735234 | Japan | JAP |  |
| NIAES | *Spodoptera cilium* Guenée | NIAES:LEPID:175 | AB735235 | Japan | JAP |  |
| NIAES | *Spodoptera cilium* Guenée | NIAES:LEPID:176 | AB735236 | Japan | JAP |  |
| NIAES | *Spodoptera cilium* Guenée | NIAES:LEPID:177 | AB735237 | Japan | JAP |  |
| NIBGE | *Spodoptera cilium* Guenée | NIAES:LEPID:178 | AB735238 | Japan | JAP |  |
| NIBGE | *Spodoptera cilium* Guenée | MAMOT956-10 | JN988597 | Pakistan | PAK |  |
| NIBGE | *Spodoptera cilium* Guenée | MAMOT955-10 | JN988598 | Pakistan | PAK |  |
| NIBGE | *Spodoptera cilium* Guenée | MAMOT954-10 | JN988599 | Pakistan | PAK |  |
| NIBGE | *Spodoptera cilium* Guenée | MAMOT953-10 | JN988600 | Pakistan | PAK |  |
| CBGP | *Spodoptera cosmiodes* (Walker) | B100* | HQ177297 | French Guiana | GUF | Kergoat *et al*. 2012 |
| CBGP | *Spodoptera cosmiodes* (Walker) | B150* | HQ177298 | Brazil | BRA | Kergoat *et al*. 2012 |
| CBGP | *Spodoptera cosmiodes* (Walker) | B151* | HQ177299 | Brazil | BRA | Kergoat *et al*. 2012 |
| CBGP | *Spodoptera cosmiodes* (Walker) | B166* | HQ177300 | Brazil | BRA | Kergoat *et al*. 2012 |
| CBGP | *Spodoptera cosmiodes* (Walker) | B19* | HQ177295 | French Guiana | GUF | Kergoat *et al*. 2012 |
| UFP | *Spodoptera cosmiodes* (Walker) | LEMMZ123-10 | JF854736 | Brazil | BRA |  |
| UFP | *Spodoptera cosmiodes* (Walker) | LEMMZ126-10 | JF854739 | Brazil | BRA |  |
| EMBRAPA | *Spodoptera cosmiodes* (Walker) | GBGL12629-13 | KF261172 | Brazil | BRA | Gomez-Rolim *et al*. 2013 |
| EMBRAPA | *Spodoptera cosmiodes* (Walker) | GBGL12628-13 | KF261173 | Brazil | BRA | Gomez-Rolim *et al*. 2013 |
| EMBRAPA | *Spodoptera cosmiodes* (Walker) | GBGL12627-13 | KF261174 | Brazil | BRA | Gomez-Rolim *et al*. 2013 |
| EMBRAPA | *Spodoptera cosmiodes* (Walker) | GBGL12626-13 | KF261175 | Brazil | BRA | Gomez-Rolim *et al*. 2013 |
| EMBRAPA | *Spodoptera cosmiodes* (Walker) | GBGL12625-13 | KF261176 | Brazil | BRA | Gomez-Rolim *et al*. 2013 |
| EMBRAPA | *Spodoptera cosmiodes* (Walker) | GBGL12624-13 | KF261177 | Brazil | BRA | Gomez-Rolim et al. 2013 |
| EMBRAPA | *Spodoptera cosmiodes* (Walker) | GBGL12623-13 | KF261178 | Brazil | BRA | Gomez-Rolim *et al*. 2013 |
| EMBRAPA | *Spodoptera cosmiodes* (Walker) | GBGL12622-13 | KF261179 | Brazil | BRA | Gomez-Rolim *et al*. 2013 |
| EMBRAPA | *Spodoptera cosmiodes* (Walker) | GBGL12621-13 | KF261180 | Brazil | BRA | Gomez-Rolim *et al*. 2013 |
| EMBRAPA | *Spodoptera cosmiodes* (Walker) | GBGL12619-13 | KF261182 | Brazil | BRA | Gomez-Rolim *et al*. 2013 |
| EMBRAPA | *Spodoptera cosmiodes* (Walker) | GBGL12618-13 | KF261183 | Brazil | BRA | Gomez-Rolim *et al*. 2013 |
| EMBRAPA | *Spodoptera cosmiodes* (Walker) | GBGL12617-13 | KF261184 | Brazil | BRA | Gomez-Rolim *et al*. 2013 |
| EMBRAPA | *Spodoptera cosmiodes* (Walker) | GBGL12616-13 | KF261185 | Brazil | BRA | Gomez-Rolim *et al*. 2013 |
| EMBRAPA | *Spodoptera cosmiodes* (Walker) | GBGL12615-13 | KF261186 | Brazil | BRA | Gomez-Rolim *et al*. 2013 |
| EMBRAPA | *Spodoptera cosmiodes* (Walker) | GBGL12614-13 | KF261187 | Brazil | BRA | Gomez-Rolim *et al*. 2013 |
| EMBRAPA | *Spodoptera cosmiodes* (Walker) | GBGL12613-13 | KF261188 | Brazil | BRA | Gomez-Rolim *et al*. 2013 |
| EMBRAPA | *Spodoptera cosmiodes* (Walker) | GBGL12612-13 | KF261189 | Brazil | BRA | Gomez-Rolim *et al*. 2013 |
| EMBRAPA | *Spodoptera cosmiodes* (Walker) | GBGL12611-13 | KF261190 | Brazil | BRA | Gomez-Rolim *et al*. 2013 |
| EMBRAPA | *Spodoptera cosmiodes* (Walker) | GBGL12610-13 | KF261191 | Brazil | BRA | Gomez-Rolim *et al*. 2013 |
| EMBRAPA | *Spodoptera cosmiodes* (Walker) | GBGL12609-13 | KF261192 | Brazil | BRA | Gomez-Rolim *et al*. 2013 |
| EMBRAPA | *Spodoptera cosmiodes* (Walker) | GBGL12608-13 | KF261193 | Brazil | BRA | Gomez-Rolim *et al*. 2013 |
| EMBRAPA | *Spodoptera cosmiodes* (Walker) | GBGL12607-13 | KF261194 | Brazil | BRA | Gomez-Rolim *et al*. 2013 |
| EMBRAPA | *Spodoptera cosmiodes* (Walker) | GBGL12606-13 | KF261195 | Brazil | BRA | Gomez-Rolim *et al*. 2013 |
| EMBRAPA | *Spodoptera cosmiodes* (Walker) | GBGL12605-13 | KF261196 | Brazil | BRA | Gomez-Rolim *et al*. 2013 |
| EMBRAPA | *Spodoptera cosmiodes* (Walker) | GBGL12604-13 | KF261197 | Brazil | BRA | Gomez-Rolim *et al*. 2013 |
| EMBRAPA | *Spodoptera cosmiodes* (Walker) | GBGL12603-13 | KF261198 | Brazil | BRA | Gomez-Rolim *et al*. 2013 |
| EMBRAPA | *Spodoptera cosmiodes* (Walker) | GBGL12602-13 | KF261199 | Brazil | BRA | Gomez-Rolim *et al*. 2013 |
| EMBRAPA | *Spodoptera cosmiodes* (Walker) | GBGL12601-13 | KF261200 | Brazil | BRA | Gomez-Rolim *et al*. 2013 |
| CBGP | *Spodoptera descoinsi* L.-C. & Silvain | B102* | HQ177306 | French Guiana | GUF | Kergoat *et al*. 2012 |
| CBGP | *Spodoptera descoinsi* L.-C. & Silvain | B157* | HQ177307 | French Guiana | GUF | Kergoat *et al*. 2012 |
| CBGP | *Spodoptera descoinsi* L.-C. & Silvain | B32* | HQ177304 | French Guiana | GUF | Kergoat *et al*. 2012 |
| CBGP | *Spodoptera descoinsi* L.-C. & Silvain | B38* | HQ177305 | French Guiana | GUF | Kergoat *et al*. 2012 |
| CBGP | *Spodoptera descoinsi* L.-C. & Silvain | LSU30* | HQ177308 | French Guiana | GUF | Kergoat *et al*. 2012 |
| CBGP | *Spodoptera descoinsi* L.-C. & Silvain | LSU31* | HQ177309 | French Guiana | GUF | Kergoat *et al*. 2012 |
| MNHN | *Spodoptera descoinsi* L.-C. & Silvain | MNHN43** | KF854162 | French Guiana | GUF | this study |
| MNHN | *Spodoptera descoinsi* L.-C. & Silvain | MNHN44** | KF854163 | French Guiana | GUF | this study |
| MNHN | *Spodoptera descoinsi* L.-C. & Silvain | MNHN45** | KF854164 | French Guiana | GUF | this study |
| CBGP | *Spodoptera depravata* (Butler) | GK206* | HQ177302 | Japan | JAP | Kergoat *et al*. 2012 |
| NIAES | *Spodoptera depravata* (Butler) | NIAES:LEPID:210 | AB733681 | Japan | JAP |  |
| NIAES | *Spodoptera depravata* (Butler) | NIAES:LEPID:211 | AB733682 | Japan | JAP |  |
| CLS | *Spodoptera depravata* (Butler) | JF100809166 | JX509780 | China | CHN |  |
| CLS | *Spodoptera depravata* (Butler) | LJZ100726594 | JX509781 | China | CHN |  |
| CLS | *Spodoptera depravata* (Butler) | LJZ100726665 | JX509782 | China | CHN |  |
| UM | *Spodoptera depravata* (Butler) | LTOLB1216-11 | KF492138 | Japan | JAP |  |
| CBGP | *Spodoptera dolichos* (Fabricius) | B104* | HQ177315 | Guadeloupe | GLP | Kergoat *et al*. 2012 |
| CBGP | *Spodoptera dolichos* (Fabricius) | B122* | HQ177316 | USA - Georgia | USA | Kergoat *et al*. 2012 |
| CBGP | *Spodoptera dolichos* (Fabricius) | B35* | HQ177313 | French Guiana | GUF | Kergoat *et al*. 2012 |
| CBGP | *Spodoptera dolichos* (Fabricius) | B37* | HQ177314 | Guadeloupe | GLP | Kergoat *et al*. 2012 |
| CBGP | *Spodoptera dolichos* (Fabricius) | LSU14* | HQ177318 | French Guiana | GUF | Kergoat *et al*. 2012 |
| CBGP | *Spodoptera dolichos* (Fabricius) | LSU27* | HQ177319 | USA - Florida | USA | Kergoat *et al*. 2012 |
| CBGP | *Spodoptera dolichos* (Fabricius) | LSU3* | HQ177317 | Guadeloupe | GLP | Kergoat *et al*. 2012 |
| MNHN | *Spodoptera dolichos* (Fabricius) | MNHN34** | KF854165 | Dominican Republic | DOM | this study |
| MNHN | *Spodoptera dolichos* (Fabricius) | MNHN35** | KF854166 | Venezuela | VEN | this study |
| MNHN | *Spodoptera dolichos* (Fabricius) | MNHN36** | KF854167 | French Guiana | GUF | this study |
| MNHN | *Spodoptera dolichos* (Fabricius) | MNHN37** | KF854168 | Jamaica | JAM | this study |
| MNHN | *Spodoptera dolichos* (Fabricius) | MNHN38** | KF854169 | Martinique | MTQ | this study |
| MNHN | *Spodoptera dolichos* (Fabricius) | MNHN65** | KF854170 | Peru | PER | this study |
| MNHN | *Spodoptera dolichos* (Fabricius) | MNHN7** | KF854171 | Martinique | MTQ | this study |
| MNHN | *Spodoptera dolichos* (Fabricius) | MNHN8** | KF854172 | Martinique | MTQ | this study |
| PSCAS | *Spodoptera dolichos* (Fabricius) | MHAUB912-05 | GU159415 | Costa Rica | CRI |  |
| PSCAS | *Spodoptera dolichos* (Fabricius) | MHAUB911-05 | GU159416 | Costa Rica | CRI |  |
| PSCAS | *Spodoptera dolichos* (Fabricius) | MHAUB910-05 | GU159417 | Costa Rica | CRI |  |
| PSCAS | *Spodoptera dolichos* (Fabricius) | MHAUB914-05 | GU159418 | Costa Rica | CRI |  |
| PSCAS | *Spodoptera dolichos* (Fabricius) | MHAUB915-05 | GU159419 | Costa Rica | CRI |  |
| PSCAS | *Spodoptera dolichos* (Fabricius) | MHAUB913-05 | GU159420 | Costa Rica | CRI |  |
| PSCAS | *Spodoptera dolichos* (Fabricius) | MHAUF369-06 | GU163697 | Costa Rica | CRI |  |
| PSCAS | *Spodoptera dolichos* (Fabricius) | MHAUC210-06 | GU337021 | Costa Rica | CRI |  |
| USDA | *Spodoptera dolichos* (Fabricius) | GBGL10124-12 | HM756086 | USA - Florida | USA | Nagoshi *et al*. 2011 |
| USDA | *Spodoptera dolichos* (Fabricius) | GBGL10125-12 | HM756087 | USA - Florida | USA | Nagoshi *et al*. 2011 |
| USDA | *Spodoptera dolichos* (Fabricius) | GBGL10126-12 | HM756088 | USA - Florida | USA | Nagoshi *et al*. 2011 |
| USDA | *Spodoptera dolichos* (Fabricius) | GBGL10127-12 | HM756089 | USA - Florida | USA | Nagoshi *et al*. 2011 |
| CBGP | *Spodoptera dolichos* (Fabricius) | B2* | HQ177312 | French Guiana | GUF | Kergoat *et al.* 2012 |
| CBGP | *Spodoptera dolichos* (Fabricius) | LSU29* | HQ177320 | French Guiana | GUF | Kergoat *et al.* 2012 |
| UFP | *Spodoptera dolichos* (Fabricius) | LEMMZ124-10 | JF854737 | Brazil | BRA |  |
| ACG | *Spodoptera dolichos* (Fabricius) | BLPAG055-07 | JN807244 | Costa Rica | CRI |  |
| RCJH | *Spodoptera dolichos* (Fabricius) | MHMXQ639-08 | JQ536602 | Costa Rica | CRI |  |
| ACG | *Spodoptera dolichos* (Fabricius) | BLPCP113-08 | JQ550106 | Costa Rica | CRI |  |
| ACG | *Spodoptera dolichos* (Fabricius) | BLPDB841-09 | JQ552123 | Costa Rica | CRI |  |
| ACG | *Spodoptera dolichos* (Fabricius) | BLPDC205-09 | JQ552408 | Costa Rica | CRI |  |
| ACG | *Spodoptera dolichos* (Fabricius) | BLPCI619-08 | JQ554810 | Costa Rica | CRI |  |
| ACG | *Spodoptera dolichos* (Fabricius) | BLPCJ098-08 | JQ555213 | Costa Rica | CRI |  |
| RCJH | *Spodoptera dolichos* (Fabricius) | BLPCK133-08 | JQ556135 | Costa Rica | CRI |  |
| ACG | *Spodoptera dolichos* (Fabricius) | BLPCO135-08 | JQ559274 | Costa Rica | CRI |  |
| ACG | *Spodoptera dolichos* (Fabricius) | BLPCD470-08 | JQ560332 | Costa Rica | CRI |  |
| ACG | *Spodoptera dolichos* (Fabricius) | BLPCD472-08 | JQ560334 | Costa Rica | CRI |  |
| ACG | *Spodoptera dolichos* (Fabricius) | BLPCD473-08 | JQ560335 | Costa Rica | CRI |  |
| ACG | *Spodoptera dolichos* (Fabricius) | BLPCD476-08 | JQ560338 | Costa Rica | CRI |  |
| ACG | *Spodoptera dolichos* (Fabricius) | BLPCD478-08 | JQ560340 | Costa Rica | CRI |  |
| ACG | *Spodoptera dolichos* (Fabricius) | BLPCH049-08 | JQ563387 | Costa Rica | CRI |  |
| ACG | *Spodoptera dolichos* (Fabricius) | BLPBG173-07 | JQ566273 | Costa Rica | CRI |  |
| ACG | *Spodoptera dolichos* (Fabricius) | BLPBH662-07 | JQ567479 | Costa Rica | CRI |  |
| ACG | *Spodoptera dolichos* (Fabricius) | BLPBH664-07 | JQ567481 | Costa Rica | CRI |  |
| ACG | *Spodoptera dolichos* (Fabricius) | BLPAF454-07 | JQ570808 | Costa Rica | CRI |  |
| ACG | *Spodoptera dolichos* (Fabricius) | BLPAF455-07 | JQ570809 | Costa Rica | CRI |  |
| ACG | *Spodoptera dolichos* (Fabricius) | BLPAF456-07 | JQ570810 | Costa Rica | CRI |  |
| ACG | *Spodoptera dolichos* (Fabricius) | BLPAG030-07 | JQ571203 | Costa Rica | CRI |  |
| ACG | *Spodoptera dolichos* (Fabricius) | BLPAH330-07 | JQ571984 | Costa Rica | CRI |  |
| ACG | *Spodoptera dolichos* (Fabricius) | BLPAH331-07 | JQ571985 | Costa Rica | CRI |  |
| ACG | *Spodoptera dolichos* (Fabricius) | BLPBA273-07 | JQ572301 | Costa Rica | CRI |  |
| ACG | *Spodoptera dolichos* (Fabricius) | BLPAA820-06 | JQ577746 | Costa Rica | CRI |  |
| ACG | *Spodoptera dolichos* (Fabricius) | BLPAA926-06 | JQ577820 | Costa Rica | CRI |  |
| ACG | *Spodoptera dolichos* (Fabricius) | BLPAA927-06 | JQ577821 | Costa Rica | CRI |  |
| ACG | *Spodoptera dolichos* (Fabricius) | BLPAB037-06 | JQ577861 | Costa Rica | CRI |  |
| ACG | *Spodoptera dolichos* (Fabricius) | BLPAB238-06 | JQ578017 | Costa Rica | CRI |  |
| ACG | *Spodoptera dolichos* (Fabricius) | BLPAB239-06 | JQ578018 | Costa Rica | CRI |  |
| ACG | *Spodoptera dolichos* (Fabricius) | BLPAB275-06 | JQ578049 | Costa Rica | CRI |  |
| ACG | *Spodoptera dolichos* (Fabricius) | BLPAB276-06 | JQ578050 | Costa Rica | CRI |  |
| ACG | *Spodoptera dolichos* (Fabricius) | BLPAC328-06 | JQ578767 | Costa Rica | CRI |  |
| ACG | *Spodoptera dolichos* (Fabricius) | BLPAD217-06 | JQ579336. | Costa Rica | CRI |  |
| PSCAS | *Spodoptera dolichos* (Fabricius) | MHMXO341-08 | JQ601917 | Costa Rica | CRI |  |
| PSCAS | *Spodoptera dolichos* (Fabricius) | MHMXA733-06 | JQ602911 | Costa Rica | CRI |  |
| CBGP | *Spodoptera eridania* (Stoll) | B108* | HQ177328 | Peru | PER | Kergoat *et al.* 2012 |
| CBGP | *Spodoptera eridania* (Stoll) | B109* | HQ177329 | Peru | PER | Kergoat *et al.* 2012 |
| CBGP | *Spodoptera eridania* (Stoll) | B16* | HQ177321 | USA - Louisiana | USA | Kergoat *et al.* 2012 |
| CBGP | *Spodoptera eridania* (Stoll) | B47* | HQ177322 | Guadeloupe | GLP | Kergoat *et al.* 2012 |
| CBGP | *Spodoptera eridania* (Stoll) | B70* | HQ177323 | USA - Louisiana | USA | Kergoat *et al.* 2012 |
| CBGP | *Spodoptera eridania* (Stoll) | B71* | HQ177324 | USA - Louisiana | USA | Kergoat *et al.* 2012 |
| CBGP | *Spodoptera eridania* (Stoll) | B72* | HQ177325 | USA - Louisiana | USA | Kergoat *et al.* 2012 |
| CBGP | *Spodoptera eridania* (Stoll) | B73* | HQ177326 | USA - Louisiana | USA | Kergoat *et al.* 2012 |
| CBGP | *Spodoptera eridania* (Stoll) | B75* | HQ177327 | Guadeloupe | GLP | Kergoat *et al.* 2012 |
| USDA | *Spodoptera eridania* (Stoll) | GBGL10119-12 | HM756081 | USA - Florida | USA | Nagoshi *et al.* 2011 |
| USDA | *Spodoptera eridania* (Stoll) | GBGL10120-12 | HM756082 | USA - Florida | USA | Nagoshi et al. 2011 |
| USDA | *Spodoptera eridania* (Stoll) | GBGL10121-12 | HM756083 | USA - Florida | USA | Nagoshi *et al.* 2011 |
| USDA | *Spodoptera eridania* (Stoll) | GBGL10122-12 | HM756084 | USA - Florida | USA | Nagoshi *et al.* 2011 |
| USDA | *Spodoptera eridania* (Stoll) | GBGL10123-12 | HM756085 | USA - Florida | USA | Nagoshi *et al.* 2011 |
| CBGP | *Spodoptera eridania* (Stoll) | LSU5* | HQ177330 | USA | USA | Kergoat *et al*. 2012 |
| PSCAS | *Spodoptera eridania* (Stoll) | MHAUB899-05 | GU159421 | Costa Rica | CRI |  |
| PSCAS | *Spodoptera eridania* (Stoll) | MHAUB898-05 | GU159422 | Costa Rica | CRI |  |
| PSCAS | *Spodoptera eridania* (Stoll) | MHAUB897-05 | GU159423 | Costa Rica | CRI |  |
| PSCAS | *Spodoptera eridania* (Stoll) | MHAUB895-05 | GU159424 | Costa Rica | CRI |  |
| PSCAS | *Spodoptera eridania* (Stoll) | MHAUB896-05 | GU159425 | Costa Rica | CRI |  |
| RCJH | *Spodoptera eridania* (Stoll) | BLPCK258-08 | JN807245 | Costa Rica | CRI |  |
| ACG | *Spodoptera eridania* (Stoll) | BLPEC679-11 | JQ546665 | Costa Rica | CRI |  |
| ACG | *Spodoptera eridania* (Stoll) | BLPDA469-09 | JQ551023 | Costa Rica | CRI |  |
| ACG | *Spodoptera eridania* (Stoll) | BLPDC450-09 | JQ552635 | Costa Rica | CRI |  |
| PSCAS | *Spodoptera eridania* (Stoll) | MHMXF638-07 | JQ605007 | Costa Rica | CRI |  |
| PSCAS | *Spodoptera eridania* (Stoll) | MHMXI219-07 | JQ605405 | Costa Rica | CRI |  |
| EMBRAPA | *Spodoptera eridania* (Stoll) | GBGL12646-13 | KF261155 | Brazil | BRA | Gomez-Rolim *et al*. 2013 |
| EMBRAPA | *Spodoptera eridania* (Stoll) | GBGL12645-13 | KF261156 | Brazil | BRA | Gomez-Rolim *et al*. 2013 |
| EMBRAPA | *Spodoptera eridania* (Stoll) | GBGL12644-13 | KF261157 | Brazil | BRA | Gomez-Rolim *et al*. 2013 |
| UFP | *Spodoptera eridania* (Stoll) | LEMMZ129-10 | JF854742 | Brazil | BRA |  |
| EMBRAPA | *Spodoptera eridania* (Stoll) | GBGL12643-13 | KF261158 | Brazil | BRA | Gomez-Rolim *et al*. 2013 |
| EMBRAPA | *Spodoptera eridania* (Stoll) | GBGL12642-13 | KF261159 | Brazil | BRA | Gomez-Rolim *et al*. 2013 |
| EMBRAPA | *Spodoptera eridania* (Stoll) | GBGL12641-13 | KF261160 | Brazil | BRA |  |
| EMBRAPA | *Spodoptera eridania* (Stoll) | GBGL12640-13 | KF261161 | Brazil | BRA |  |
| EMBRAPA | *Spodoptera eridania* (Stoll) | GBGL12639-13 | KF261162 | Brazil | BRA |  |
| EMBRAPA | *Spodoptera eridania* (Stoll) | GBGL12638-13 | KF261163 | Brazil | BRA |  |
| EMBRAPA | *Spodoptera eridania* (Stoll) | GBGL12637-13 | KF261164 | Brazil | BRA |  |
| EMBRAPA | *Spodoptera eridania* (Stoll) | GBGL12636-13 | KF261165 | Brazil | BRA |  |
| EMBRAPA | *Spodoptera eridania* (Stoll) | GBGL12635-13 | KF261166 | Brazil | BRA |  |
| EMBRAPA | *Spodoptera eridania* (Stoll) | GBGL12634-13 | KF261167 | Brazil | BRA |  |
| EMBRAPA | *Spodoptera eridania* (Stoll) | GBGL12633-13 | KF261168 | Brazil | BRA |  |
| EMBRAPA | *Spodoptera eridania* (Stoll) | GBGL12632-13 | KF261169 | Brazil | BRA |  |
| EMBRAPA | *Spodoptera eridania* (Stoll) | GBGL12631-13 | KF261170 | Brazil | BRA |  |
| EMBRAPA | *Spodoptera eridania* (Stoll) | GBGL12630-13 | KF261171 | Brazil | BRA |  |
| CBGP | *Spodoptera evanida* Schaus | B155* | HQ177310 | French Guiana | GUF | Kergoat *et al*. 2012 |
| CBGP | *Spodoptera evanida* Schaus | B156* | HQ177311 | French Guiana | GUF | Kergoat *et al*. 2012 |
| MNHN | *Spodoptera evanida* Schaus | MNHN52** | KF854173 | French Guiana | GUF | this study |
| MNHN | *Spodoptera evanida* Schaus | MNHN53** | KF854174 | French Guiana | GUF | this study |
| MNHN | *Spodoptera evanida* Schaus | MNHN54** | KF854175 | French Guiana | GUF | this study |
| MNHN | *Spodoptera evanida* Schaus | MNHN55** | KF854176 | French Guiana | GUF | this study |
| MNHN | *Spodoptera evanida* Schaus | MNHN60** | KF854177 | French Guiana | GUF | this study |
| MNHN | *Spodoptera evanida* Schaus | MNHN61** | KF854178 | French Guiana | GUF | this study |
| MNHN | *Spodoptera evanida* Schaus | MNHN71** | KF854179 | Peru | PER | this study |
| MNHN | *Spodoptera evanida* Schaus | MNHN72** | KF854180 | French Guiana | GUF | this study |
| CBGP | *Spodoptera exempta* (Walker) | B162* | HQ177334 | Kenya | KEN | Kergoat *et al*. 2012 |
| CBGP | *Spodoptera exempta* (Walker) | B163* | HQ177335 | Kenya | KEN | Kergoat *et al*. 2012 |
| CBGP | *Spodoptera exempta* (Walker) | B24* | HQ177331 | Laboratory colony | N/A | Kergoat *et al*. 2012 |
| CBGP | *Spodoptera exempta* (Walker) | B25* | HQ177332 | Laboratory colony | N/A | Kergoat *et al*. 2012 |
| CBGP | *Spodoptera exempta* (Walker) | B93* | HQ177333 | Laboratory colony | N/A | Kergoat *et al*. 2012 |
| NIAES | *Spodoptera exempta* (Walker) | NIAES:LEPID:247 | AB735194 | Japan | JAP |  |
| NIAES | *Spodoptera exempta* (Walker) | NIAES:LEPID:248 | AB735195 | Japan | JAP |  |
| NIAES | *Spodoptera exempta* (Walker) | NIAES:LEPID:249 | AB735196 | Japan | JAP |  |
| NIAES | *Spodoptera exempta* (Walker) | NIAES:LEPID:251 | AB735197 | Japan | JAP |  |
| NIAES | *Spodoptera exempta* (Walker) | NIAES:LEPID:252 | AB735198 | Japan | JAP |  |
| NIAES | *Spodoptera exempta* (Walker) | NIAES:LEPID:253 | AB735199 | Japan | JAP |  |
| NIAES | *Spodoptera exempta* (Walker) | NIAES:LEPID:254 | AB735200 | Japan | JAP |  |
| NIAES | *Spodoptera exempta* (Walker) | NIAES:LEPID:255 | AB735201 | Japan | JAP |  |
| NIAES | *Spodoptera exempta* (Walker) | NIAES:LEPID:256 | AB735202 | Japan | JAP |  |
| NIAES | *Spodoptera exempta* (Walker) | NIAES:LEPID:257 | AB735203 | Japan | JAP |  |
| NIAES | *Spodoptera exempta* (Walker) | NIAES:LEPID:258 | AB735204 | Japan | JAP |  |
| NIAES | *Spodoptera exempta* (Walker) | NIAES:LEPID:260 | AB735205 | Japan | JAP |  |
| NIAES | *Spodoptera exempta* (Walker) | NIAES:LEPID:261 | AB735206 | Japan | JAP |  |
| NIAES | *Spodoptera exempta* (Walker) | NIAES:LEPID:262 | AB735207 | Japan | JAP |  |
| NIAES | *Spodoptera exempta* (Walker) | NIAES:LEPID:263 | AB735208 | Japan | JAP |  |
| NIAES | *Spodoptera exempta* (Walker) | NIAES:LEPID:264 | AB735209 | Japan | JAP |  |
| NIAES | *Spodoptera exempta* (Walker) | NIAES:LEPID:265 | AB735210 | Japan | JAP |  |
| NIAES | *Spodoptera exempta* (Walker) | NIAES:LEPID:266 | AB735211 | Japan | JAP |  |
| NIAES | *Spodoptera exempta* (Walker) | NIAES:LEPID:267 | AB735212 | Japan | JAP |  |
| NIAES | *Spodoptera exempta* (Walker) | NIAES:LEPID:268 | AB735213 | Japan | JAP |  |
| NIAES | *Spodoptera exempta* (Walker) | NIAES:LEPID:269 | AB735214 | Japan | JAP |  |
| NIAES | *Spodoptera exempta* (Walker) | NIAES:LEPID:270 | AB735215 | Japan | JAP |  |
| NIAES | *Spodoptera exempta* (Walker) | NIAES:LEPID:271 | AB735216 | Japan | JAP |  |
| NIAES | *Spodoptera exempta* (Walker) | NIAES:LEPID:272 | AB735217 | Japan | JAP |  |
| NIAES | *Spodoptera exempta* (Walker) | NIAES:LEPID:273 | AB735218 | Japan | JAP |  |
| NIAES | *Spodoptera exempta* (Walker) | NIAES:LEPID:275 | AB735219 | Japan | JAP |  |
| NIAES | *Spodoptera exempta* (Walker) | NIAES:LEPID:276 | AB735220 | Japan | JAP |  |
| NIAES | *Spodoptera exempta* (Walker) | NIAES:LEPID:143 | AB735221 | Japan | JAP |  |
| NIAES | *Spodoptera exempta* (Walker) | NIAES:LEPID:144 | AB735222 | Japan | JAP |  |
| NIAES | *Spodoptera exempta* (Walker) | NIAES:LEPID:145 | AB735223 | Japan | JAP |  |
| NIAES | *Spodoptera exempta* (Walker) | NIAES:LEPID:146 | AB735224 | Japan | JAP |  |
| NIAES | *Spodoptera exempta* (Walker) | NIAES:LEPID:147 | AB735225 | Japan | JAP |  |
| NIAES | *Spodoptera exempta* (Walker) | NIAES:LEPID:148 | AB735226 | Japan | JAP |  |
| NIAES | *Spodoptera exempta* (Walker) | NIAES:LEPID:149 | AB735227 | Japan | JAP |  |
| NIAES | *Spodoptera exempta* (Walker) | NIAES:LEPID:162 | AB735228 | Japan | JAP |  |
| NIAES | *Spodoptera exempta* (Walker) | NIAES:LEPID:163 | AB735229 | Japan | JAP |  |
| NIAES | *Spodoptera exempta* (Walker) | NIAES:LEPID:164 | AB735230 | Japan | JAP |  |
| NIAES | *Spodoptera exempta* (Walker) | NIAES:LEPID:165 | AB735231 | Japan | JAP |  |
| NIAES | *Spodoptera exempta* (Walker) | NIAES:LEPID:166 | AB735232 | Japan | JAP |  |
| NIAES | *Spodoptera exempta* (Walker) | NIAES:LEPID:167 | AB735233 | Japan | JAP |  |
| NIAES | *Spodoptera exempta* (Walker) | NIAES:LEPID:274 | AB735239 | Japan | JAP |  |
| UR | *Spodoptera exempta* (Walker) | MGABB511-10 | HM893111 | Gabon | GAB |  |
| LEC | *Spodoptera exempta* (Walker) | GBMIN17394-13 | JQ315120 | Tanzania | TZA | Graham *et al*. 2012 |
| LEC | *Spodoptera exempta* (Walker) | GBMIN17302-13 | JQ315122 | Tanzania | TZA | Graham *et al*. 2012 |
| LEC | *Spodoptera exempta* (Walker) | GBMIN17393-13 | JQ315125 | Tanzania | TZA | Graham *et al*. 2012 |
| LEC | *Spodoptera exempta* (Walker) | GBMIN17301-13 | JQ315126 | Tanzania | TZA | Graham *et al*. 2012 |
| LEC | *Spodoptera exempta* (Walker) | GBMIN17392-13 | JQ315127 | Tanzania | TZA | Graham *et al*. 2012 |
| LEC | *Spodoptera exempta* (Walker) | GBMIN17300-13 | JQ315128 | Tanzania | TZA | Graham *et al*. 2012 |
| LEC | *Spodoptera exempta* (Walker) | GBMIN17391-13 | JQ315129 | Tanzania | TZA | Graham *et al*. 2012 |
| LEC | *Spodoptera exempta* (Walker) | GBMIN17299-13 | JQ315130 | Tanzania | TZA | Graham *et al*. 2012 |
| LEC | *Spodoptera exempta* (Walker) | GBMIN17390-13 | JQ315131 | Tanzania | TZA | Graham *et al*. 2012 |
| LEC | *Spodoptera exempta* (Walker) | GBMIN17298-13 | JQ315136 | Tanzania | TZA | Graham *et al*. 2012 |
| SI | *Spodoptera exempta* (Walker) | LPNGM110-07 | JX970437 | Papua New Guinea | PNG |  |
| SI | *Spodoptera exempta* (Walker) | LPNGM108-07 | JX970438 | Papua New Guinea | PNG |  |
| SI | *Spodoptera exempta* (Walker) | LPNGM107-07 | JX970439 | Papua New Guinea | PNG |  |
| NIAES | *Spodoptera exigua* (Hübner) | NIAES:LEPID:199 | AB733673 | Japan | JAP |  |
| NIAES | *Spodoptera exigua* (Hübner) | NIAES:LEPID:200 | AB733674 | Japan | JAP |  |
| NIAES | *Spodoptera exigua* (Hübner) | NIAES:LEPID:201 | AB733675 | Japan | JAP |  |
| UM | *Spodoptera exigua* (Hübner) | GBGL4999-08 | EU779856 | Laboratory colony | N/A |  |
| FERA | *Spodoptera exigua* (Hübner) | GBMIN38597-13 | FN907973 | unknown | N/A |  |
| FERA | *Spodoptera exigua* (Hübner) | GBMIN38646-13 | FN907974 | unknown | N/A |  |
| FERA | *Spodoptera exigua* (Hübner) | GBMIN38596-13 | FN907975 | unknown | N/A |  |
| FERA | *Spodoptera exigua* (Hübner) | GBMIN38631-13 | FN908004 | Thailand | THA |  |
| FERA | *Spodoptera exigua* (Hübner) | GBMIN38621-13 | FN908024 | Spain | ESP |  |
| BSCZ | *Spodoptera exigua* (Hübner) | GWORO707-09 | JF415658 | Germany | DEU |  |
| TAU | *Spodoptera exigua* (Hübner) | GBMIN30171-13 | JQ064572 | India | IND |  |
| UM | *Spodoptera exigua* (Hübner) | LAUSM003-09 | KF522648 | Australia | AUS |  |
| UM | *Spodoptera exigua* (Hübner) | LAUSM012-09 | KF522649 | Australia | AUS |  |
| UM | *Spodoptera exigua* (Hübner) | LAUSM010-09 | KF522650 | Australia | AUS |  |
| UM | *Spodoptera exigua* (Hübner) | LAUSM011-09 | KF522651 | Australia | AUS |  |
| BIO | *Spodoptera exigua* (Hübner) | PHMO362-03 | GU094753 | Canada - Ontario | CAN |  |
| RCG | *Spodoptera exigua* (Hübner) | FBLMV381-09 | GU707393 | Germany | DEU |  |
| CBGP | *Spodoptera exigua* (Hübner) | B34* | HQ177337 | Kenya | KEN | Kergoat *et al*. 2012 |
| CBGP | *Spodoptera exigua* (Hübner) | B67* | HQ177338 | USA - California | USA | Kergoat *et al*. 2012 |
| CBGP | *Spodoptera exigua* (Hübner) | B68* | HQ177339 | Laboratory colony | N/A | Kergoat *et al*. 2012 |
| CBGP | *Spodoptera exigua* (Hübner) | B69* | HQ177340 | Laboratory colony | N/A | Kergoat *et al*. 2012 |
| CBGP | *Spodoptera exigua* (Hübner) | G2178* | HQ177343 | Egypt | EGY | Kergoat *et al*. 2012 |
| RCH | *Spodoptera exigua* (Hübner) | GWORZ535-10 | HM914242 | Germany | DEU |  |
| CBGP | *Spodoptera exigua* (Hübner) | G2182* | HQ177344 | Egypt | EGY | Kergoat *et al*. 2012 |
| CBGP | *Spodoptera exigua* (Hübner) | G2185* | HQ177345 | Egypt | EGY | Kergoat *et al*. 2012 |
| USDA | *Spodoptera exigua* (Hübner) | GBGL10115-12 | HM756077 | USA - Florida | USA | Nagoshi *et al*. 2011 |
| USDA | *Spodoptera exigua* (Hübner) | GBGL10116-12 | HM756078 | USA - Florida | USA | Nagoshi *et al*. 2011 |
| USDA | *Spodoptera exigua* (Hübner) | GBGL10117-12 | HM756079 | USA - Florida | USA | Nagoshi *et al*. 2011 |
| USDA | *Spodoptera exigua* (Hübner) | GBGL10118-12 | HM756080 | USA - Florida | USA | Nagoshi *et al*. 2011 |
| CBGP | *Spodoptera exigua* (Hübner) | LSU11* | HQ177341 | USA | USA | Kergoat *et al*. 2012 |
| CBGP | *Spodoptera exigua* (Hübner) | LSU15* | HQ177342 | USA- California | USA | Kergoat *et al*. 2012 |
| CBGP | *Spodoptera frugiperda* (J.E. Smith) | B90C* | HQ177351 | Peru | PER | Kergoat *et al*. 2012 |
| CBGP | *Spodoptera frugiperda* (J.E. Smith) | FRUB1** | KF854181 | Brazil | BRA | this study |
| CBGP | *Spodoptera frugiperda* (J.E. Smith) | FRUB11** | KF854182 | Brazil | BRA | this study |
| CBGP | *Spodoptera frugiperda* (J.E. Smith) | FRUB12** | KF854183 | Brazil | BRA | this study |
| CBGP | *Spodoptera frugiperda* (J.E. Smith) | FRUB13** | KF854184 | Brazil | BRA | this study |
| CBGP | *Spodoptera frugiperda* (J.E. Smith) | FRUB14** | KF854185 | Brazil | BRA | this study |
| CBGP | *Spodoptera frugiperda* (J.E. Smith) | FRUB15** | KF854186 | Brazil | BRA | this study |
| CBGP | *Spodoptera frugiperda* (J.E. Smith) | FRUB16** | KF854187 | Brazil | BRA | this study |
| CBGP | *Spodoptera frugiperda* (J.E. Smith) | FRUB17** | KF854188 | Brazil | BRA | this study |
| CBGP | *Spodoptera frugiperda* (J.E. Smith) | FRUB3** | KF854189 | Brazil | BRA | this study |
| CBGP | *Spodoptera frugiperda* (J.E. Smith) | FRUB4** | KF854190 | Brazil | BRA | this study |
| CBGP | *Spodoptera frugiperda* (J.E. Smith) | FRUB7** | KF854191 | Brazil | BRA | this study |
| CBGP | *Spodoptera frugiperda* (J.E. Smith) | FRUB8** | KF854192 | Brazil | BRA | this study |
| CBGP | *Spodoptera frugiperda* (J.E. Smith) | G1 135** | KF854193 | Guadeloupe | GLP | this study |
| CBGP | *Spodoptera frugiperda* (J.E. Smith) | G4 153** | KF854194 | Guadeloupe | GLP | this study |
| USDA | *Spodoptera frugiperda* (J.E. Smith) | GBGL10069-12 | HM136586 | USA - Florida | USA | Nagoshi *et al.* 2011 |
| USDA | *Spodoptera frugiperda* (J.E. Smith) | GBGL10070-12 | HM136587 | USA - Florida | USA | Nagoshi *et al.* 2011 |
| USDA | *Spodoptera frugiperda* (J.E. Smith) | GBGL10071-12 | HM136588 | USA - Florida | USA | Nagoshi *et al.* 2011 |
| USDA | *Spodoptera frugiperda* (J.E. Smith) | GBGL10072-12 | HM136589 | USA - Florida | USA | Nagoshi *et al.* 2011 |
| USDA | *Spodoptera frugiperda* (J.E. Smith) | GBGL10073-12 | HM136590 | USA - Florida | USA | Nagoshi *et al.* 2011 |
| USDA | *Spodoptera frugiperda* (J.E. Smith) | GBGL10074-12 | HM136591 | USA - Florida | USA | Nagoshi *et al.* 2011 |
| USDA | *Spodoptera frugiperda* (J.E. Smith) | GBGL10075-12 | HM136592 | USA - Florida | USA | Nagoshi *et al.* 2011 |
| CBGP | *Spodoptera frugiperda* (J.E. Smith) | LSU21* | HQ177353 | Guadeloupe | GLP | Kergoat *et al*. 2012 |
| MNHN | *Spodoptera frugiperda* (J.E. Smith) | MNHN12** | KF854195 | Paraguay | PRY | this study |
| MNHN | *Spodoptera frugiperda* (J.E. Smith) | MNHN14** | KF854196 | Dominican Repubic | DOM | this study |
| MNHN | *Spodoptera frugiperda* (J.E. Smith) | MNHN16** | KF854197 | Dominican Repubic | DOM | this study |
| MNHN | *Spodoptera frugiperda* (J.E. Smith) | MNHN56** | KF854198 | Cuba | CUB | this study |
| MNHN | *Spodoptera frugiperda* (J.E. Smith) | MNHN81** | KF854199 | Peru | PER | this study |
| CBGP | *Spodoptera frugiperda* (J.E. Smith) | 311 MF67** | KF854200 | French Guiana | GUF | this study |
| CBGP | *Spodoptera frugiperda* (J.E. Smith) | 337 MR7** | KF854201 | French Guiana | GUF | this study |
| CBGP | *Spodoptera frugiperda* (J.E. Smith) | B10R* | HQ177348 | French Guiana | GUF | Kergoat *et al*. 2012 |
| CBGP | *Spodoptera frugiperda* (J.E. Smith) | B12R* | HQ177349 | French Guiana | GUF | Kergoat *et al*. 2012 |
| CBGP | *Spodoptera frugiperda* (J.E. Smith) | B7R* | HQ177346 | French Guiana | GUF | Kergoat *et al*. 2012 |
| CBGP | *Spodoptera frugiperda* (J.E. Smith) | CE1** | KF854202 | French Guiana | GUF | this study |
| CBGP | *Spodoptera frugiperda* (J.E. Smith) | F1 329** | KF854203 | French Guiana | GUF | this study |
| CBGP | *Spodoptera frugiperda* (J.E. Smith) | F1 330** | KF854204 | French Guiana | GUF | this study |
| CBGP | *Spodoptera frugiperda* (J.E. Smith) | F4 322** | KF854205 | French Guiana | GUF | this study |
| CBGP | *Spodoptera frugiperda* (J.E. Smith) | FRUB10** | KF854206 | Brazil | BRA | this study |
| CBGP | *Spodoptera frugiperda* (J.E. Smith) | FRUB9** | KF854207 | Brazil | BRA | this study |
| CBGP | *Spodoptera frugiperda* (J.E. Smith) | G9 305** | KF854208 | French Guiana | GUF | this study |
| CBGP | *Spodoptera frugiperda* (J.E. Smith) | G9 308** | KF854209 | French Guiana | GUF | this study |
| CBGP | *Spodoptera frugiperda* (J.E. Smith) | GK429** | KF854212 | Brazil | BRA | this study |
| USDA | *Spodoptera frugiperda* (J.E. Smith) | GBGL10076-12 | HM136593 | USA - Florida | USA | Nagoshi *et al.* 2011 |
| USDA | *Spodoptera frugiperda* (J.E. Smith) | GBGL10077-12 | HM136594 | USA - Florida | USA | Nagoshi *et al.* 2011 |
| USDA | *Spodoptera frugiperda* (J.E. Smith) | GBGL10078-12 | HM136595 | USA - Florida | USA | Nagoshi *et al.* 2011 |
| USDA | *Spodoptera frugiperda* (J.E. Smith) | GBGL10079-12 | HM136596 | USA - Florida | USA | Nagoshi *et al.* 2011 |
| USDA | *Spodoptera frugiperda* (J.E. Smith) | GBGL10080-12 | HM136597 | USA - Florida | USA | Nagoshi *et al.* 2011 |
| USDA | *Spodoptera frugiperda* (J.E. Smith) | GBGL10081-12 | HM136598 | USA - Florida | USA | Nagoshi *et al.* 2011 |
| CBGP | *Spodoptera frugiperda* (J.E. Smith) | LSU22R* | HQ177350 | Guadeloupe | GLP | Kergoat *et al*. 2012 |
| CBGP | *Spodoptera frugiperda* (J.E. Smith) | MFG 50** | KF854213 | French Guiana | GUF | this study |
| CBGP | *Spodoptera frugiperda* (J.E. Smith) | MFG 56** | KF854214 | French Guiana | GUF | this study |
| MNHN | *Spodoptera frugiperda* (J.E. Smith) | MNHN10** | KF854215 | Dominican Repubic | DOM | this study |
| MNHN | *Spodoptera frugiperda* (J.E. Smith) | MNHN11** | KF854216 | Venezuela | VEN | this study |
| MNHN | *Spodoptera frugiperda* (J.E. Smith) | MNHN13** | KF854217 | Mexico | MEX | this study |
| MNHN | *Spodoptera frugiperda* (J.E. Smith) | MNHN15** | KF854218 | Guatemala | GTM | this study |
| MNHN | *Spodoptera frugiperda* (J.E. Smith) | MNHN9** | KF854219 | Dominican Repubic | DOM | this study |
| UM | *Spodoptera frugiperda* (J.E. Smith) | GBGL5879-09 | EU768963 | unknown | N/A |  |
| BIO | *Spodoptera frugiperda* (J.E. Smith) | XAH548-05 | GU090723 | Canada - Ontario | CAN | Hebert *et al*. 2010 |
| BIO | *Spodoptera frugiperda* (J.E. Smith) | XAH550-05 | GU090724 | Canada - Ontario | CAN | Hebert *et al*. 2010 |
| BIO | *Spodoptera frugiperda* (J.E. Smith) | PHMO299-03 | GU094754 | Canada - New Brunswick | CAN | Hebert *et al*. 2010 |
| BIO | *Spodoptera frugiperda* (J.E. Smith) | PHMO305-03 | GU094755 | Canada - New Brunswick | CAN | Hebert *et al*. 2010 |
| BIO | *Spodoptera frugiperda* (J.E. Smith) | PHMO358-03 | GU094756 | Canada - New Brunswick | CAN | Hebert *et al*. 2010 |
| BIO | *Spodoptera frugiperda* (J.E. Smith) | MNBB563-05 | GU095403 | Canada - New Brunswick | CAN | Hebert *et al*. 2010 |
| PSCAS | *Spodoptera frugiperda* (J.E. Smith) | MHAUB890-05 | GU159426 | Costa Rica | CRI |  |
| PSCAS | *Spodoptera frugiperda* (J.E. Smith) | MHAUB893-05 | GU159427 | Costa Rica | CRI |  |
| PSCAS | *Spodoptera frugiperda* (J.E. Smith) | MHAUB892-05 | GU159428 | Costa Rica | CRI |  |
| PSCAS | *Spodoptera frugiperda* (J.E. Smith) | MHAUB886-05 | GU159429 | Costa Rica | CRI |  |
| PSCAS | *Spodoptera frugiperda* (J.E. Smith) | MHAUB888-05 | GU159430 | Costa Rica | CRI |  |
| PSCAS | *Spodoptera frugiperda* (J.E. Smith) | MHAUB889-05 | GU159431 | Costa Rica | CRI |  |
| PSCAS | *Spodoptera frugiperda* (J.E. Smith) | MHAUB887-05 | GU159432 | Costa Rica | CRI |  |
| PSCAS | *Spodoptera frugiperda* (J.E. Smith) | MHAUB885-05 | GU159433 | Costa Rica | CRI |  |
| PSCAS | *Spodoptera frugiperda* (J.E. Smith) | MHAUB891-05 | GU159434 | Costa Rica | CRI |  |
| PSCAS | *Spodoptera frugiperda* (J.E. Smith) | MHAUB894-05 | GU159435 | Costa Rica | CRI |  |
| PSCAS | *Spodoptera frugiperda* (J.E. Smith) | MHAUF155-06 | GU163698 | Costa Rica | CRI |  |
| BIO | *Spodoptera frugiperda* (J.E. Smith) | XAD236-04 | GU439147 | Canada - Ontario | CAN |  |
| BIO | *Spodoptera frugiperda* (J.E. Smith) | XAD239-04 | GU439148 | Canada - Ontario | CAN |  |
| BIO | *Spodoptera frugiperda* (J.E. Smith) | XAD265-04 | GU439149 | Canada - Ontario | CAN |  |
| BIO | *Spodoptera frugiperda* (J.E. Smith) | XAD490-04 | GU439150 | Canada - Ontario | CAN |  |
| BIO | *Spodoptera frugiperda* (J.E. Smith) | XAD509-04 | GU439151 | Canada - Ontario | CAN |  |
| RCJH | *Spodoptera frugiperda* (J.E. Smith) | MHMYE1162-09 | GU653637 | Costa Rica | CRI |  |
| BIO | *Spodoptera frugiperda* (J.E. Smith) | MJMSL002-10 | HQ964352 | USA - Massachusetts | USA |  |
| BIO | *Spodoptera frugiperda* (J.E. Smith) | MJMSL151-10 | HQ964487 | USA - Massachusetts | USA |  |
| UFP | *Spodoptera frugiperda* (J.E. Smith) | LEMMZ127-10 | JF854740 | Brazil | BRA |  |
| UFP | *Spodoptera frugiperda* (J.E. Smith) | LEMMZ128-10 | JF854741 | Brazil | BRA |  |
| UFP | *Spodoptera frugiperda* (J.E. Smith) | LEMMZ130-10 | JF854743 | Brazil | BRA |  |
| UFP | *Spodoptera frugiperda* (J.E. Smith) | LEMMZ131-10 | JF854744 | Brazil | BRA |  |
| UFP | *Spodoptera frugiperda* (J.E. Smith) | LEMMZ132-10 | JF854745 | Brazil | BRA |  |
| UFP | *Spodoptera frugiperda* (J.E. Smith) | LEMMZ133-10 | JF854746 | Brazil | BRA |  |
| UFP | *Spodoptera frugiperda* (J.E. Smith) | LEMMZ134-10 | JF854747 | Brazil | BRA |  |
| ACG | *Spodoptera frugiperda* (J.E. Smith) | BLPBB657-07 | JN807246 | Costa Rica | CRI |  |
| RCJH | *Spodoptera frugiperda* (J.E. Smith) | IBOLG016-08 | JQ547900 | Costa Rica | CRI |  |
| ACG | *Spodoptera frugiperda* (J.E. Smith) | BLPDE131-09 | JQ554012 | Costa Rica | CRI |  |
| ACG | *Spodoptera frugiperda* (J.E. Smith) | BLPCC401-08 | JQ559528 | Costa Rica | CRI |  |
| ACG | *Spodoptera frugiperda* (J.E. Smith) | BLPAG434-07 | JQ571459 | Costa Rica | CRI |  |
| ACG | *Spodoptera frugiperda* (J.E. Smith) | BLPBA709-07 | JQ572603 | Costa Rica | CRI |  |
| ACG | *Spodoptera frugiperda* (J.E. Smith) | BLPAB120-06 | JQ577923 | Costa Rica | CRI |  |
| CTAG | *Spodoptera frugiperda* (J.E. Smith) | GBGL3727-06 | U72974 | unknown | N/A |  |
| CTAG | *Spodoptera frugiperda* (J.E. Smith) | GBGL3728-06 | U72975 | unknown | N/A |  |
| CTAG | *Spodoptera frugiperda* (J.E. Smith) | GBGL3729-06 | U72976 | unknown | N/A |  |
| CTAG | *Spodoptera frugiperda* (J.E. Smith) | GBGL3730-06 | U72977 | unknown | N/A |  |
| CTAG | *Spodoptera frugiperda* (J.E. Smith) | GBGL3731-06 | U72978 | unknown | N/A |  |
| PSCAS | *Spodoptera latifascia* (Walker) | MHAUB922-05 | GU159436 | Costa Rica | CRI |  |
| PSCAS | *Spodoptera latifascia* (Walker) | MHAUB924-05 | GU159437 | Costa Rica | CRI |  |
| PSCAS | *Spodoptera latifascia* (Walker) | MHAUB925-05 | GU159438 | Costa Rica | CRI |  |
| PSCAS | *Spodoptera latifascia* (Walker) | MHAUB918-05 | GU159439 | Costa Rica | CRI |  |
| PSCAS | *Spodoptera latifascia* (Walker) | MHAUB921-05 | GU159440 | Costa Rica | CRI |  |
| PSCAS | *Spodoptera latifascia* (Walker) | MHAUB919-05 | GU159441 | Costa Rica | CRI |  |
| PSCAS | *Spodoptera latifascia* (Walker) | MHAUB920-05 | GU159442 | Costa Rica | CRI |  |
| PSCAS | *Spodoptera latifascia* (Walker) | MHAUB916-05 | GU159443 | Costa Rica | CRI |  |
| PSCAS | *Spodoptera latifascia* (Walker) | MHAUB917-05 | GU159444 | Costa Rica | CRI |  |
| PSCAS | *Spodoptera latifascia* (Walker) | MHAUB923-05 | GU159445 | Costa Rica | CRI |  |
| PSCAS | *Spodoptera latifascia* (Walker) | MHAUC211-06 | GU337022 | Costa Rica | CRI |  |
| RCJH | *Spodoptera latifascia* (Walker) | MHMYC2258-09 | GU651179 | Costa Rica | CRI |  |
| RCJH | *Spodoptera latifascia* (Walker) | MHMXU488-08 | JF751689 | Costa Rica | CRI |  |
| ACG | *Spodoptera latifascia* (Walker) | BLPDU281-11 | JF844085 | Costa Rica | CRI |  |
| ACG | *Spodoptera latifascia* (Walker) | BLPDU282-11 | JF844086 | Costa Rica | CRI |  |
| ACG | *Spodoptera latifascia* (Walker) | BLPDU283-11 | JF844087 | Costa Rica | CRI |  |
| ACG | *Spodoptera latifascia* (Walker) | BLPDU284-11 | JF844088 | Costa Rica | CRI |  |
| ACG | *Spodoptera latifascia* (Walker) | BLPDU884-11 | JF844655 | Costa Rica | CRI |  |
| ACG | *Spodoptera latifascia* (Walker) | BLPDV1050-11 | JF844917 | Costa Rica | CRI |  |
| ACG | *Spodoptera latifascia* (Walker) | BLPDV1051-11 | JF844918 | Costa Rica | CRI |  |
| ACG | *Spodoptera latifascia* (Walker) | BLPDV1056-11 | JF844921 | Costa Rica | CRI |  |
| ACG | *Spodoptera latifascia* (Walker) | BLPDY798-11 | JN261856 | Costa Rica | CRI |  |
| ACG | *Spodoptera latifascia* (Walker) | BLPDB355-09 | JQ551692 | Costa Rica | CRI |  |
| ACG | *Spodoptera latifascia* (Walker) | BLPDE113-09 | JQ553994 | Costa Rica | CRI |  |
| ACG | *Spodoptera latifascia* (Walker) | BLPDE114-09 | JQ553995 | Costa Rica | CRI |  |
| ACG | *Spodoptera latifascia* (Walker) | BLPDE115-09 | JQ553996 | Costa Rica | CRI |  |
| ACG | *Spodoptera latifascia* (Walker) | BLPCI616-08 | JQ554807 | Costa Rica | CRI |  |
| ACG | *Spodoptera latifascia* (Walker) | BLPCI617-08 | JQ554808 | Costa Rica | CRI |  |
| ACG | *Spodoptera latifascia* (Walker) | BLPCJ553-08 | JQ555655 | Costa Rica | CRI |  |
| ACG | *Spodoptera latifascia* (Walker) | BLPCJ554-08 | JQ555656 | Costa Rica | CRI |  |
| ACG | *Spodoptera latifascia* (Walker) | BLPCJ555-08 | JQ556283 | Costa Rica | CRI |  |
| RCJH | *Spodoptera latifascia* (Walker) | BLPCK290-08 | JQ556284 | Costa Rica | CRI |  |
| RCJH | *Spodoptera latifascia* (Walker) | BLPCL008-08 | JQ556958 | Costa Rica | CRI |  |
| ACG | *Spodoptera latifascia* (Walker) | BLPCD474-08 | JQ560336 | Costa Rica | CRI |  |
| ACG | *Spodoptera latifascia* (Walker) | BLPCD477-08 | JQ560339 | Costa Rica | CRI |  |
| ACG | *Spodoptera latifascia* (Walker) | BLPCD479-08 | JQ560341 | Costa Rica | CRI |  |
| ACG | *Spodoptera latifascia* (Walker) | BLPCD480-08 | JQ560342 | Costa Rica | CRI |  |
| ACG | *Spodoptera latifascia* (Walker) | BLPCH656-08 | JQ563957 | Costa Rica | CRI |  |
| ACG | *Spodoptera latifascia* (Walker) | BLPCI137-08 | JQ564356 | Costa Rica | CRI |  |
| ACG | *Spodoptera latifascia* (Walker) | BLPBF915-07 | JQ566087 | Costa Rica | CRI |  |
| ACG | *Spodoptera latifascia* (Walker) | BLPBH663-07 | JQ567480 | Costa Rica | CRI |  |
| ACG | *Spodoptera latifascia* (Walker) | BLPCA159-08 | JQ567765 | Costa Rica | CRI |  |
| ACG | *Spodoptera latifascia* (Walker) | BLPCA207-08 | JQ567807 | Costa Rica | CRI |  |
| ACG | *Spodoptera latifascia* (Walker) | BLPCA208-08 | JQ567808 | Costa Rica | CRI |  |
| ACG | *Spodoptera latifascia* (Walker) | BLPCA519-08 | JQ568077 | Costa Rica | CRI |  |
| ACG | *Spodoptera latifascia* (Walker) | BLPCA520-08 | JQ568078 | Costa Rica | CRI |  |
| ACG | *Spodoptera latifascia* (Walker) | BLPCA521-08 | JQ568079 | Costa Rica | CRI |  |
| ACG | *Spodoptera latifascia* (Walker) | BLPCA522-08 | JQ568080 | Costa Rica | CRI |  |
| ACG | *Spodoptera latifascia* (Walker) | BLPCA523-08 | JQ568081 | Costa Rica | CRI |  |
| ACG | *Spodoptera latifascia* (Walker) | BLPCA525-08 | JQ568082 | Costa Rica | CRI |  |
| ACG | *Spodoptera latifascia* (Walker) | BLPAF457-07 | JQ570811 | Costa Rica | CRI |  |
| ACG | *Spodoptera latifascia* (Walker) | BLPAG029-07 | JQ571202 | Costa Rica | CRI |  |
| ACG | *Spodoptera latifascia* (Walker) | BLPAG031-07 | JQ571204 | Costa Rica | CRI |  |
| ACG | *Spodoptera latifascia* (Walker) | BLPBA005-07 | JQ572097 | Costa Rica | CRI |  |
| ACG | *Spodoptera latifascia* (Walker) | BLPAA382-06 | JQ577376 | Costa Rica | CRI |  |
| ACG | *Spodoptera latifascia* (Walker) | BLPAA386-06 | JQ577377 | Costa Rica | CRI |  |
| ACG | *Spodoptera latifascia* (Walker) | BLPAA394-06 | JQ577384 | Costa Rica | CRI |  |
| ACG | *Spodoptera latifascia* (Walker) | BLPAA463-06 | JQ577447 | Costa Rica | CRI |  |
| ACG | *Spodoptera latifascia* (Walker) | BLPAA707-06 | JQ577649 | Costa Rica | CRI |  |
| ACG | *Spodoptera latifascia* (Walker) | BLPAA730-06 | JQ577665 | Costa Rica | CRI |  |
| ACG | *Spodoptera latifascia* (Walker) | BLPAA922-06 | JQ577817 | Costa Rica | CRI |  |
| ACG | *Spodoptera latifascia* (Walker) | BLPAB893-06 | JQ578525 | Costa Rica | CRI |  |
| ACG | *Spodoptera latifascia* (Walker) | BLPAC244-06 | JQ578737 | Costa Rica | CRI |  |
| ACG | *Spodoptera latifascia* (Walker) | BLPAC247-06 | JQ578740 | Costa Rica | CRI |  |
| ACG | *Spodoptera latifascia* (Walker) | BLPAC723-06 | JQ579064 | Costa Rica | CRI |  |
| ACG | *Spodoptera latifascia* (Walker) | BLPAC725-06 | JQ579066 | Costa Rica | CRI |  |
| ACG | *Spodoptera latifascia* (Walker) | BLPAD014-06 | JQ579185 | Costa Rica | CRI |  |
| PSCAS | *Spodoptera latifascia* (Walker) | MHMXA728-06 | JQ602909 | Costa Rica | CRI |  |
| PSCAS | *Spodoptera latifascia* (Walker) | MHMXA734-06 | JQ602914 | Costa Rica | CRI |  |
| PSCAS | *Spodoptera latifascia* (Walker) | MHMXA735-06 | JQ602916 | Costa Rica | CRI |  |
| PSCAS | *Spodoptera latifascia* (Walker) | MHAUG697-07 | JQ604827 | Costa Rica | CRI |  |
| MNHN | *Spodoptera latifascia* (Walker) | MNHN46** | KF854220 | Mexico | MEX | this study |
| MNHN | *Spodoptera latifascia* (Walker) | MNHN47** | KF854221 | Guatemala | GTM | this study |
| MNHN | *Spodoptera latifascia* (Walker) | MNHN48** | KF854222 | Costa Rica | CRI | this study |
| MNHN | *Spodoptera latifascia* (Walker) | MNHN51** | KF854223 | Dominican Republic | DOM | this study |
| CBGP | *Spodoptera latifascia* (Walker) | B123* | HQ177361 | USA - Georgia | USA | Kergoat *et al.* 2012 |
| CBGP | *Spodoptera latifascia* (Walker) | B18* | HQ177355 | Guadeloupe | GLP | Kergoat *et al.* 2012 |
| CBGP | *Spodoptera latifascia* (Walker) | B29* | HQ177356 | Guadeloupe | GLP | Kergoat *et al.* 2012 |
| CBGP | *Spodoptera latifascia* (Walker) | B3* | HQ177354 | USA - Louisiana | USA | Kergoat *et al.* 2012 |
| CBGP | *Spodoptera latifascia* (Walker) | B31* | HQ177357 | Barbados | BRB | Kergoat *et al.* 2012 |
| CBGP | *Spodoptera latifascia* (Walker) | B33* | HQ177358 | USA - Louisiana | USA | Kergoat *et al.* 2012 |
| CBGP | *Spodoptera latifascia* (Walker) | B36* | HQ177359 | Barbados | BRB | Kergoat *et al.* 2012 |
| CBGP | *Spodoptera latifascia* (Walker) | B39* | HQ177360 | USA - Louisiana | USA | Kergoat *et al.* 2012 |
| CBGP | *Spodoptera latifascia* (Walker) | LSU17* | HQ177363 | USA | USA | Kergoat *et al.* 2012 |
| CBGP | *Spodoptera latifascia* (Walker) | LSU6* | HQ177362 | Guadeloupe | GLP | Kergoat *et al.* 2012 |
| FERA | *Spodoptera littoralis* (Boisduval) | GBMIN38593-13 | FN907981 | unknown | N/A |  |
| FERA | *Spodoptera littoralis* (Boisduval) | GBMIN38642-13 | FN907982 | unknown | N/A |  |
| FERA | *Spodoptera littoralis* (Boisduval) | GBMIN38592-13 | FN907983 | unknown | N/A |  |
| FERA | *Spodoptera littoralis* (Boisduval) | GBMIN38641-13 | FN907984 | unknown | N/A |  |
| FERA | *Spodoptera littoralis* (Boisduval) | GBMIN38587-13 | FN907993 | unknown | N/A |  |
| FERA | *Spodoptera littoralis* (Boisduval) | GBMIN38580-13 | FN908007 | Viet Nam | VNM |  |
| FERA | *Spodoptera littoralis* (Boisduval) | GBMIN38629-13 | FN908008 | Ghana | GHA |  |
| FERA | *Spodoptera littoralis* (Boisduval) | GBMIN38579-13 | FN908009 | unknown | N/A |  |
| FERA | *Spodoptera littoralis* (Boisduval) | GBMIN38628-13 | FN908010 | Nigeria | NGA |  |
| FERA | *Spodoptera littoralis* (Boisduval) | GBMIN38627-13 | FN908012 | Israel | ISR |  |
| FERA | *Spodoptera littoralis* (Boisduval) | GBMIN38574-13 | FN908019 | Israel | ISR |  |
| CBGP | *Spodoptera littoralis* (Boisduval) | ADN0* | HQ177371 | Egypt | EGY | Kergoat *et al.* 2012 |
| CBGP | *Spodoptera littoralis* (Boisduval) | B14* | HQ177364 | Laboratory colony | N/A | Kergoat *et al.* 2012 |
| CBGP | *Spodoptera littoralis* (Boisduval) | B164* | HQ177367 | DRC | COD | Kergoat *et al.* 2012 |
| CBGP | *Spodoptera littoralis* (Boisduval) | B165* | HQ177368 | DRC | COD | Kergoat *et al.* 2012 |
| CBGP | *Spodoptera littoralis* (Boisduval) | B98* | HQ177365 | Egypt | EGY | Kergoat *et al.* 2012 |
| CBGP | *Spodoptera littoralis* (Boisduval) | B99* | HQ177366 | Egypt | EGY | Kergoat *et al.* 2012 |
| CBGP | *Spodoptera littoralis* (Boisduval) | GK118* | HQ177372 | Mali | MLI | Kergoat *et al.* 2012 |
| CBGP | *Spodoptera littoralis* (Boisduval) | GK119* | HQ177373 | Mali | MLI | Kergoat *et al.* 2012 |
| CBGP | *Spodoptera littoralis* (Boisduval) | GK120* | HQ177374 | Mali | MLI | Kergoat *et al.* 2012 |
| USDA | *Spodoptera littoralis* (Boisduval) | HM756074 | HM756074 | Portugal | PRT | Nagoshi *et al.* 2011 |
| CBGP | *Spodoptera littoralis* (Boisduval) | LSU35* | HQ177370 | Laboratory colony | N/A | Kergoat *et al.* 2012 |
| CBGP | *Spodoptera littoralis* (Boisduval) | LSU7* | HQ177369 | Laboratory colony | N/A | Kergoat *et al.* 2012 |
| NIAES | *Spodoptera litura* (Fabricius) | NIAES:LEPID:179 | AB733671 | Japan | JAP |  |
| NIAES | *Spodoptera litura* (Fabricius) | NIAES:LEPID:180 | AB733672 | Japan | JAP |  |
| CBGP | *Spodoptera litura* (Fabricius) | B113* | HQ177376 | Australia | AUS | Kergoat *et al.* 2012 |
| CBGP | *Spodoptera litura* (Fabricius) | B114* | HQ177377 | Australia | AUS | Kergoat *et al.* 2012 |
| CBGP | *Spodoptera litura* (Fabricius) | B15* | HQ177375 | Indonesia | IDN | Kergoat *et al.* 2012 |
| CBGP | *Spodoptera litura* (Fabricius) | B153* | HQ177378 | Indonesia | IDN | Kergoat *et al.* 2012 |
| CBGP | *Spodoptera litura* (Fabricius) | B154* | HQ177379 | Philippines | PHL | Kergoat *et al.* 2012 |
| CBGP | *Spodoptera litura* (Fabricius) | B160* | HQ177380 | Indonesia | IDN | Kergoat *et al.* 2012 |
| CBGP | *Spodoptera litura* (Fabricius) | GK425** | KF854224 | Indonesia | IDN | this study |
| CBGP | *Spodoptera litura* (Fabricius) | GK426** | KF854225 | Indonesia | IDN | this study |
| PAU | *Spodoptera litura* (Fabricius) | LEPIN003-12 | LEPIN003-12 | India | IND |  |
| USDA | *Spodoptera litura* (Fabricius) | GBGL10128-12 | HM756090 | Taiwan | TWN | Nagoshi *et al.* 2011 |
| USDA | *Spodoptera litura* (Fabricius) | GBGL10129-12 | HM756091 | Taiwan | TWN | Nagoshi *et al.* 2011 |
| USDA | *Spodoptera litura* (Fabricius) | GBGL10130-12 | HM756092 | Taiwan | TWN | Nagoshi *et al.* 2011 |
| USDA | *Spodoptera litura* (Fabricius) | GBGL10131-12 | HM756093 | Taiwan | CHN | Nagoshi *et al.* 2011 |
| CBGP | *Spodoptera litura* (Fabricius) | JSTR00021 0103** | KF854226 | China | CHN | this study |
| CBGP | *Spodoptera litura* (Fabricius) | JSTR00026 0201** | KF854227 | China | CHN | this study |
| CBGP | *Spodoptera litura* (Fabricius) | JSTR00026 0202** | KF854228 | China | CHN | this study |
| FERA | *Spodoptera litura* (Fabricius) | GBMIN38601-13 | FN907965 | Thailand | THA |  |
| FERA | *Spodoptera litura* (Fabricius) | GBMIN38650-13 | FN907966 | Bangladesh | BGD |  |
| FERA | *Spodoptera litura* (Fabricius) | GBMIN38600-13 | FN907967 | Bangladesh | BGD |  |
| FERA | *Spodoptera litura* (Fabricius) | GBMIN38649-13 | FN907968 | Bangladesh | BGD |  |
| FERA | *Spodoptera litura* (Fabricius) | GBMIN38599-13 | FN907969 | Bangladesh | BGD |  |
| FERA | *Spodoptera litura* (Fabricius) | GBMIN38636-13 | FN907994 | unknown | N/A |  |
| FERA | *Spodoptera litura* (Fabricius) | GBMIN38623-13 | FN908020 | Bangladesh | BGD |  |
| FERA | *Spodoptera litura* (Fabricius) | GBMIN38573-13 | FN908021 | Pakistan | PAK |  |
| FERA | *Spodoptera litura* (Fabricius) | GBMIN38622-13 | FN908022 | India | IND |  |
| FERA | *Spodoptera litura* (Fabricius) | GBMIN38571-13 | FN908025 | India | IND |  |
| ANIC | *Spodoptera litura* (Fabricius) | ANICK311-10 | HQ950413 | Australia | AUS |  |
| GSA | *Spodoptera litura* (Fabricius) | GBMIN22884-13 | JN087373 | South Korea | KOR |  |
| TAU | *Spodoptera litura* (Fabricius) | GBMIN30175-13 | JQ064564 | India | IND |  |
| TAU | *Spodoptera litura* (Fabricius) | GBMIN30170-13 | JQ064565 | India | IND |  |
| TAU | *Spodoptera litura* (Fabricius) | GBMIN30174-13 | JQ064566 | India | IND |  |
| TAU | *Spodoptera litura* (Fabricius) | GBMIN30169-13 | JQ064567 | India | IND |  |
| TAU | *Spodoptera litura* (Fabricius) | GBMIN30173-13 | JQ064568 | India | IND |  |
| TAU | *Spodoptera litura* (Fabricius) | GBMIN30168-13 | JQ064569 | India | IND |  |
| TAU | *Spodoptera litura* (Fabricius) | GBMIN30172-13 | JQ064570 | India | IND |  |
| TAU | *Spodoptera litura* (Fabricius) | GBMIN30167-13 | JQ064571 | India | IND |  |
| ZIQ | *Spodoptera litura* (Fabricius) | GBMIN22009-13 | JX156331 | China | CHN |  |
| NIMBB | *Spodoptera litura* (Fabricius) | GBGL12681-13 | KF022223 | Philippines | PHL |  |
| PAU | *Spodoptera litura* (Fabricius) | LEPIN014-13 | LEPIN014.13 | India | IND |  |
| PAU | *Spodoptera litura* (Fabricius) | LEPIN015-13 | LEPIN015.13 | India | IND |  |
| PAU | *Spodoptera litura* (Fabricius) | LEPIN044-15 | LEPIN044.13 | India | IND |  |
| CBGP | *Spodoptera marima* (Schaus) | B145* | HQ177381 | French Guiana | GUF | Kergoat *et al.* 2012 |
| MNHN | *Spodoptera marima* (Schaus) | MNHN39** | KF854229 | Venezuela | VEN | this study |
| MNHN | *Spodoptera marima* (Schaus) | MNHN40** | KF854230 | Venezuela | VEN | this study |
| MNHN | *Spodoptera marima* (Schaus) | MNHN58** | KF854231 | French Guiana | GUF | this study |
| NIAES | *Spodoptera mauritia* (Boisduval) | NIAES:LEPID:181 | AB733407 | Japan | JAP |  |
| NIAES | *Spodoptera mauritia* (Boisduval) | NIAES:LEPID:182 | AB733408 | Japan | JAP |  |
| NIAES | *Spodoptera mauritia* (Boisduval) | NIAES:LEPID:183 | AB733409 | Japan | JAP |  |
| ANIC | *Spodoptera mauritia* (Boisduval) | ANICK415-10 | KF389305 | Australia | AUS |  |
| CBGP | *Spodoptera mauritia* (Boisduval) | B152* | HQ177386 | Papua New Guinea | PNG | Kergoat *et al.* 2012 |
| CBGP | *Spodoptera mauritia* (Boisduval) | B65* | HQ177382 | Reunion | REU | Kergoat *et al.* 2012 |
| CBGP | *Spodoptera mauritia* (Boisduval) | B66* | HQ177383 | Reunion | REU | Kergoat *et al.* 2012 |
| CBGP | *Spodoptera mauritia* (Boisduval) | B94* | HQ177384 | Reunion | REU | Kergoat *et al.* 2012 |
| CBGP | *Spodoptera ochrea* (Hampson) | B110* | HQ177390 | Peru | PER | Kergoat *et al.* 2012 |
| CBGP | *Spodoptera ochrea* (Hampson) | B84* | HQ177387 | Peru | PER | Kergoat *et al.* 2012 |
| CBGP | *Spodoptera ochrea* (Hampson) | B85* | HQ177388 | Peru | PER | Kergoat *et al.* 2012 |
| CBGP | *Spodoptera ochrea* (Hampson) | B86* | HQ177389 | Peru | PER | Kergoat *et al.* 2012 |
| MNHN | *Spodoptera ochrea* (Hampson) | MNHN75** | KF854232 | Peru | PER | this study |
| CBGP | *Spodoptera ornithogalli* (Guenée) | B106* | HQ177394 | USA | USA | Kergoat *et al.* 2012 |
| CBGP | *Spodoptera ornithogalli* (Guenée) | B121* | HQ177395 | USA - Georgia | USA | Kergoat *et al.* 2012 |
| CBGP | *Spodoptera ornithogalli* (Guenée) | B146* | HQ177396 | French Guiana | GUF | Kergoat *et al.* 2012 |
| CBGP | *Spodoptera ornithogalli* (Guenée) | B28* | HQ177392 | USA - Louisiana | USA | Kergoat *et al.* 2012 |
| CBGP | *Spodoptera ornithogalli* (Guenée) | B4* | HQ177391 | USA - Louisiana | USA | Kergoat *et al.* 2012 |
| CBGP | *Spodoptera ornithogalli* (Guenée) | B54* | HQ177393 | USA - Louisiana | USA | Kergoat *et al.* 2012 |
| CBGP | *Spodoptera ornithogalli* (Guenée) | LSU34* | HQ177398 | USA - Louisiana | USA | Kergoat *et al.* 2012 |
| CBGP | *Spodoptera ornithogalli* (Guenée) | LSU8* | HQ177397 | USA | USA | Kergoat *et al.* 2012 |
| MNHN | *Spodoptera ornithogalli* (Guenée) | MNHN41** | KF854233 | Guatemala | GTM | this study |
| MNHN | *Spodoptera ornithogalli* (Guenée) | MNHN69** | KF854234 | Dominican Republic | DOM | this study |
| UM | *Spodoptera ornithogalli* (Guenée) | GBGL5878-09 | EU768964 | USA - Maryland | USA |  |
| BIO | *Spodoptera ornithogalli* (Guenée) | LOT327-04 | GU088101 | USA - Tennessee | USA |  |
| BIO | *Spodoptera ornithogalli* (Guenée) | LOT326-04 | GU088102 | USA - Tennessee | USA |  |
| UBC | *Spodoptera ornithogalli* (Guenée) | LGSM428-04 | GU090194 | USA - Tennessee | USA | Hebert *et al.* 2010 |
| UBC | *Spodoptera ornithogalli* (Guenée) | LGSM429-04 | GU090195 | USA - Tennessee | USA | Hebert *et al.* 2010 |
| UBC | *Spodoptera ornithogalli* (Guenée) | XAB643-04 | GU094314 | Canada - Ontario | CAN | Hebert *et al.* 2010 |
| BIO | *Spodoptera ornithogalli* (Guenée) | MJMSL004-10 | HQ964354 | USA - Massachusetts | USA |  |
| CTAG | *Spodoptera ornithogalli* (Guenée) | GBGL3732-06 | U72979 | USA | USA |  |
| CBGP | *Spodoptera pecten* Guenée | P1* | HQ177399 | Indonesia | IDN | Kergoat *et al*. 2012 |
| CBGP | *Spodoptera pecten* Guenée | P2* | HQ177400 | Indonesia | IDN | Kergoat *et al*. 2012 |
| CBGP | *Spodoptera pectinicornis* (Hampson) | B142* | HQ177401 | Australia | AUS | Kergoat *et al*. 2012 |
| CBGP | *Spodoptera pectinicornis* (Hampson) | B143* | HQ177402 | Australia | AUS | Kergoat *et al*. 2012 |
| CBGP | *Spodoptera pectinicornis* (Hampson) | B144* | HQ177403 | Australia | AUS | Kergoat *et al*. 2012 |
| CBGP | *Spodoptera picta* (Guérin-Méneville) | B158* | HQ177404 | Australia | AUS | Kergoat *et al*. 2012 |
| CBGP | *Spodoptera picta* (Guérin-Méneville) | B159* | HQ177405 | Australia | AUS | Kergoat *et al*. 2012 |
| ANIC | *Spodoptera picta* (Guérin-Méneville) | ANICK309-10 | KF388230 | Australia | AUS | Hebert *et al*. 2013 |
| CBGP | *Spodoptera praefica* (Grote) | B105* | HQ177408 | USA - California | USA | Kergoat *et al*. 2012 |
| CBGP | *Spodoptera praefica* (Grote) | B30* | HQ177407 | USA - California | USA | Kergoat *et al*. 2012 |
| CBGP | *Spodoptera praefica* (Grote) | B5* | HQ177406 | USA - California | USA | Kergoat *et al*. 2012 |
| CBGP | *Spodoptera praefica* (Grote) | LSU32* | HQ177409 | USA - California | USA | Kergoat *et al*. 2012 |
| USDA | *Spodoptera pulchella* (H.-Schäffer) | GBGL10113-12 | HM756075 | USA - Florida | USA | Nagoshi *et al*. 2011 |
| USDA | *Spodoptera pulchella* (H.-Schäffer) | GBGL10114-12 | HM756076 | USA - Florida | USA | Nagoshi *et al.* 2011 |
| CBGP | *Spodoptera triturata* (Walker) | GK121* | HQ177411 | Kenya | KEN | Kergoat *et al*. 2012 |
| CBGP | *Spodoptera triturata* (Walker) | Tri* | HQ177410 | Kenya | KEN | Kergoat *et al*. 2012 |
| ANIC | *Spodoptera umbraculata* (Walker) | ANICK420-10 | ANICK420-10 | Australia | AUS |  |
| ANIC | *Spodoptera umbraculata* (Walker) | ANICK419-10 | HQ950507 | Australia | AUS |  |
| ANIC | *Spodoptera umbraculata* (Walker) | ANIAG471-11 | KF388257 | Australia | AUS | Hebert *et al*. 2013 |
| ANIC | *Spodoptera umbraculata* (Walker) | ANIAG473-11 | KF391334 | Australia | AUS | Hebert *et al*. 2013 |
| ANIC | *Spodoptera umbraculata* (Walker) | ANIAG472-11 | KF392212 | Australia | AUS | Hebert *et al*. 2013 |
| ANIC | *Spodoptera umbraculata* (Walker) | ANIAG470-11 | KF393736 | Australia | AUS | Hebert *et al*. 2013 |
| MNHN | *Spodoptera umbraculata* (Walker) | MNHN84** | KF854161 | Australia | AUS | this study |
|  |  |  |  |  |  |  |
| RCW | *Agrotis ipsilon* (Hufnagel) | LBCS250-07 | FJ412140 | unknown | N/A |  |
| HUT | *Helicoverpa armigera* (Hübner) | CYTC4773-12 | GU188273 | unknown | N/A | Yin *et al.* 2010 |
| PSCAS | *Heliothis virescens* (Fabricius) | MHMXD610-06 | JQ602717 | unknown | N/A |  |
| BIO | *Mythimna unipuncta* (Haworth) | XAD347-04 | GU439027 | unknown | N/A |  |
| CIRN | *Noctua atlantica* (Warren) | GBGL1131-06 | AY600452 | unknown | N/A |  |
| UM | *Psychomorpha epimenis* (Drury) | GBGL5000-08 | EU779855 | unknown | N/A |  |
| YU | *Sesamia inferens* (Walker) | CYTC1194-12 | JN039362 | unknown | N/A |  |
|  |  |  |  |  |  |  |
