# Supplementary material for: Phylogenetic Molecular Species Delimitations Unravel Potential New Species in the Pest Genus Spodoptera Guenée, 1852 (Lepidoptera, Noctuidae)
Source: PLoS One. 2015 Apr 8;10(4):e0122407. doi: 10.1371/journal.pone.0122407 (PMC4390195; doi:10.1371/journal.pone.0122407)
Supplement: S2 Table — Three partitions were specified (one per codon position). Selected models and partitions based on the AICc are figured on the left whereas selected models and partitions based on the BIC are figured on the right. (DOCX) [file pone.0122407.s008.docx]

| **Partitions (AICc)** | **Models (AICc)** | **Partitions (BIC)** | **Models (BIC)** |
| --- | --- | --- | --- |
| #1: pos1, pos2, pos3 | GTR+G+I | #1: pos1 | GTR+G+I |
|  |  | #2: pos2 | GTR+G |
|  |  | #3: pos3 | HKY+I |
