# Supplementary material for: Phylogenetic Molecular Species Delimitations Unravel Potential New Species in the Pest Genus Spodoptera Guenée, 1852 (Lepidoptera, Noctuidae)
Source: PLoS One. 2015 Apr 8;10(4):e0122407. doi: 10.1371/journal.pone.0122407 (PMC4390195; doi:10.1371/journal.pone.0122407)
Supplement: S3 Table — The geographic origin and assignment to putative species clusters is provided for each Spodoptera specimen. Similarly to the sidebars presented on Fig 2, we have used one column per set of analyses (PTP with RAxML, PTP with MrBayes, GMYC with BEAST (strict clock) and GMYC with BEAST (UCLN clock). For more clarity, putative species clusters also are highlighted using a combination of numbers and distinct shades of grey. (DOCX) [file pone.0122407.s009.docx]

| **Species** | **Voucher no.** | **Origin** | **ISO code** | **Putative species clusters** | | | |
| --- | --- | --- | --- | --- | --- | --- | --- |
|  |  |  |  | **PTP** | **PTP** | **GMYC** | **GMYC** |
|  |  |  |  | **RAxML** | **MrBayes** | **BEAST** | **Beast** |
|  |  |  |  |  |  | **strict clock** | **UCLN** |
| *Spodoptera exigua* (Hübner) | LAUSM003-09 | Australia | AUS | **1** | **1** | **1** | **1** |
| *Spodoptera exigua* (Hübner) | LAUSM012-09 | Australia | AUS | **1** | **1** | **1** | **1** |
| *Spodoptera exigua* (Hübner) | LAUSM010-09 | Australia | AUS | **1** | **1** | **1** | **1** |
| *Spodoptera exigua* (Hübner) | LAUSM011-09 | Australia | AUS | **1** | **1** | **1** | **1** |
| *Spodoptera exigua* (Hübner) | NIAES:LEPID:199 | Japan | JAP | **2** | **2** | **2** | **2** |
| *Spodoptera exigua* (Hübner) | NIAES:LEPID:200 | Japan | JAP | **2** | **2** | **2** | **2** |
| *Spodoptera exigua* (Hübner) | NIAES:LEPID:201 | Japan | JAP | **2** | **2** | **2** | **2** |
| *Spodoptera exigua* (Hübner) | GBGL4999-08 | Laboratory colony | N/A | **2** | **2** | **2** | **2** |
| *Spodoptera exigua* (Hübner) | GBMIN38597-13 | unknown | N/A | **2** | **2** | **2** | **2** |
| *Spodoptera exigua* (Hübner) | GBMIN38646-13 | unknown | N/A | **2** | **2** | **2** | **2** |
| *Spodoptera exigua* (Hübner) | GBMIN38596-13 | unknown | N/A | **2** | **2** | **2** | **2** |
| *Spodoptera exigua* (Hübner) | GBMIN38631-13 | Thailand | THA | **2** | **2** | **2** | **2** |
| *Spodoptera exigua* (Hübner) | GBMIN38621-13 | Spain | ESP | **2** | **2** | **2** | **2** |
| *Spodoptera exigua* (Hübner) | GWORO707-09 | Germany | DEU | **2** | **2** | **2** | **2** |
| *Spodoptera exigua* (Hübner) | GBMIN30171-13 | India | IND | **2** | **2** | **2** | **2** |
| *Spodoptera exigua* (Hübner) | PHMO362-03 | Canada - Ontario | CAN | **2** | **2** | **2** | **2** |
| *Spodoptera exigua* (Hübner) | FBLMV381-09 | Germany | DEU | **2** | **2** | **2** | **2** |
| *Spodoptera exigua* (Hübner) | B34* | Kenya | KEN | **2** | **2** | **2** | **2** |
| *Spodoptera exigua* (Hübner) | B67* | USA - California | USA | **2** | **2** | **2** | **2** |
| *Spodoptera exigua* (Hübner) | B68* | Laboratory colony | N/A | **2** | **2** | **2** | **2** |
| *Spodoptera exigua* (Hübner) | B69* | Laboratory colony | N/A | **2** | **2** | **2** | **2** |
| *Spodoptera exigua* (Hübner) | G2178* | Egypt | EGY | **2** | **2** | **2** | **2** |
| *Spodoptera exigua* (Hübner) | GWORZ535-10 | Germany | DEU | **2** | **2** | **2** | **2** |
| *Spodoptera exigua* (Hübner) | G2182* | Egypt | EGY | **2** | **2** | **2** | **2** |
| *Spodoptera exigua* (Hübner) | G2185* | Egypt | EGY | **2** | **2** | **2** | **2** |
| *Spodoptera exigua* (Hübner) | GBGL10115-12 | USA - Florida | USA | **2** | **2** | **2** | **2** |
| *Spodoptera exigua* (Hübner) | GBGL10116-12 | USA - Florida | USA | **2** | **2** | **2** | **2** |
| *Spodoptera exigua* (Hübner) | GBGL10117-12 | USA - Florida | USA | **2** | **2** | **2** | **2** |
| *Spodoptera exigua* (Hübner) | GBGL10118-12 | USA - Florida | USA | **2** | **2** | **2** | **2** |
| *Spodoptera exigua* (Hübner) | LSU11* | USA | USA | **2** | **2** | **2** | **2** |
| *Spodoptera exigua* (Hübner) | LSU15* | USA- California | USA | **2** | **2** | **2** | **2** |
| *Spodoptera cilium* Guenée | GK123* | Kenya | KEN | **3** | **3** | **3** | **3** |
| *Spodoptera cilium* Guenée | GK124* | Kenya | KEN | **3** | **3** | **3** | **3** |
| *Spodoptera cilium* Guenée | GK125* | Kenya | KEN | **3** | **3** | **3** | **3** |
| *Spodoptera cilium* Guenée | NIAES:LEPID:173 | Japan | JAP | **3** | **3** | **3** | **3** |
| *Spodoptera cilium* Guenée | NIAES:LEPID:175 | Japan | JAP | **3** | **3** | **3** | **3** |
| *Spodoptera cilium* Guenée | NIAES:LEPID:176 | Japan | JAP | **3** | **3** | **3** | **3** |
| *Spodoptera cilium* Guenée | NIAES:LEPID:177 | Japan | JAP | **3** | **3** | **3** | **3** |
| *Spodoptera cilium* Guenée | NIAES:LEPID:178 | Japan | JAP | **3** | **3** | **3** | **3** |
| *Spodoptera cilium* Guenée | MAMOT956-10 | Pakistan | PAK | **3** | **3** | **3** | **3** |
| *Spodoptera cilium* Guenée | MAMOT955-10 | Pakistan | PAK | **3** | **3** | **3** | **3** |
| *Spodoptera cilium* Guenée | MAMOT954-10 | Pakistan | PAK | **3** | **3** | **3** | **3** |
| *Spodoptera cilium* Guenée | MAMOT953-10 | Pakistan | PAK | **3** | **3** | **3** | **3** |
| *Spodoptera depravata* (Butler) | GK206* | Japan | JAP | **4** | **4** | **4** | **4** |
| *Spodoptera depravata* (Butler) | NIAES:LEPID:210 | Japan | JAP | **4** | **4** | **4** | **4** |
| *Spodoptera depravata* (Butler) | NIAES:LEPID:211 | Japan | JAP | **4** | **4** | **4** | **4** |
| *Spodoptera depravata* (Butler) | JF100809166 | China | CHN | **4** | **4** | **4** | **4** |
| *Spodoptera depravata* (Butler) | LJZ100726594 | China | CHN | **4** | **4** | **4** | **4** |
| *Spodoptera depravata* (Butler) | LJZ100726665 | China | CHN | **4** | **4** | **4** | **4** |
| *Spodoptera depravata* (Butler) | LTOLB1216-11 | Japan | JAP | **4** | **4** | **4** | **4** |
| *Spodoptera umbraculata* (Walker) | ANICK420-10 | Australia | AUS | **5** | **5** | **5** | **5** |
| *Spodoptera umbraculata* (Walker) | ANICK419-10 | Australia | AUS | **5** | **5** | **5** | **5** |
| *Spodoptera umbraculata* (Walker) | ANIAG471-11 | Australia | AUS | **5** | **5** | **5** | **5** |
| *Spodoptera umbraculata* (Walker) | ANIAG473-11 | Australia | AUS | **5** | **5** | **5** | **5** |
| *Spodoptera umbraculata* (Walker) | ANIAG472-11 | Australia | AUS | **5** | **5** | **5** | **5** |
| *Spodoptera umbraculata* (Walker) | ANIAG470-11 | Australia | AUS | **5** | **5** | **5** | **5** |
| *Spodoptera umbraculata* (Walker) | MNHN84** | Australia | AUS | **5** | **5** | **5** | **5** |
| *Spodoptera pecten* Guenée | P1* | Indonesia | IDN | **6** | **6** | **6** | **6** |
| *Spodoptera pecten* Guenée | P2* | Indonesia | IDN | **6** | **6** | **6** | **6** |
| *Spodoptera exempta* (Walker) | B162* | Kenya | KEN | **7** | **7** | **7** | **7** |
| *Spodoptera exempta* (Walker) | B163* | Kenya | KEN | **7** | **7** | **7** | **7** |
| *Spodoptera exempta* (Walker) | B24* | Laboratory colony | N/A | **7** | **7** | **7** | **7** |
| *Spodoptera exempta* (Walker) | B25* | Laboratory colony | N/A | **7** | **7** | **7** | **7** |
| *Spodoptera exempta* (Walker) | B93* | Laboratory colony | N/A | **7** | **7** | **7** | **7** |
| *Spodoptera exempta* (Walker) | NIAES:LEPID:247 | Japan | JAP | **7** | **7** | **7** | **7** |
| *Spodoptera exempta* (Walker) | NIAES:LEPID:248 | Japan | JAP | **7** | **7** | **7** | **7** |
| *Spodoptera exempta* (Walker) | NIAES:LEPID:249 | Japan | JAP | **7** | **7** | **7** | **7** |
| *Spodoptera exempta* (Walker) | NIAES:LEPID:251 | Japan | JAP | **7** | **7** | **7** | **7** |
| *Spodoptera exempta* (Walker) | NIAES:LEPID:252 | Japan | JAP | **7** | **7** | **7** | **7** |
| *Spodoptera exempta* (Walker) | NIAES:LEPID:253 | Japan | JAP | **7** | **7** | **7** | **7** |
| *Spodoptera exempta* (Walker) | NIAES:LEPID:254 | Japan | JAP | **7** | **7** | **7** | **7** |
| *Spodoptera exempta* (Walker) | NIAES:LEPID:255 | Japan | JAP | **7** | **7** | **7** | **7** |
| *Spodoptera exempta* (Walker) | NIAES:LEPID:256 | Japan | JAP | **7** | **7** | **7** | **7** |
| *Spodoptera exempta* (Walker) | NIAES:LEPID:257 | Japan | JAP | **7** | **7** | **7** | **7** |
| *Spodoptera exempta* (Walker) | NIAES:LEPID:258 | Japan | JAP | **7** | **7** | **7** | **7** |
| *Spodoptera exempta* (Walker) | NIAES:LEPID:260 | Japan | JAP | **7** | **7** | **7** | **7** |
| *Spodoptera exempta* (Walker) | NIAES:LEPID:261 | Japan | JAP | **7** | **7** | **7** | **7** |
| *Spodoptera exempta* (Walker) | NIAES:LEPID:262 | Japan | JAP | **7** | **7** | **7** | **7** |
| *Spodoptera exempta* (Walker) | NIAES:LEPID:263 | Japan | JAP | **7** | **7** | **7** | **7** |
| *Spodoptera exempta* (Walker) | NIAES:LEPID:264 | Japan | JAP | **7** | **7** | **7** | **7** |
| *Spodoptera exempta* (Walker) | NIAES:LEPID:265 | Japan | JAP | **7** | **7** | **7** | **7** |
| *Spodoptera exempta* (Walker) | NIAES:LEPID:266 | Japan | JAP | **7** | **7** | **7** | **7** |
| *Spodoptera exempta* (Walker) | NIAES:LEPID:267 | Japan | JAP | **7** | **7** | **7** | **7** |
| *Spodoptera exempta* (Walker) | NIAES:LEPID:268 | Japan | JAP | **7** | **7** | **7** | **7** |
| *Spodoptera exempta* (Walker) | NIAES:LEPID:269 | Japan | JAP | **7** | **7** | **7** | **7** |
| *Spodoptera exempta* (Walker) | NIAES:LEPID:270 | Japan | JAP | **7** | **7** | **7** | **7** |
| *Spodoptera exempta* (Walker) | NIAES:LEPID:271 | Japan | JAP | **7** | **7** | **7** | **7** |
| *Spodoptera exempta* (Walker) | NIAES:LEPID:272 | Japan | JAP | **7** | **7** | **7** | **7** |
| *Spodoptera exempta* (Walker) | NIAES:LEPID:273 | Japan | JAP | **7** | **7** | **7** | **7** |
| *Spodoptera exempta* (Walker) | NIAES:LEPID:275 | Japan | JAP | **7** | **7** | **7** | **7** |
| *Spodoptera exempta* (Walker) | NIAES:LEPID:276 | Japan | JAP | **7** | **7** | **7** | **7** |
| *Spodoptera exempta* (Walker) | NIAES:LEPID:143 | Japan | JAP | **7** | **7** | **7** | **7** |
| *Spodoptera exempta* (Walker) | NIAES:LEPID:144 | Japan | JAP | **7** | **7** | **7** | **7** |
| *Spodoptera exempta* (Walker) | NIAES:LEPID:145 | Japan | JAP | **7** | **7** | **7** | **7** |
| *Spodoptera exempta* (Walker) | NIAES:LEPID:146 | Japan | JAP | **7** | **7** | **7** | **7** |
| *Spodoptera exempta* (Walker) | NIAES:LEPID:147 | Japan | JAP | **7** | **7** | **7** | **7** |
| *Spodoptera exempta* (Walker) | NIAES:LEPID:148 | Japan | JAP | **7** | **7** | **7** | **7** |
| *Spodoptera exempta* (Walker) | NIAES:LEPID:149 | Japan | JAP | **7** | **7** | **7** | **7** |
| *Spodoptera exempta* (Walker) | NIAES:LEPID:162 | Japan | JAP | **7** | **7** | **7** | **7** |
| *Spodoptera exempta* (Walker) | NIAES:LEPID:163 | Japan | JAP | **7** | **7** | **7** | **7** |
| *Spodoptera exempta* (Walker) | NIAES:LEPID:164 | Japan | JAP | **7** | **7** | **7** | **7** |
| *Spodoptera exempta* (Walker) | NIAES:LEPID:165 | Japan | JAP | **7** | **7** | **7** | **7** |
| *Spodoptera exempta* (Walker) | NIAES:LEPID:166 | Japan | JAP | **7** | **7** | **7** | **7** |
| *Spodoptera exempta* (Walker) | NIAES:LEPID:167 | Japan | JAP | **7** | **7** | **7** | **7** |
| *Spodoptera exempta* (Walker) | NIAES:LEPID:274 | Japan | JAP | **7** | **7** | **7** | **7** |
| *Spodoptera exempta* (Walker) | MGABB511-10 | Gabon | GAB | **7** | **7** | **7** | **7** |
| *Spodoptera exempta* (Walker) | GBMIN17394-13 | Tanzania | TZA | **7** | **7** | **7** | **7** |
| *Spodoptera exempta* (Walker) | GBMIN17302-13 | Tanzania | TZA | **7** | **7** | **7** | **7** |
| *Spodoptera exempta* (Walker) | GBMIN17393-13 | Tanzania | TZA | **7** | **7** | **7** | **7** |
| *Spodoptera exempta* (Walker) | GBMIN17301-13 | Tanzania | TZA | **7** | **7** | **7** | **7** |
| *Spodoptera exempta* (Walker) | GBMIN17392-13 | Tanzania | TZA | **7** | **7** | **7** | **7** |
| *Spodoptera exempta* (Walker) | GBMIN17300-13 | Tanzania | TZA | **7** | **7** | **7** | **7** |
| *Spodoptera exempta* (Walker) | GBMIN17391-13 | Tanzania | TZA | **7** | **7** | **7** | **7** |
| *Spodoptera exempta* (Walker) | GBMIN17299-13 | Tanzania | TZA | **7** | **7** | **7** | **7** |
| *Spodoptera exempta* (Walker) | GBMIN17390-13 | Tanzania | TZA | **7** | **7** | **7** | **7** |
| *Spodoptera exempta* (Walker) | GBMIN17298-13 | Tanzania | TZA | **7** | **7** | **7** | **7** |
| *Spodoptera exempta* (Walker) | LPNGM110-07 | Papua New Guinea | PNG | **7** | **7** | **7** | **7** |
| *Spodoptera exempta* (Walker) | LPNGM108-07 | Papua New Guinea | PNG | **7** | **7** | **7** | **7** |
| *Spodoptera exempta* (Walker) | LPNGM107-07 | Papua New Guinea | PNG | **7** | **7** | **7** | **7** |
| *Spodoptera triturata* (Walker) | GK121* | Kenya | KEN | **8** | **8** | **8** | **8** |
| *Spodoptera triturata* (Walker) | Tri* | Kenya | KEN | **8** | **8** | **8** | **8** |
| *Spodoptera mauritia* (Boisduval) | NIAES:LEPID:181 | Japan | JAP | **9** | **9** | **9** | **9** |
| *Spodoptera mauritia* (Boisduval) | NIAES:LEPID:182 | Japan | JAP | **9** | **9** | **9** | **9** |
| *Spodoptera mauritia* (Boisduval) | NIAES:LEPID:183 | Japan | JAP | **9** | **9** | **9** | **9** |
| *Spodoptera mauritia* (Boisduval) | ANICK415-10 | Australia | AUS | **9** | **9** | **9** | **9** |
| *Spodoptera mauritia* (Boisduval) | B152* | Papua New Guinea | PNG | **9** | **9** | **9** | **9** |
| *Spodoptera mauritia* (Boisduval) | B65* | Reunion | REU | **10** | **10** | **10** | **10** |
| *Spodoptera mauritia* (Boisduval) | B66* | Reunion | REU | **10** | **10** | **10** | **10** |
| *Spodoptera mauritia* (Boisduval) | B94* | Reunion | REU | **10** | **10** | **10** | **10** |
| *Spodoptera ochrea* (Hampson) | B110* | Peru | PER | **11** | **11** | **11** | **11** |
| *Spodoptera ochrea* (Hampson) | B84* | Peru | PER | **11** | **11** | **11** | **11** |
| *Spodoptera ochrea* (Hampson) | B85* | Peru | PER | **11** | **11** | **11** | **11** |
| *Spodoptera ochrea* (Hampson) | B86* | Peru | PER | **11** | **11** | **11** | **11** |
| *Spodoptera ochrea* (Hampson) | MNHN75** | Peru | PER | **11** | **11** | **11** | **11** |
| *Spodoptera albula* (Walker) | B20* | French Guiana | GUF | **12** | **12** | **12** | **12** |
| *Spodoptera albula* (Walker) | B53* | Honduras | HND | **12** | **12** | **12** | **12** |
| *Spodoptera albula* (Walker) | LSU16* | French Guiana | GUF | **12** | **12** | **12** | **12** |
| *Spodoptera albula* (Walker) | LSU24* | USA - Louisiana | USA | **12** | **12** | **12** | **12** |
| *Spodoptera albula* (Walker) | MNHN26** | Venezuela | VEN | **12** | **12** | **12** | **12** |
| *Spodoptera albula* (Walker) | MNHN27** | Jamaica | JAM | **12** | **12** | **12** | **12** |
| *Spodoptera albula* (Walker) | MNHN30** | Panama | PAN | **12** | **12** | **12** | **12** |
| *Spodoptera albula* (Walker) | GBGL12664-13 | Brazil | BRA | **12** | **12** | **12** | **12** |
| *Spodoptera albula* (Walker) | GBGL12663-13 | Brazil | BRA | **12** | **12** | **12** | **12** |
| *Spodoptera albula* (Walker) | GBGL12662-13 | Brazil | BRA | **12** | **12** | **12** | **12** |
| *Spodoptera albula* (Walker) | GBGL12661-13 | Brazil | BRA | **12** | **12** | **12** | **12** |
| *Spodoptera albula* (Walker) | GBGL12660-13 | Brazil | BRA | **12** | **12** | **12** | **12** |
| *Spodoptera albula* (Walker) | GBGL12659-13 | Brazil | BRA | **12** | **12** | **12** | **12** |
| *Spodoptera albula* (Walker) | GBGL12658-13 | Brazil | BRA | **12** | **12** | **12** | **12** |
| *Spodoptera albula* (Walker) | GBGL12657-13 | Brazil | BRA | **12** | **12** | **12** | **12** |
| *Spodoptera albula* (Walker) | GBGL12656-13 | Brazil | BRA | **12** | **12** | **12** | **12** |
| *Spodoptera albula* (Walker) | GBGL12655-13 | Brazil | BRA | **12** | **12** | **12** | **12** |
| *Spodoptera albula* (Walker) | GBGL12654-13 | Brazil | BRA | **12** | **12** | **12** | **12** |
| *Spodoptera albula* (Walker) | GBGL12653-13 | Brazil | BRA | **12** | **12** | **12** | **12** |
| *Spodoptera albula* (Walker) | GBGL12652-13 | Brazil | BRA | **12** | **12** | **12** | **12** |
| *Spodoptera albula* (Walker) | GBGL12651-13 | Brazil | BRA | **12** | **12** | **12** | **12** |
| *Spodoptera albula* (Walker) | GBGL12650-13 | Brazil | BRA | **12** | **12** | **12** | **12** |
| *Spodoptera albula* (Walker) | GBGL12649-13 | Brazil | BRA | **12** | **12** | **12** | **12** |
| *Spodoptera albula* (Walker) | GBGL12648-13 | Brazil | BRA | **12** | **12** | **12** | **12** |
| *Spodoptera albula* (Walker) | GBGL12647-13 | Brazil | BRA | **12** | **12** | **12** | **12** |
| *Spodoptera eridania* (Stoll) | B108* | Peru | PER | **13** | **13** | **13** | **13** |
| *Spodoptera eridania* (Stoll) | B109* | Peru | PER | **13** | **13** | **13** | **13** |
| *Spodoptera eridania* (Stoll) | B16* | USA - Louisiana | USA | **13** | **13** | **13** | **13** |
| *Spodoptera eridania* (Stoll) | B47* | Guadeloupe | GLP | **13** | **13** | **13** | **13** |
| *Spodoptera eridania* (Stoll) | B70* | USA - Louisiana | USA | **13** | **13** | **13** | **13** |
| *Spodoptera eridania* (Stoll) | B71* | USA - Louisiana | USA | **13** | **13** | **13** | **13** |
| *Spodoptera eridania* (Stoll) | B72* | USA - Louisiana | USA | **13** | **13** | **13** | **13** |
| *Spodoptera eridania* (Stoll) | B73* | USA - Louisiana | USA | **13** | **13** | **13** | **13** |
| *Spodoptera eridania* (Stoll) | B75* | Guadeloupe | GLP | **13** | **13** | **13** | **13** |
| *Spodoptera eridania* (Stoll) | GBGL10119-12 | USA - Florida | USA | **13** | **13** | **13** | **13** |
| *Spodoptera eridania* (Stoll) | GBGL10120-12 | USA - Florida | USA | **13** | **13** | **13** | **13** |
| *Spodoptera eridania* (Stoll) | GBGL10121-12 | USA - Florida | USA | **13** | **13** | **13** | **13** |
| *Spodoptera eridania* (Stoll) | GBGL10122-12 | USA - Florida | USA | **13** | **13** | **13** | **13** |
| *Spodoptera eridania* (Stoll) | GBGL10123-12 | USA - Florida | USA | **13** | **13** | **13** | **13** |
| *Spodoptera eridania* (Stoll) | LSU5* | USA | USA | **13** | **13** | **13** | **13** |
| *Spodoptera eridania* (Stoll) | MHAUB899-05 | Costa Rica | CRI | **13** | **13** | **13** | **13** |
| *Spodoptera eridania* (Stoll) | MHAUB898-05 | Costa Rica | CRI | **13** | **13** | **13** | **13** |
| *Spodoptera eridania* (Stoll) | MHAUB897-05 | Costa Rica | CRI | **13** | **13** | **13** | **13** |
| *Spodoptera eridania* (Stoll) | MHAUB895-05 | Costa Rica | CRI | **13** | **13** | **13** | **13** |
| *Spodoptera eridania* (Stoll) | MHAUB896-05 | Costa Rica | CRI | **13** | **13** | **13** | **13** |
| *Spodoptera eridania* (Stoll) | BLPCK258-08 | Costa Rica | CRI | **13** | **13** | **13** | **13** |
| *Spodoptera eridania* (Stoll) | BLPEC679-11 | Costa Rica | CRI | **13** | **13** | **13** | **13** |
| *Spodoptera eridania* (Stoll) | BLPDA469-09 | Costa Rica | CRI | **13** | **13** | **13** | **13** |
| *Spodoptera eridania* (Stoll) | BLPDC450-09 | Costa Rica | CRI | **13** | **13** | **13** | **13** |
| *Spodoptera eridania* (Stoll) | MHMXF638-07 | Costa Rica | CRI | **13** | **13** | **13** | **13** |
| *Spodoptera eridania* (Stoll) | MHMXI219-07 | Costa Rica | CRI | **13** | **13** | **13** | **13** |
| *Spodoptera eridania* (Stoll) | GBGL12646-13 | Brazil | BRA | **13** | **13** | **13** | **13** |
| *Spodoptera eridania* (Stoll) | GBGL12645-13 | Brazil | BRA | **13** | **13** | **13** | **13** |
| *Spodoptera eridania* (Stoll) | GBGL12644-13 | Brazil | BRA | **13** | **13** | **13** | **13** |
| *Spodoptera eridania* (Stoll) | LEMMZ129-10 | Brazil | BRA | **13** | **13** | **13** | **13** |
| *Spodoptera eridania* (Stoll) | GBGL12643-13 | Brazil | BRA | **13** | **13** | **13** | **13** |
| *Spodoptera eridania* (Stoll) | GBGL12642-13 | Brazil | BRA | **13** | **13** | **13** | **13** |
| *Spodoptera eridania* (Stoll) | GBGL12641-13 | Brazil | BRA | **13** | **13** | **13** | **13** |
| *Spodoptera eridania* (Stoll) | GBGL12640-13 | Brazil | BRA | **13** | **13** | **13** | **13** |
| *Spodoptera eridania* (Stoll) | GBGL12639-13 | Brazil | BRA | **13** | **13** | **13** | **13** |
| *Spodoptera eridania* (Stoll) | GBGL12638-13 | Brazil | BRA | **13** | **13** | **13** | **13** |
| *Spodoptera eridania* (Stoll) | GBGL12637-13 | Brazil | BRA | **13** | **13** | **13** | **13** |
| *Spodoptera eridania* (Stoll) | GBGL12636-13 | Brazil | BRA | **13** | **13** | **13** | **13** |
| *Spodoptera eridania* (Stoll) | GBGL12635-13 | Brazil | BRA | **13** | **13** | **13** | **13** |
| *Spodoptera eridania* (Stoll) | GBGL12634-13 | Brazil | BRA | **13** | **13** | **13** | **13** |
| *Spodoptera eridania* (Stoll) | GBGL12633-13 | Brazil | BRA | **13** | **13** | **13** | **13** |
| *Spodoptera eridania* (Stoll) | GBGL12632-13 | Brazil | BRA | **13** | **13** | **13** | **13** |
| *Spodoptera eridania* (Stoll) | GBGL12631-13 | Brazil | BRA | **13** | **13** | **13** | **13** |
| *Spodoptera eridania* (Stoll) | GBGL12630-13 | Brazil | BRA | **13** | **13** | **13** | **13** |
| *Spodoptera apertura* (Walker) | ANICK313-10 | Australia | AUS | **14** | **14** | **14** | **14** |
| *Spodoptera apertura* (Walker) | ANICK315-10 | Australia | AUS | **15** | **14** | **14** | **14** |
| *Spodoptera frugiperda* (J.E. Smith) | B90C* | Peru | PER | **16** | **15** | **15** | **15** |
| *Spodoptera frugiperda* (J.E. Smith) | FRUB1** | Brazil | BRA | **16** | **15** | **15** | **15** |
| *Spodoptera frugiperda* (J.E. Smith) | FRUB11** | Brazil | BRA | **16** | **15** | **15** | **15** |
| *Spodoptera frugiperda* (J.E. Smith) | FRUB12** | Brazil | BRA | **16** | **15** | **15** | **15** |
| *Spodoptera frugiperda* (J.E. Smith) | FRUB13** | Brazil | BRA | **16** | **15** | **15** | **15** |
| *Spodoptera frugiperda* (J.E. Smith) | FRUB14** | Brazil | BRA | **16** | **15** | **15** | **15** |
| *Spodoptera frugiperda* (J.E. Smith) | FRUB15** | Brazil | BRA | **16** | **15** | **15** | **15** |
| *Spodoptera frugiperda* (J.E. Smith) | FRUB16** | Brazil | BRA | **16** | **15** | **15** | **15** |
| *Spodoptera frugiperda* (J.E. Smith) | FRUB17** | Brazil | BRA | **16** | **15** | **15** | **15** |
| *Spodoptera frugiperda* (J.E. Smith) | FRUB3** | Brazil | BRA | **16** | **15** | **15** | **15** |
| *Spodoptera frugiperda* (J.E. Smith) | FRUB4** | Brazil | BRA | **16** | **15** | **15** | **15** |
| *Spodoptera frugiperda* (J.E. Smith) | FRUB7** | Brazil | BRA | **16** | **15** | **15** | **15** |
| *Spodoptera frugiperda* (J.E. Smith) | FRUB8** | Brazil | BRA | **16** | **15** | **15** | **15** |
| *Spodoptera frugiperda* (J.E. Smith) | G1 135** | Guadeloupe | GLP | **16** | **15** | **15** | **15** |
| *Spodoptera frugiperda* (J.E. Smith) | G4 153** | Guadeloupe | GLP | **16** | **15** | **15** | **15** |
| *Spodoptera frugiperda* (J.E. Smith) | GBGL10069-12 | USA - Florida | USA | **16** | **15** | **15** | **15** |
| *Spodoptera frugiperda* (J.E. Smith) | GBGL10070-12 | USA - Florida | USA | **16** | **15** | **15** | **15** |
| *Spodoptera frugiperda* (J.E. Smith) | GBGL10071-12 | USA - Florida | USA | **16** | **15** | **15** | **15** |
| *Spodoptera frugiperda* (J.E. Smith) | GBGL10072-12 | USA - Florida | USA | **16** | **15** | **15** | **15** |
| *Spodoptera frugiperda* (J.E. Smith) | GBGL10073-12 | USA - Florida | USA | **16** | **15** | **15** | **15** |
| *Spodoptera frugiperda* (J.E. Smith) | GBGL10074-12 | USA - Florida | USA | **16** | **15** | **15** | **15** |
| *Spodoptera frugiperda* (J.E. Smith) | GBGL10075-12 | USA - Florida | USA | **16** | **15** | **15** | **15** |
| *Spodoptera frugiperda* (J.E. Smith) | LSU21* | Guadeloupe | GLP | **16** | **15** | **15** | **15** |
| *Spodoptera frugiperda* (J.E. Smith) | MNHN12** | Paraguay | PRY | **16** | **15** | **15** | **15** |
| *Spodoptera frugiperda* (J.E. Smith) | MNHN14** | Dominican Repubic | DOM | **16** | **15** | **15** | **15** |
| *Spodoptera frugiperda* (J.E. Smith) | MNHN16** | Dominican Repubic | DOM | **16** | **15** | **15** | **15** |
| *Spodoptera frugiperda* (J.E. Smith) | MNHN56** | Cuba | CUB | **16** | **15** | **15** | **15** |
| *Spodoptera frugiperda* (J.E. Smith) | MNHN81** | Peru | PER | **16** | **15** | **15** | **15** |
| *Spodoptera frugiperda* (J.E. Smith) | 311 MF67** | French Guiana | GUF | **17** | **16** | **16** | **16** |
| *Spodoptera frugiperda* (J.E. Smith) | 337 MR7** | French Guiana | GUF | **17** | **16** | **16** | **16** |
| *Spodoptera frugiperda* (J.E. Smith) | B10R* | French Guiana | GUF | **17** | **16** | **16** | **16** |
| *Spodoptera frugiperda* (J.E. Smith) | B12R* | French Guiana | GUF | **17** | **16** | **16** | **16** |
| *Spodoptera frugiperda* (J.E. Smith) | B7R* | French Guiana | GUF | **17** | **16** | **16** | **16** |
| *Spodoptera frugiperda* (J.E. Smith) | CE1** | French Guiana | GUF | **17** | **16** | **16** | **16** |
| *Spodoptera frugiperda* (J.E. Smith) | F1 329** | French Guiana | GUF | **17** | **16** | **16** | **16** |
| *Spodoptera frugiperda* (J.E. Smith) | F1 330** | French Guiana | GUF | **17** | **16** | **16** | **16** |
| *Spodoptera frugiperda* (J.E. Smith) | F4 322** | French Guiana | GUF | **17** | **16** | **16** | **16** |
| *Spodoptera frugiperda* (J.E. Smith) | FRUB10** | Brazil | BRA | **17** | **16** | **16** | **16** |
| *Spodoptera frugiperda* (J.E. Smith) | FRUB9** | Brazil | BRA | **17** | **16** | **16** | **16** |
| *Spodoptera frugiperda* (J.E. Smith) | G9 305** | French Guiana | GUF | **17** | **16** | **16** | **16** |
| *Spodoptera frugiperda* (J.E. Smith) | G9 308** | French Guiana | GUF | **17** | **16** | **16** | **16** |
| *Spodoptera frugiperda* (J.E. Smith) | GK429** | Brazil | BRA | **17** | **16** | **16** | **16** |
| *Spodoptera frugiperda* (J.E. Smith) | GBGL10076-12 | USA - Florida | USA | **17** | **16** | **16** | **16** |
| *Spodoptera frugiperda* (J.E. Smith) | GBGL10077-12 | USA - Florida | USA | **17** | **16** | **16** | **16** |
| *Spodoptera frugiperda* (J.E. Smith) | GBGL10078-12 | USA - Florida | USA | **17** | **16** | **16** | **16** |
| *Spodoptera frugiperda* (J.E. Smith) | GBGL10079-12 | USA - Florida | USA | **17** | **16** | **16** | **16** |
| *Spodoptera frugiperda* (J.E. Smith) | GBGL10080-12 | USA - Florida | USA | **17** | **16** | **16** | **16** |
| *Spodoptera frugiperda* (J.E. Smith) | GBGL10081-12 | USA - Florida | USA | **17** | **16** | **16** | **16** |
| *Spodoptera frugiperda* (J.E. Smith) | LSU22R* | Guadeloupe | GLP | **17** | **16** | **16** | **16** |
| *Spodoptera frugiperda* (J.E. Smith) | MFG 50** | French Guiana | GUF | **17** | **16** | **16** | **16** |
| *Spodoptera frugiperda* (J.E. Smith) | MFG 56** | French Guiana | GUF | **17** | **16** | **16** | **16** |
| *Spodoptera frugiperda* (J.E. Smith) | MNHN10** | Dominican Repubic | DOM | **17** | **16** | **16** | **16** |
| *Spodoptera frugiperda* (J.E. Smith) | MNHN11** | Venezuela | VEN | **17** | **16** | **16** | **16** |
| *Spodoptera frugiperda* (J.E. Smith) | MNHN13** | Mexico | MEX | **17** | **16** | **16** | **16** |
| *Spodoptera frugiperda* (J.E. Smith) | MNHN15** | Guatemala | GTM | **17** | **16** | **16** | **16** |
| *Spodoptera frugiperda* (J.E. Smith) | MNHN9** | Dominican Repubic | DOM | **17** | **16** | **16** | **16** |
| *Spodoptera frugiperda* (J.E. Smith) | GBGL5879-09 | unknown | N/A | **17** | **16** | **16** | **16** |
| *Spodoptera frugiperda* (J.E. Smith) | XAH548-05 | Canada - Ontario | CAN | **17** | **16** | **16** | **16** |
| *Spodoptera frugiperda* (J.E. Smith) | XAH550-05 | Canada - Ontario | CAN | **17** | **16** | **16** | **16** |
| *Spodoptera frugiperda* (J.E. Smith) | PHMO299-03 | Canada - New Brunswick | CAN | **17** | **16** | **16** | **16** |
| *Spodoptera frugiperda* (J.E. Smith) | PHMO305-03 | Canada - New Brunswick | CAN | **17** | **16** | **16** | **16** |
| *Spodoptera frugiperda* (J.E. Smith) | PHMO358-03 | Canada - New Brunswick | CAN | **17** | **16** | **16** | **16** |
| *Spodoptera frugiperda* (J.E. Smith) | MNBB563-05 | Canada - New Brunswick | CAN | **17** | **16** | **16** | **16** |
| *Spodoptera frugiperda* (J.E. Smith) | MHAUB890-05 | Costa Rica | CRI | **17** | **16** | **16** | **16** |
| *Spodoptera frugiperda* (J.E. Smith) | MHAUB893-05 | Costa Rica | CRI | **17** | **16** | **16** | **16** |
| *Spodoptera frugiperda* (J.E. Smith) | MHAUB892-05 | Costa Rica | CRI | **17** | **16** | **16** | **16** |
| *Spodoptera frugiperda* (J.E. Smith) | MHAUB886-05 | Costa Rica | CRI | **17** | **16** | **16** | **16** |
| *Spodoptera frugiperda* (J.E. Smith) | MHAUB888-05 | Costa Rica | CRI | **17** | **16** | **16** | **16** |
| *Spodoptera frugiperda* (J.E. Smith) | MHAUB889-05 | Costa Rica | CRI | **17** | **16** | **16** | **16** |
| *Spodoptera frugiperda* (J.E. Smith) | MHAUB887-05 | Costa Rica | CRI | **17** | **16** | **16** | **16** |
| *Spodoptera frugiperda* (J.E. Smith) | MHAUB885-05 | Costa Rica | CRI | **17** | **16** | **16** | **16** |
| *Spodoptera frugiperda* (J.E. Smith) | MHAUB891-05 | Costa Rica | CRI | **17** | **16** | **16** | **16** |
| *Spodoptera frugiperda* (J.E. Smith) | MHAUB894-05 | Costa Rica | CRI | **17** | **16** | **16** | **16** |
| *Spodoptera frugiperda* (J.E. Smith) | MHAUF155-06 | Costa Rica | CRI | **17** | **16** | **16** | **16** |
| *Spodoptera frugiperda* (J.E. Smith) | XAD236-04 | Canada - Ontario | CAN | **17** | **16** | **16** | **16** |
| *Spodoptera frugiperda* (J.E. Smith) | XAD239-04 | Canada - Ontario | CAN | **17** | **16** | **16** | **16** |
| *Spodoptera frugiperda* (J.E. Smith) | XAD265-04 | Canada - Ontario | CAN | **17** | **16** | **16** | **16** |
| *Spodoptera frugiperda* (J.E. Smith) | XAD490-04 | Canada - Ontario | CAN | **17** | **16** | **16** | **16** |
| *Spodoptera frugiperda* (J.E. Smith) | XAD509-04 | Canada - Ontario | CAN | **17** | **16** | **16** | **16** |
| *Spodoptera frugiperda* (J.E. Smith) | MHMYE1162-09 | Costa Rica | CRI | **17** | **16** | **16** | **16** |
| *Spodoptera frugiperda* (J.E. Smith) | MJMSL002-10 | USA - Massachusetts | USA | **17** | **16** | **16** | **16** |
| *Spodoptera frugiperda* (J.E. Smith) | MJMSL151-10 | USA - Massachusetts | USA | **17** | **16** | **16** | **16** |
| *Spodoptera frugiperda* (J.E. Smith) | LEMMZ127-10 | Brazil | BRA | **17** | **16** | **16** | **16** |
| *Spodoptera frugiperda* (J.E. Smith) | LEMMZ128-10 | Brazil | BRA | **17** | **16** | **16** | **16** |
| *Spodoptera frugiperda* (J.E. Smith) | LEMMZ130-10 | Brazil | BRA | **17** | **16** | **16** | **16** |
| *Spodoptera frugiperda* (J.E. Smith) | LEMMZ131-10 | Brazil | BRA | **17** | **16** | **16** | **16** |
| *Spodoptera frugiperda* (J.E. Smith) | LEMMZ132-10 | Brazil | BRA | **17** | **16** | **16** | **16** |
| *Spodoptera frugiperda* (J.E. Smith) | LEMMZ133-10 | Brazil | BRA | **17** | **16** | **16** | **16** |
| *Spodoptera frugiperda* (J.E. Smith) | LEMMZ134-10 | Brazil | BRA | **17** | **16** | **16** | **16** |
| *Spodoptera frugiperda* (J.E. Smith) | BLPBB657-07 | Costa Rica | CRI | **17** | **16** | **16** | **16** |
| *Spodoptera frugiperda* (J.E. Smith) | IBOLG016-08 | Costa Rica | CRI | **17** | **16** | **16** | **16** |
| *Spodoptera frugiperda* (J.E. Smith) | BLPDE131-09 | Costa Rica | CRI | **17** | **16** | **16** | **16** |
| *Spodoptera frugiperda* (J.E. Smith) | BLPCC401-08 | Costa Rica | CRI | **17** | **16** | **16** | **16** |
| *Spodoptera frugiperda* (J.E. Smith) | BLPAG434-07 | Costa Rica | CRI | **17** | **16** | **16** | **16** |
| *Spodoptera frugiperda* (J.E. Smith) | BLPBA709-07 | Costa Rica | CRI | **17** | **16** | **16** | **16** |
| *Spodoptera frugiperda* (J.E. Smith) | BLPAB120-06 | Costa Rica | CRI | **17** | **16** | **16** | **16** |
| *Spodoptera frugiperda* (J.E. Smith) | GBGL3727-06 | unknown | N/A | **17** | **16** | **16** | **16** |
| *Spodoptera frugiperda* (J.E. Smith) | GBGL3728-06 | unknown | N/A | **17** | **16** | **16** | **16** |
| *Spodoptera frugiperda* (J.E. Smith) | GBGL3729-06 | unknown | N/A | **17** | **16** | **16** | **16** |
| *Spodoptera frugiperda* (J.E. Smith) | GBGL3730-06 | unknown | N/A | **17** | **16** | **16** | **16** |
| *Spodoptera frugiperda* (J.E. Smith) | GBGL3731-06 | unknown | N/A | **17** | **16** | **16** | **16** |
| *Spodoptera pectinicornis* (Hampson) | B142* | Australia | AUS | **18** | **17** | **17** | **17** |
| *Spodoptera pectinicornis* (Hampson) | B143* | Australia | AUS | **18** | **17** | **17** | **17** |
| *Spodoptera pectinicornis* (Hampson) | B144* | Australia | AUS | **18** | **17** | **17** | **17** |
| *Spodoptera picta* (Guérin-Méneville) | B158* | Australia | AUS | **19** | **18** | **18** | **18** |
| *Spodoptera picta* (Guérin-Méneville) | B159* | Australia | AUS | **19** | **18** | **18** | **18** |
| *Spodoptera picta* (Guérin-Méneville) | ANICK309-10 | Australia | AUS | **19** | **18** | **18** | **18** |
| *Spodoptera littoralis* (Boisduval) | GBMIN38593-13 | unknown | N/A | **20** | **19** | **19** | **19** |
| *Spodoptera littoralis* (Boisduval) | GBMIN38642-13 | unknown | N/A | **20** | **19** | **19** | **19** |
| *Spodoptera littoralis* (Boisduval) | GBMIN38592-13 | unknown | N/A | **20** | **19** | **19** | **19** |
| *Spodoptera littoralis* (Boisduval) | GBMIN38641-13 | unknown | N/A | **20** | **19** | **19** | **19** |
| *Spodoptera littoralis* (Boisduval) | GBMIN38587-13 | unknown | N/A | **20** | **19** | **19** | **19** |
| *Spodoptera littoralis* (Boisduval) | GBMIN38580-13 | Viet Nam | VNM | **20** | **19** | **19** | **19** |
| *Spodoptera littoralis* (Boisduval) | GBMIN38629-13 | Ghana | GHA | **20** | **19** | **19** | **19** |
| *Spodoptera littoralis* (Boisduval) | GBMIN38579-13 | unknown | N/A | **20** | **19** | **19** | **19** |
| *Spodoptera littoralis* (Boisduval) | GBMIN38628-13 | Nigeria | NGA | **20** | **19** | **19** | **19** |
| *Spodoptera littoralis* (Boisduval) | GBMIN38627-13 | Israel | ISR | **20** | **19** | **19** | **19** |
| *Spodoptera littoralis* (Boisduval) | GBMIN38574-13 | Israel | ISR | **20** | **19** | **19** | **19** |
| *Spodoptera littoralis* (Boisduval) | ADN0* | Egypt | EGY | **20** | **19** | **19** | **19** |
| *Spodoptera littoralis* (Boisduval) | B14* | Laboratory colony | N/A | **20** | **19** | **19** | **19** |
| *Spodoptera littoralis* (Boisduval) | B164* | DRC | COD | **20** | **19** | **19** | **19** |
| *Spodoptera littoralis* (Boisduval) | B165* | DRC | COD | **20** | **19** | **19** | **19** |
| *Spodoptera littoralis* (Boisduval) | B98* | Egypt | EGY | **20** | **19** | **19** | **19** |
| *Spodoptera littoralis* (Boisduval) | B99* | Egypt | EGY | **20** | **19** | **19** | **19** |
| *Spodoptera littoralis* (Boisduval) | GK118* | Mali | MLI | **20** | **19** | **19** | **19** |
| *Spodoptera littoralis* (Boisduval) | GK119* | Mali | MLI | **20** | **19** | **19** | **19** |
| *Spodoptera littoralis* (Boisduval) | GK120* | Mali | MLI | **20** | **19** | **19** | **19** |
| *Spodoptera littoralis* (Boisduval) | HM756074 | Portugal | PRT | **20** | **19** | **19** | **19** |
| *Spodoptera littoralis* (Boisduval) | LSU35* | Laboratory colony | N/A | **20** | **19** | **19** | **19** |
| *Spodoptera littoralis* (Boisduval) | LSU7* | Laboratory colony | N/A | **20** | **19** | **19** | **19** |
| *Spodoptera litura* (Fabricius) | NIAES:LEPID:179 | Japan | JAP | **21** | **20** | **20** | **20** |
| *Spodoptera litura* (Fabricius) | NIAES:LEPID:180 | Japan | JAP | **21** | **20** | **20** | **20** |
| *Spodoptera litura* (Fabricius) | B113* | Australia | AUS | **21** | **20** | **20** | **20** |
| *Spodoptera litura* (Fabricius) | B114* | Australia | AUS | **21** | **20** | **20** | **20** |
| *Spodoptera litura* (Fabricius) | B15* | Indonesia | IDN | **21** | **20** | **20** | **20** |
| *Spodoptera litura* (Fabricius) | B153* | Indonesia | IDN | **21** | **20** | **20** | **20** |
| *Spodoptera litura* (Fabricius) | B154* | Philippines | PHL | **21** | **20** | **20** | **20** |
| *Spodoptera litura* (Fabricius) | B160* | Indonesia | IDN | **21** | **20** | **20** | **20** |
| *Spodoptera litura* (Fabricius) | GK425** | Indonesia | IDN | **21** | **20** | **20** | **20** |
| *Spodoptera litura* (Fabricius) | GK426** | Indonesia | IDN | **21** | **20** | **20** | **20** |
| *Spodoptera litura* (Fabricius) | LEPIN003-12 | India | IND | **21** | **20** | **20** | **20** |
| *Spodoptera litura* (Fabricius) | GBGL10128-12 | Taiwan | TWN | **21** | **20** | **20** | **20** |
| *Spodoptera litura* (Fabricius) | GBGL10129-12 | Taiwan | TWN | **21** | **20** | **20** | **20** |
| *Spodoptera litura* (Fabricius) | GBGL10130-12 | Taiwan | TWN | **21** | **20** | **20** | **20** |
| *Spodoptera litura* (Fabricius) | GBGL10131-12 | Taiwan | CHN | **21** | **20** | **20** | **20** |
| *Spodoptera litura* (Fabricius) | JSTR00021 0103** | China | CHN | **21** | **20** | **20** | **20** |
| *Spodoptera litura* (Fabricius) | JSTR00026 0201** | China | CHN | **21** | **20** | **20** | **20** |
| *Spodoptera litura* (Fabricius) | JSTR00026 0202** | China | CHN | **21** | **20** | **20** | **20** |
| *Spodoptera litura* (Fabricius) | GBMIN38601-13 | Thailand | THA | **21** | **20** | **20** | **20** |
| *Spodoptera litura* (Fabricius) | GBMIN38650-13 | Bangladesh | BGD | **21** | **20** | **20** | **20** |
| *Spodoptera litura* (Fabricius) | GBMIN38600-13 | Bangladesh | BGD | **21** | **20** | **20** | **20** |
| *Spodoptera litura* (Fabricius) | GBMIN38649-13 | Bangladesh | BGD | **21** | **20** | **20** | **20** |
| *Spodoptera litura* (Fabricius) | GBMIN38599-13 | Bangladesh | BGD | **21** | **20** | **20** | **20** |
| *Spodoptera litura* (Fabricius) | GBMIN38636-13 | unknown | N/A | **21** | **20** | **20** | **20** |
| *Spodoptera litura* (Fabricius) | GBMIN38623-13 | Bangladesh | BGD | **21** | **20** | **20** | **20** |
| *Spodoptera litura* (Fabricius) | GBMIN38573-13 | Pakistan | PAK | **21** | **20** | **20** | **20** |
| *Spodoptera litura* (Fabricius) | GBMIN38622-13 | India | IND | **21** | **20** | **20** | **20** |
| *Spodoptera litura* (Fabricius) | GBMIN38571-13 | India | IND | **21** | **20** | **20** | **20** |
| *Spodoptera litura* (Fabricius) | ANICK311-10 | Australia | AUS | **21** | **20** | **20** | **20** |
| *Spodoptera litura* (Fabricius) | GBMIN22884-13 | South Korea | KOR | **21** | **20** | **20** | **20** |
| *Spodoptera litura* (Fabricius) | GBMIN30175-13 | India | IND | **21** | **20** | **20** | **20** |
| *Spodoptera litura* (Fabricius) | GBMIN30170-13 | India | IND | **21** | **20** | **20** | **20** |
| *Spodoptera litura* (Fabricius) | GBMIN30174-13 | India | IND | **21** | **20** | **20** | **20** |
| *Spodoptera litura* (Fabricius) | GBMIN30169-13 | India | IND | **21** | **20** | **20** | **20** |
| *Spodoptera litura* (Fabricius) | GBMIN30173-13 | India | IND | **21** | **20** | **20** | **20** |
| *Spodoptera litura* (Fabricius) | GBMIN30168-13 | India | IND | **21** | **20** | **20** | **20** |
| *Spodoptera litura* (Fabricius) | GBMIN30172-13 | India | IND | **21** | **20** | **20** | **20** |
| *Spodoptera litura* (Fabricius) | GBMIN30167-13 | India | IND | **21** | **20** | **20** | **20** |
| *Spodoptera litura* (Fabricius) | GBMIN22009-13 | China | CHN | **21** | **20** | **20** | **20** |
| *Spodoptera litura* (Fabricius) | GBGL12681-13 | Philippines | PHL | **21** | **20** | **20** | **20** |
| *Spodoptera litura* (Fabricius) | LEPIN014-13 | India | IND | **21** | **20** | **20** | **20** |
| *Spodoptera litura* (Fabricius) | LEPIN015-13 | India | IND | **21** | **20** | **20** | **20** |
| *Spodoptera litura* (Fabricius) | LEPIN044-15 | India | IND | **21** | **20** | **20** | **20** |
| *Spodoptera praefica* (Grote) | B105* | USA - California | USA | **22** | **21** | **21** | **21** |
| *Spodoptera praefica* (Grote) | B30* | USA - California | USA | **22** | **21** | **21** | **21** |
| *Spodoptera praefica* (Grote) | B5* | USA - California | USA | **22** | **21** | **21** | **21** |
| *Spodoptera praefica* (Grote) | LSU32* | USA - California | USA | **22** | **21** | **21** | **21** |
| *Spodoptera pulchella* (H.-Schäffer) | GBGL10113-12 | USA - Florida | USA | **23** | **22** | **22** | **22** |
| *Spodoptera pulchella* (H.-Schäffer) | GBGL10114-12 | USA - Florida | USA | **23** | **22** | **22** | **22** |
| *Spodoptera androgea* (Stoll) | B1* | Guadeloupe | GLP | **24** | **23** | **23** | **23** |
| *Spodoptera androgea* (Stoll) | MNHN20** | Dominican Republic | DOM | **24** | **23** | **23** | **23** |
| *Spodoptera androgea* (Stoll) | MNHN21** | Panama | PAN | **24** | **23** | **23** | **23** |
| *Spodoptera androgea* (Stoll) | MNHN22** | Ecuador | ECU | **24** | **23** | **23** | **23** |
| *Spodoptera androgea* (Stoll) | MNHN23** | Martinique | MTQ | **24** | **23** | **23** | **23** |
| *Spodoptera androgea* (Stoll) | MNHN24** | Peru | PER | **24** | **23** | **23** | **23** |
| *Spodoptera androgea* (Stoll) | MNHN66** | Dominican Republic | DOM | **24** | **23** | **23** | **23** |
| *Spodoptera androgea* (Stoll) | MHAUB900-05 | Costa Rica | CRI | **24** | **23** | **23** | **23** |
| *Spodoptera androgea* (Stoll) | MHAUB901-05 | Costa Rica | CRI | **24** | **23** | **23** | **23** |
| *Spodoptera androgea* (Stoll) | MHAUB903-05 | Costa Rica | CRI | **24** | **23** | **23** | **23** |
| *Spodoptera androgea* (Stoll) | MHAUB907-05 | Costa Rica | CRI | **24** | **23** | **23** | **23** |
| *Spodoptera androgea* (Stoll) | MHAUB902-05 | Costa Rica | CRI | **24** | **23** | **23** | **23** |
| *Spodoptera androgea* (Stoll) | MHAUB905-05 | Costa Rica | CRI | **24** | **23** | **23** | **23** |
| *Spodoptera androgea* (Stoll) | MHAUB904-05 | Costa Rica | CRI | **24** | **23** | **23** | **23** |
| *Spodoptera androgea* (Stoll) | MHAUB906-05 | Costa Rica | CRI | **24** | **23** | **23** | **23** |
| *Spodoptera androgea* (Stoll) | MHAUB908-05 | Costa Rica | CRI | **24** | **23** | **23** | **23** |
| *Spodoptera androgea* (Stoll) | MHAUB909-05 | Costa Rica | CRI | **24** | **23** | **23** | **23** |
| *Spodoptera androgea* (Stoll) | MHAUF370-06 | Costa Rica | CRI | **24** | **23** | **23** | **23** |
| *Spodoptera androgea* (Stoll) | MHMXV046-08 | Costa Rica | CRI | **24** | **23** | **23** | **23** |
| *Spodoptera androgea* (Stoll) | BLPDA435-09 | Costa Rica | CRI | **24** | **23** | **23** | **23** |
| *Spodoptera androgea* (Stoll) | BLPDA718-09 | Costa Rica | CRI | **24** | **23** | **23** | **23** |
| *Spodoptera androgea* (Stoll) | BLPDC921-09 | Costa Rica | CRI | **24** | **23** | **23** | **23** |
| *Spodoptera androgea* (Stoll) | BLPDD477-09 | Costa Rica | CRI | **24** | **23** | **23** | **23** |
| *Spodoptera androgea* (Stoll) | BLPDD478-09 | Costa Rica | CRI | **24** | **23** | **23** | **23** |
| *Spodoptera androgea* (Stoll) | BLPDD685-09 | Costa Rica | CRI | **24** | **23** | **23** | **23** |
| *Spodoptera androgea* (Stoll) | BLPDD686-09 | Costa Rica | CRI | **24** | **23** | **23** | **23** |
| *Spodoptera androgea* (Stoll) | BLPCI618-08 | Costa Rica | CRI | **24** | **23** | **23** | **23** |
| *Spodoptera androgea* (Stoll) | BLPCJ205-08 | Costa Rica | CRI | **24** | **23** | **23** | **23** |
| *Spodoptera androgea* (Stoll) | BLPCL009-08 | Costa Rica | CRI | **24** | **23** | **23** | **23** |
| *Spodoptera androgea* (Stoll) | BLPCL010-08 | Costa Rica | CRI | **24** | **23** | **23** | **23** |
| *Spodoptera androgea* (Stoll) | BLPCO066-08 | Costa Rica | CRI | **24** | **23** | **23** | **23** |
| *Spodoptera androgea* (Stoll) | BLPCC488-08 | Costa Rica | CRI | **24** | **23** | **23** | **23** |
| *Spodoptera androgea* (Stoll) | BLPCD471-08 | Costa Rica | CRI | **24** | **23** | **23** | **23** |
| *Spodoptera androgea* (Stoll) | BLPCD475-08 | Costa Rica | CRI | **24** | **23** | **23** | **23** |
| *Spodoptera androgea* (Stoll) | BLPCF454-08 | Costa Rica | CRI | **24** | **23** | **23** | **23** |
| *Spodoptera androgea* (Stoll) | BLPCF455-08 | Costa Rica | CRI | **24** | **23** | **23** | **23** |
| *Spodoptera androgea* (Stoll) | BLPCF456-08 | Costa Rica | CRI | **24** | **23** | **23** | **23** |
| *Spodoptera androgea* (Stoll) | BLPBF046-07 | Costa Rica | CRI | **24** | **23** | **23** | **23** |
| *Spodoptera androgea* (Stoll) | BLPBF047-07 | Costa Rica | CRI | **24** | **23** | **23** | **23** |
| *Spodoptera androgea* (Stoll) | BLPBF048-07 | Costa Rica | CRI | **24** | **23** | **23** | **23** |
| *Spodoptera androgea* (Stoll) | BLPBF049-07 | Costa Rica | CRI | **24** | **23** | **23** | **23** |
| *Spodoptera androgea* (Stoll) | BLPCC003-08 | Costa Rica | CRI | **24** | **23** | **23** | **23** |
| *Spodoptera androgea* (Stoll) | BLPCC004-08 | Costa Rica | CRI | **24** | **23** | **23** | **23** |
| *Spodoptera androgea* (Stoll) | BLPAE060-06 | Costa Rica | CRI | **24** | **23** | **23** | **23** |
| *Spodoptera androgea* (Stoll) | BLPAE061-06 | Costa Rica | CRI | **24** | **23** | **23** | **23** |
| *Spodoptera androgea* (Stoll) | BLPAG449-07 | Costa Rica | CRI | **24** | **23** | **23** | **23** |
| *Spodoptera androgea* (Stoll) | BLPBB638-07 | Costa Rica | CRI | **24** | **23** | **23** | **23** |
| *Spodoptera androgea* (Stoll) | BLPBC143-07 | Costa Rica | CRI | **24** | **23** | **23** | **23** |
| *Spodoptera androgea* (Stoll) | BLPBD224-07 | Costa Rica | CRI | **24** | **23** | **23** | **23** |
| *Spodoptera androgea* (Stoll) | BLPAA821-06 | Costa Rica | CRI | **24** | **23** | **23** | **23** |
| *Spodoptera androgea* (Stoll) | BLPAA925-06 | Costa Rica | CRI | **24** | **23** | **23** | **23** |
| *Spodoptera androgea* (Stoll) | BLPAB812-06 | Costa Rica | CRI | **24** | **23** | **23** | **23** |
| *Spodoptera androgea* (Stoll) | BLPAB894-06 | Costa Rica | CRI | **24** | **23** | **23** | **23** |
| *Spodoptera androgea* (Stoll) | BLPAC245-06 | Costa Rica | CRI | **24** | **23** | **23** | **23** |
| *Spodoptera androgea* (Stoll) | BLPAC246-06 | Costa Rica | CRI | **24** | **23** | **23** | **23** |
| *Spodoptera androgea* (Stoll) | BLPAC327-06.COI | Costa Rica | CRI | **24** | **23** | **23** | **23** |
| *Spodoptera androgea* (Stoll) | BLPAC726-06 | Costa Rica | CRI | **24** | **23** | **23** | **23** |
| *Spodoptera androgea* (Stoll) | MHMXA727-06 | Costa Rica | CRI | **24** | **23** | **23** | **23** |
| *Spodoptera androgea* (Stoll) | MHMXA730-06 | Costa Rica | CRI | **24** | **23** | **23** | **23** |
| *Spodoptera androgea* (Stoll) | MHMXD719-06 | Costa Rica | CRI | **24** | **23** | **23** | **23** |
| *Spodoptera ornithogalli* (Guenée) | B106* | USA | USA | **25** | **24** | **24** | **24** |
| *Spodoptera ornithogalli* (Guenée) | B121* | USA - Georgia | USA | **25** | **24** | **24** | **24** |
| *Spodoptera ornithogalli* (Guenée) | B146* | French Guiana | GUF | **25** | **24** | **24** | **24** |
| *Spodoptera ornithogalli* (Guenée) | B28* | USA - Louisiana | USA | **25** | **24** | **24** | **24** |
| *Spodoptera ornithogalli* (Guenée) | B4* | USA - Louisiana | USA | **25** | **24** | **24** | **24** |
| *Spodoptera ornithogalli* (Guenée) | B54* | USA - Louisiana | USA | **25** | **24** | **24** | **24** |
| *Spodoptera ornithogalli* (Guenée) | LSU34* | USA - Louisiana | USA | **25** | **24** | **24** | **24** |
| *Spodoptera ornithogalli* (Guenée) | LSU8* | USA | USA | **25** | **24** | **24** | **24** |
| *Spodoptera ornithogalli* (Guenée) | MNHN41** | Guatemala | GTM | **25** | **24** | **24** | **24** |
| *Spodoptera ornithogalli* (Guenée) | MNHN69** | Dominican Repubic | DOM | **25** | **24** | **24** | **24** |
| *Spodoptera ornithogalli* (Guenée) | GBGL5878-09 | USA - Maryland | USA | **25** | **24** | **24** | **24** |
| *Spodoptera ornithogalli* (Guenée) | LOT327-04 | USA - Tennessee | USA | **25** | **24** | **24** | **24** |
| *Spodoptera ornithogalli* (Guenée) | LOT326-04 | USA - Tennessee | USA | **25** | **24** | **24** | **24** |
| *Spodoptera ornithogalli* (Guenée) | LGSM428-04 | USA - Tennessee | USA | **25** | **24** | **24** | **24** |
| *Spodoptera ornithogalli* (Guenée) | LGSM429-04 | USA - Tennessee | USA | **25** | **24** | **24** | **24** |
| *Spodoptera ornithogalli* (Guenée) | XAB643-04 | Canada - Ontario | CAN | **25** | **24** | **24** | **24** |
| *Spodoptera ornithogalli* (Guenée) | MJMSL004-10 | USA - Massachusetts | USA | **25** | **24** | **24** | **24** |
| *Spodoptera ornithogalli* (Guenée) | GBGL3732-06 | USA | USA | **25** | **24** | **24** | **24** |
| *Spodoptera marima* (Schaus) | B145* | French Guiana | GUF | **25** | **24** | **24** | **24** |
| *Spodoptera marima* (Schaus) | MNHN39** | Venezuela | VEN | **25** | **24** | **24** | **24** |
| *Spodoptera marima* (Schaus) | MNHN40** | Venezuela | VEN | **25** | **24** | **24** | **24** |
| *Spodoptera marima* (Schaus) | MNHN58** | French Guiana | GUF | **25** | **24** | **24** | **24** |
| *Spodoptera dolichos* (Fabricius) | B104* | Guadeloupe | GLP | **26** | **25** | **25** | **25** |
| *Spodoptera dolichos* (Fabricius) | B122* | USA - Georgia | USA | **26** | **25** | **25** | **25** |
| *Spodoptera dolichos* (Fabricius) | B35* | French Guiana | GUF | **26** | **25** | **25** | **25** |
| *Spodoptera dolichos* (Fabricius) | B37* | Guadeloupe | GLP | **26** | **25** | **25** | **25** |
| *Spodoptera dolichos* (Fabricius) | LSU14* | French Guiana | GUF | **26** | **25** | **25** | **25** |
| *Spodoptera dolichos* (Fabricius) | LSU27* | USA - Florida | USA | **26** | **25** | **25** | **25** |
| *Spodoptera dolichos* (Fabricius) | LSU3* | Guadeloupe | GLP | **26** | **25** | **25** | **25** |
| *Spodoptera dolichos* (Fabricius) | MNHN34** | Dominican Republic | DOM | **26** | **25** | **25** | **25** |
| *Spodoptera dolichos* (Fabricius) | MNHN35** | Venezuela | VEN | **26** | **25** | **25** | **25** |
| *Spodoptera dolichos* (Fabricius) | MNHN36** | French Guiana | GUF | **26** | **25** | **25** | **25** |
| *Spodoptera dolichos* (Fabricius) | MNHN37** | Jamaica | JAM | **26** | **25** | **25** | **25** |
| *Spodoptera dolichos* (Fabricius) | MNHN38** | Martinique | MTQ | **26** | **25** | **25** | **25** |
| *Spodoptera dolichos* (Fabricius) | MNHN65** | Peru | PER | **26** | **25** | **25** | **25** |
| *Spodoptera dolichos* (Fabricius) | MNHN7** | Martinique | MTQ | **26** | **25** | **25** | **25** |
| *Spodoptera dolichos* (Fabricius) | MNHN8** | Martinique | MTQ | **26** | **25** | **25** | **25** |
| *Spodoptera dolichos* (Fabricius) | MHAUB912-05 | Costa Rica | CRI | **26** | **25** | **25** | **25** |
| *Spodoptera dolichos* (Fabricius) | MHAUB911-05 | Costa Rica | CRI | **26** | **25** | **25** | **25** |
| *Spodoptera dolichos* (Fabricius) | MHAUB910-05 | Costa Rica | CRI | **26** | **25** | **25** | **25** |
| *Spodoptera dolichos* (Fabricius) | MHAUB914-05 | Costa Rica | CRI | **26** | **25** | **25** | **25** |
| *Spodoptera dolichos* (Fabricius) | MHAUB915-05 | Costa Rica | CRI | **26** | **25** | **25** | **25** |
| *Spodoptera dolichos* (Fabricius) | MHAUB913-05 | Costa Rica | CRI | **26** | **25** | **25** | **25** |
| *Spodoptera dolichos* (Fabricius) | MHAUF369-06 | Costa Rica | CRI | **26** | **25** | **25** | **25** |
| *Spodoptera dolichos* (Fabricius) | MHAUC210-06 | Costa Rica | CRI | **26** | **25** | **25** | **25** |
| *Spodoptera dolichos* (Fabricius) | GBGL10124-12 | USA - Florida | USA | **26** | **25** | **25** | **25** |
| *Spodoptera dolichos* (Fabricius) | GBGL10125-12 | USA - Florida | USA | **26** | **25** | **25** | **25** |
| *Spodoptera dolichos* (Fabricius) | GBGL10126-12 | USA - Florida | USA | **26** | **25** | **25** | **25** |
| *Spodoptera dolichos* (Fabricius) | GBGL10127-12 | USA - Florida | USA | **26** | **25** | **25** | **25** |
| *Spodoptera dolichos* (Fabricius) | B2* | French Guiana | GUF | **26** | **25** | **25** | **25** |
| *Spodoptera dolichos* (Fabricius) | LSU29* | French Guiana | GUF | **26** | **25** | **25** | **25** |
| *Spodoptera dolichos* (Fabricius) | LEMMZ124-10 | Brazil | BRA | **26** | **25** | **25** | **25** |
| *Spodoptera dolichos* (Fabricius) | BLPAG055-07 | Costa Rica | CRI | **26** | **25** | **25** | **25** |
| *Spodoptera dolichos* (Fabricius) | MHMXQ639-08 | Costa Rica | CRI | **26** | **25** | **25** | **25** |
| *Spodoptera dolichos* (Fabricius) | BLPCP113-08 | Costa Rica | CRI | **26** | **25** | **25** | **25** |
| *Spodoptera dolichos* (Fabricius) | BLPDB841-09 | Costa Rica | CRI | **26** | **25** | **25** | **25** |
| *Spodoptera dolichos* (Fabricius) | BLPDC205-09 | Costa Rica | CRI | **26** | **25** | **25** | **25** |
| *Spodoptera dolichos* (Fabricius) | BLPCI619-08 | Costa Rica | CRI | **26** | **25** | **25** | **25** |
| *Spodoptera dolichos* (Fabricius) | BLPCJ098-08 | Costa Rica | CRI | **26** | **25** | **25** | **25** |
| *Spodoptera dolichos* (Fabricius) | BLPCK133-08 | Costa Rica | CRI | **26** | **25** | **25** | **25** |
| *Spodoptera dolichos* (Fabricius) | BLPCO135-08 | Costa Rica | CRI | **26** | **25** | **25** | **25** |
| *Spodoptera dolichos* (Fabricius) | BLPCD470-08 | Costa Rica | CRI | **26** | **25** | **25** | **25** |
| *Spodoptera dolichos* (Fabricius) | BLPCD472-08 | Costa Rica | CRI | **26** | **25** | **25** | **25** |
| *Spodoptera dolichos* (Fabricius) | BLPCD473-08 | Costa Rica | CRI | **26** | **25** | **25** | **25** |
| *Spodoptera dolichos* (Fabricius) | BLPCD476-08 | Costa Rica | CRI | **26** | **25** | **25** | **25** |
| *Spodoptera dolichos* (Fabricius) | BLPCD478-08 | Costa Rica | CRI | **26** | **25** | **25** | **25** |
| *Spodoptera dolichos* (Fabricius) | BLPCH049-08 | Costa Rica | CRI | **26** | **25** | **25** | **25** |
| *Spodoptera dolichos* (Fabricius) | BLPBG173-07 | Costa Rica | CRI | **26** | **25** | **25** | **25** |
| *Spodoptera dolichos* (Fabricius) | BLPBH662-07 | Costa Rica | CRI | **26** | **25** | **25** | **25** |
| *Spodoptera dolichos* (Fabricius) | BLPBH664-07 | Costa Rica | CRI | **26** | **25** | **25** | **25** |
| *Spodoptera dolichos* (Fabricius) | BLPAF454-07 | Costa Rica | CRI | **26** | **25** | **25** | **25** |
| *Spodoptera dolichos* (Fabricius) | BLPAF455-07 | Costa Rica | CRI | **26** | **25** | **25** | **25** |
| *Spodoptera dolichos* (Fabricius) | BLPAF456-07 | Costa Rica | CRI | **26** | **25** | **25** | **25** |
| *Spodoptera dolichos* (Fabricius) | BLPAG030-07 | Costa Rica | CRI | **26** | **25** | **25** | **25** |
| *Spodoptera dolichos* (Fabricius) | BLPAH330-07 | Costa Rica | CRI | **26** | **25** | **25** | **25** |
| *Spodoptera dolichos* (Fabricius) | BLPAH331-07 | Costa Rica | CRI | **26** | **25** | **25** | **25** |
| *Spodoptera dolichos* (Fabricius) | BLPBA273-07 | Costa Rica | CRI | **26** | **25** | **25** | **25** |
| *Spodoptera dolichos* (Fabricius) | BLPAA820-06 | Costa Rica | CRI | **26** | **25** | **25** | **25** |
| *Spodoptera dolichos* (Fabricius) | BLPAA926-06 | Costa Rica | CRI | **26** | **25** | **25** | **25** |
| *Spodoptera dolichos* (Fabricius) | BLPAA927-06 | Costa Rica | CRI | **26** | **25** | **25** | **25** |
| *Spodoptera dolichos* (Fabricius) | BLPAB037-06 | Costa Rica | CRI | **26** | **25** | **25** | **25** |
| *Spodoptera dolichos* (Fabricius) | BLPAB238-06 | Costa Rica | CRI | **26** | **25** | **25** | **25** |
| *Spodoptera dolichos* (Fabricius) | BLPAB239-06 | Costa Rica | CRI | **26** | **25** | **25** | **25** |
| *Spodoptera dolichos* (Fabricius) | BLPAB275-06 | Costa Rica | CRI | **26** | **25** | **25** | **25** |
| *Spodoptera dolichos* (Fabricius) | BLPAB276-06 | Costa Rica | CRI | **26** | **25** | **25** | **25** |
| *Spodoptera dolichos* (Fabricius) | BLPAC328-06 | Costa Rica | CRI | **26** | **25** | **25** | **25** |
| *Spodoptera dolichos* (Fabricius) | BLPAD217-06 | Costa Rica | CRI | **26** | **25** | **25** | **25** |
| *Spodoptera dolichos* (Fabricius) | MHMXO341-08 | Costa Rica | CRI | **26** | **25** | **25** | **25** |
| *Spodoptera dolichos* (Fabricius) | MHMXA733-06 | Costa Rica | CRI | **26** | **25** | **25** | **25** |
| *Spodoptera cosmiodes* (Walker) | B100* | French Guiana | GUF | **27** | **26** | **26** | **26** |
| *Spodoptera cosmiodes* (Walker) | B150* | Brazil | BRA | **27** | **26** | **26** | **26** |
| *Spodoptera cosmiodes* (Walker) | B151* | Brazil | BRA | **27** | **26** | **26** | **26** |
| *Spodoptera cosmiodes* (Walker) | B166* | Brazil | BRA | **27** | **26** | **26** | **26** |
| *Spodoptera cosmiodes* (Walker) | B19* | French Guiana | GUF | **27** | **26** | **26** | **26** |
| *Spodoptera cosmiodes* (Walker) | LEMMZ123-10 | Brazil | BRA | **27** | **26** | **26** | **26** |
| *Spodoptera cosmiodes* (Walker) | LEMMZ126-10 | Brazil | BRA | **27** | **26** | **26** | **26** |
| *Spodoptera cosmiodes* (Walker) | GBGL12629-13 | Brazil | BRA | **27** | **26** | **26** | **26** |
| *Spodoptera cosmiodes* (Walker) | GBGL12628-13 | Brazil | BRA | **27** | **26** | **26** | **26** |
| *Spodoptera cosmiodes* (Walker) | GBGL12627-13 | Brazil | BRA | **27** | **26** | **26** | **26** |
| *Spodoptera cosmiodes* (Walker) | GBGL12626-13 | Brazil | BRA | **27** | **26** | **26** | **26** |
| *Spodoptera cosmiodes* (Walker) | GBGL12625-13 | Brazil | BRA | **27** | **26** | **26** | **26** |
| *Spodoptera cosmiodes* (Walker) | GBGL12624-13 | Brazil | BRA | **27** | **26** | **26** | **26** |
| *Spodoptera cosmiodes* (Walker) | GBGL12623-13 | Brazil | BRA | **27** | **26** | **26** | **26** |
| *Spodoptera cosmiodes* (Walker) | GBGL12622-13 | Brazil | BRA | **27** | **26** | **26** | **26** |
| *Spodoptera cosmiodes* (Walker) | GBGL12621-13 | Brazil | BRA | **27** | **26** | **26** | **26** |
| *Spodoptera cosmiodes* (Walker) | GBGL12619-13 | Brazil | BRA | **27** | **26** | **26** | **26** |
| *Spodoptera cosmiodes* (Walker) | GBGL12618-13 | Brazil | BRA | **27** | **26** | **26** | **26** |
| *Spodoptera cosmiodes* (Walker) | GBGL12617-13 | Brazil | BRA | **27** | **26** | **26** | **26** |
| *Spodoptera cosmiodes* (Walker) | GBGL12616-13 | Brazil | BRA | **27** | **26** | **26** | **26** |
| *Spodoptera cosmiodes* (Walker) | GBGL12615-13 | Brazil | BRA | **27** | **26** | **26** | **26** |
| *Spodoptera cosmiodes* (Walker) | GBGL12614-13 | Brazil | BRA | **27** | **26** | **26** | **26** |
| *Spodoptera cosmiodes* (Walker) | GBGL12613-13 | Brazil | BRA | **27** | **26** | **26** | **26** |
| *Spodoptera cosmiodes* (Walker) | GBGL12612-13 | Brazil | BRA | **27** | **26** | **26** | **26** |
| *Spodoptera cosmiodes* (Walker) | GBGL12611-13 | Brazil | BRA | **27** | **26** | **26** | **26** |
| *Spodoptera cosmiodes* (Walker) | GBGL12610-13 | Brazil | BRA | **27** | **26** | **26** | **26** |
| *Spodoptera cosmiodes* (Walker) | GBGL12609-13 | Brazil | BRA | **27** | **26** | **26** | **26** |
| *Spodoptera cosmiodes* (Walker) | GBGL12608-13 | Brazil | BRA | **27** | **26** | **26** | **26** |
| *Spodoptera cosmiodes* (Walker) | GBGL12607-13 | Brazil | BRA | **27** | **26** | **26** | **26** |
| *Spodoptera cosmiodes* (Walker) | GBGL12606-13 | Brazil | BRA | **27** | **26** | **26** | **26** |
| *Spodoptera cosmiodes* (Walker) | GBGL12605-13 | Brazil | BRA | **27** | **26** | **26** | **26** |
| *Spodoptera cosmiodes* (Walker) | GBGL12604-13 | Brazil | BRA | **27** | **26** | **26** | **26** |
| *Spodoptera cosmiodes* (Walker) | GBGL12603-13 | Brazil | BRA | **27** | **26** | **26** | **26** |
| *Spodoptera cosmiodes* (Walker) | GBGL12602-13 | Brazil | BRA | **27** | **26** | **26** | **26** |
| *Spodoptera cosmiodes* (Walker) | GBGL12601-13 | Brazil | BRA | **27** | **26** | **26** | **26** |
| *Spodoptera descoinsi* L.-C. & Silvain | LSU30* | French Guiana | GUF | **27** | **26** | **26** | **26** |
| *Spodoptera descoinsi* L.-C. & Silvain | B32* | French Guiana | GUF | **27** | **26** | **26** | **26** |
| *Spodoptera descoinsi* L.-C. & Silvain | B102* | French Guiana | GUF | **28** | **27** | **27** | **27** |
| *Spodoptera descoinsi* L.-C. & Silvain | B157* | French Guiana | GUF | **28** | **27** | **27** | **27** |
| *Spodoptera descoinsi* L.-C. & Silvain | B38* | French Guiana | GUF | **28** | **27** | **27** | **27** |
| *Spodoptera descoinsi* L.-C. & Silvain | LSU31* | French Guiana | GUF | **28** | **27** | **27** | **27** |
| *Spodoptera descoinsi* L.-C. & Silvain | MNHN43** | French Guiana | GUF | **28** | **27** | **27** | **27** |
| *Spodoptera descoinsi* L.-C. & Silvain | MNHN44** | French Guiana | GUF | **28** | **27** | **27** | **27** |
| *Spodoptera descoinsi* L.-C. & Silvain | MNHN45** | French Guiana | GUF | **28** | **27** | **27** | **27** |
| *Spodoptera evanida* Schaus | B155* | French Guiana | GUF | **29** | **28** | **27** | **27** |
| *Spodoptera evanida* Schaus | B156* | French Guiana | GUF | **29** | **28** | **27** | **27** |
| *Spodoptera evanida* Schaus | MNHN52** | French Guiana | GUF | **29** | **28** | **27** | **27** |
| *Spodoptera evanida* Schaus | MNHN53** | French Guiana | GUF | **29** | **28** | **27** | **27** |
| *Spodoptera evanida* Schaus | MNHN54** | French Guiana | GUF | **29** | **28** | **27** | **27** |
| *Spodoptera evanida* Schaus | MNHN55** | French Guiana | GUF | **29** | **28** | **27** | **27** |
| *Spodoptera evanida* Schaus | MNHN60** | French Guiana | GUF | **29** | **28** | **27** | **27** |
| *Spodoptera evanida* Schaus | MNHN61** | French Guiana | GUF | **29** | **28** | **27** | **27** |
| *Spodoptera evanida* Schaus | MNHN71** | Peru | PER | **29** | **28** | **27** | **27** |
| *Spodoptera evanida* Schaus | MNHN72** | French Guiana | GUF | **29** | **28** | **27** | **27** |
| *Spodoptera latifascia* (Walker) | MHAUB922-05 | Costa Rica | CRI | **30** | **29** | **28** | **28** |
| *Spodoptera latifascia* (Walker) | MHAUB924-05 | Costa Rica | CRI | **30** | **29** | **28** | **28** |
| *Spodoptera latifascia* (Walker) | MHAUB925-05 | Costa Rica | CRI | **30** | **29** | **28** | **28** |
| *Spodoptera latifascia* (Walker) | MHAUB918-05 | Costa Rica | CRI | **30** | **29** | **28** | **28** |
| *Spodoptera latifascia* (Walker) | MHAUB921-05 | Costa Rica | CRI | **30** | **29** | **28** | **28** |
| *Spodoptera latifascia* (Walker) | MHAUB919-05 | Costa Rica | CRI | **30** | **29** | **28** | **28** |
| *Spodoptera latifascia* (Walker) | MHAUB920-05 | Costa Rica | CRI | **30** | **29** | **28** | **28** |
| *Spodoptera latifascia* (Walker) | MHAUB916-05 | Costa Rica | CRI | **30** | **29** | **28** | **28** |
| *Spodoptera latifascia* (Walker) | MHAUB917-05 | Costa Rica | CRI | **30** | **29** | **28** | **28** |
| *Spodoptera latifascia* (Walker) | MHAUB923-05 | Costa Rica | CRI | **30** | **29** | **28** | **28** |
| *Spodoptera latifascia* (Walker) | MHAUC211-06 | Costa Rica | CRI | **30** | **29** | **28** | **28** |
| *Spodoptera latifascia* (Walker) | MHMYC2258-09 | Costa Rica | CRI | **30** | **29** | **28** | **28** |
| *Spodoptera latifascia* (Walker) | MHMXU488-08 | Costa Rica | CRI | **30** | **29** | **28** | **28** |
| *Spodoptera latifascia* (Walker) | BLPDU281-11 | Costa Rica | CRI | **30** | **29** | **28** | **28** |
| *Spodoptera latifascia* (Walker) | BLPDU282-11 | Costa Rica | CRI | **30** | **29** | **28** | **28** |
| *Spodoptera latifascia* (Walker) | BLPDU283-11 | Costa Rica | CRI | **30** | **29** | **28** | **28** |
| *Spodoptera latifascia* (Walker) | BLPDU284-11 | Costa Rica | CRI | **30** | **29** | **28** | **28** |
| *Spodoptera latifascia* (Walker) | BLPDU884-11 | Costa Rica | CRI | **30** | **29** | **28** | **28** |
| *Spodoptera latifascia* (Walker) | BLPDV1050-11 | Costa Rica | CRI | **30** | **29** | **28** | **28** |
| *Spodoptera latifascia* (Walker) | BLPDV1051-11 | Costa Rica | CRI | **30** | **29** | **28** | **28** |
| *Spodoptera latifascia* (Walker) | BLPDV1056-11 | Costa Rica | CRI | **30** | **29** | **28** | **28** |
| *Spodoptera latifascia* (Walker) | BLPDY798-11 | Costa Rica | CRI | **30** | **29** | **28** | **28** |
| *Spodoptera latifascia* (Walker) | BLPDB355-09 | Costa Rica | CRI | **30** | **29** | **28** | **28** |
| *Spodoptera latifascia* (Walker) | BLPDE113-09 | Costa Rica | CRI | **30** | **29** | **28** | **28** |
| *Spodoptera latifascia* (Walker) | BLPDE114-09 | Costa Rica | CRI | **30** | **29** | **28** | **28** |
| *Spodoptera latifascia* (Walker) | BLPDE115-09 | Costa Rica | CRI | **30** | **29** | **28** | **28** |
| *Spodoptera latifascia* (Walker) | BLPCI616-08 | Costa Rica | CRI | **30** | **29** | **28** | **28** |
| *Spodoptera latifascia* (Walker) | BLPCI617-08 | Costa Rica | CRI | **30** | **29** | **28** | **28** |
| *Spodoptera latifascia* (Walker) | BLPCJ553-08 | Costa Rica | CRI | **30** | **29** | **28** | **28** |
| *Spodoptera latifascia* (Walker) | BLPCJ554-08 | Costa Rica | CRI | **30** | **29** | **28** | **28** |
| *Spodoptera latifascia* (Walker) | BLPCJ555-08 | Costa Rica | CRI | **30** | **29** | **28** | **28** |
| *Spodoptera latifascia* (Walker) | BLPCK290-08 | Costa Rica | CRI | **30** | **29** | **28** | **28** |
| *Spodoptera latifascia* (Walker) | BLPCL008-08 | Costa Rica | CRI | **30** | **29** | **28** | **28** |
| *Spodoptera latifascia* (Walker) | BLPCD474-08 | Costa Rica | CRI | **30** | **29** | **28** | **28** |
| *Spodoptera latifascia* (Walker) | BLPCD477-08 | Costa Rica | CRI | **30** | **29** | **28** | **28** |
| *Spodoptera latifascia* (Walker) | BLPCD479-08 | Costa Rica | CRI | **30** | **29** | **28** | **28** |
| *Spodoptera latifascia* (Walker) | BLPCD480-08 | Costa Rica | CRI | **30** | **29** | **28** | **28** |
| *Spodoptera latifascia* (Walker) | BLPCH656-08 | Costa Rica | CRI | **30** | **29** | **28** | **28** |
| *Spodoptera latifascia* (Walker) | BLPCI137-08 | Costa Rica | CRI | **30** | **29** | **28** | **28** |
| *Spodoptera latifascia* (Walker) | BLPBF915-07 | Costa Rica | CRI | **30** | **29** | **28** | **28** |
| *Spodoptera latifascia* (Walker) | BLPBH663-07 | Costa Rica | CRI | **30** | **29** | **28** | **28** |
| *Spodoptera latifascia* (Walker) | BLPCA159-08 | Costa Rica | CRI | **30** | **29** | **28** | **28** |
| *Spodoptera latifascia* (Walker) | BLPCA207-08 | Costa Rica | CRI | **30** | **29** | **28** | **28** |
| *Spodoptera latifascia* (Walker) | BLPCA208-08 | Costa Rica | CRI | **30** | **29** | **28** | **28** |
| *Spodoptera latifascia* (Walker) | BLPCA519-08 | Costa Rica | CRI | **30** | **29** | **28** | **28** |
| *Spodoptera latifascia* (Walker) | BLPCA520-08 | Costa Rica | CRI | **30** | **29** | **28** | **28** |
| *Spodoptera latifascia* (Walker) | BLPCA521-08 | Costa Rica | CRI | **30** | **29** | **28** | **28** |
| *Spodoptera latifascia* (Walker) | BLPCA522-08 | Costa Rica | CRI | **30** | **29** | **28** | **28** |
| *Spodoptera latifascia* (Walker) | BLPCA523-08 | Costa Rica | CRI | **30** | **29** | **28** | **28** |
| *Spodoptera latifascia* (Walker) | BLPCA525-08 | Costa Rica | CRI | **30** | **29** | **28** | **28** |
| *Spodoptera latifascia* (Walker) | BLPAF457-07 | Costa Rica | CRI | **30** | **29** | **28** | **28** |
| *Spodoptera latifascia* (Walker) | BLPAG029-07 | Costa Rica | CRI | **30** | **29** | **28** | **28** |
| *Spodoptera latifascia* (Walker) | BLPAG031-07 | Costa Rica | CRI | **30** | **29** | **28** | **28** |
| *Spodoptera latifascia* (Walker) | BLPBA005-07 | Costa Rica | CRI | **30** | **29** | **28** | **28** |
| *Spodoptera latifascia* (Walker) | BLPAA382-06 | Costa Rica | CRI | **30** | **29** | **28** | **28** |
| *Spodoptera latifascia* (Walker) | BLPAA386-06 | Costa Rica | CRI | **30** | **29** | **28** | **28** |
| *Spodoptera latifascia* (Walker) | BLPAA394-06 | Costa Rica | CRI | **30** | **29** | **28** | **28** |
| *Spodoptera latifascia* (Walker) | BLPAA463-06 | Costa Rica | CRI | **30** | **29** | **28** | **28** |
| *Spodoptera latifascia* (Walker) | BLPAA707-06 | Costa Rica | CRI | **30** | **29** | **28** | **28** |
| *Spodoptera latifascia* (Walker) | BLPAA730-06 | Costa Rica | CRI | **30** | **29** | **28** | **28** |
| *Spodoptera latifascia* (Walker) | BLPAA922-06 | Costa Rica | CRI | **30** | **29** | **28** | **28** |
| *Spodoptera latifascia* (Walker) | BLPAB893-06 | Costa Rica | CRI | **30** | **29** | **28** | **28** |
| *Spodoptera latifascia* (Walker) | BLPAC244-06 | Costa Rica | CRI | **30** | **29** | **28** | **28** |
| *Spodoptera latifascia* (Walker) | BLPAC247-06 | Costa Rica | CRI | **30** | **29** | **28** | **28** |
| *Spodoptera latifascia* (Walker) | BLPAC723-06 | Costa Rica | CRI | **30** | **29** | **28** | **28** |
| *Spodoptera latifascia* (Walker) | BLPAC725-06 | Costa Rica | CRI | **30** | **29** | **28** | **28** |
| *Spodoptera latifascia* (Walker) | BLPAD014-06 | Costa Rica | CRI | **30** | **29** | **28** | **28** |
| *Spodoptera latifascia* (Walker) | MHMXA728-06 | Costa Rica | CRI | **30** | **29** | **28** | **28** |
| *Spodoptera latifascia* (Walker) | MHMXA734-06 | Costa Rica | CRI | **30** | **29** | **28** | **28** |
| *Spodoptera latifascia* (Walker) | MHMXA735-06 | Costa Rica | CRI | **30** | **29** | **28** | **28** |
| *Spodoptera latifascia* (Walker) | MHAUG697-07 | Costa Rica | CRI | **30** | **29** | **28** | **28** |
| *Spodoptera latifascia* (Walker) | MNHN46** | Mexico | MEX | **30** | **29** | **28** | **28** |
| *Spodoptera latifascia* (Walker) | MNHN47** | Guatemala | GTM | **30** | **29** | **28** | **28** |
| *Spodoptera latifascia* (Walker) | MNHN48** | Costa Rica | CRI | **30** | **29** | **28** | **28** |
| *Spodoptera latifascia* (Walker) | MNHN51** | Dominican Republic | DOM | **30** | **29** | **28** | **28** |
| *Spodoptera latifascia* (Walker) | B123* | USA - Georgia | USA | **30** | **29** | **28** | **28** |
| *Spodoptera latifascia* (Walker) | B18* | Guadeloupe | GLP | **30** | **29** | **28** | **28** |
| *Spodoptera latifascia* (Walker) | B29* | Guadeloupe | GLP | **30** | **29** | **28** | **28** |
| *Spodoptera latifascia* (Walker) | B3* | USA - Louisiana | USA | **30** | **29** | **28** | **28** |
| *Spodoptera latifascia* (Walker) | B31* | Barbados | BRB | **30** | **29** | **28** | **28** |
| *Spodoptera latifascia* (Walker) | B33* | USA - Louisiana | USA | **30** | **29** | **28** | **28** |
| *Spodoptera latifascia* (Walker) | B36* | Barbados | BRB | **30** | **29** | **28** | **28** |
| *Spodoptera latifascia* (Walker) | B39* | USA - Louisiana | USA | **30** | **29** | **28** | **28** |
| *Spodoptera latifascia* (Walker) | LSU17* | USA | USA | **30** | **29** | **28** | **28** |
| *Spodoptera latifascia* (Walker) | LSU6* | Guadeloupe | GLP | **30** | **29** | **28** | **28** |
