# Supplementary material for: Phylogenetic Molecular Species Delimitations Unravel Potential New Species in the Pest Genus Spodoptera Guenée, 1852 (Lepidoptera, Noctuidae)
Source: PLoS One. 2015 Apr 8;10(4):e0122407. doi: 10.1371/journal.pone.0122407 (PMC4390195; doi:10.1371/journal.pone.0122407)
Supplement: S1 Fig — Support values are provided on major nodes (only BV ≥ 50% are shown). (PDF) [file pone.0122407.s001.pdf]

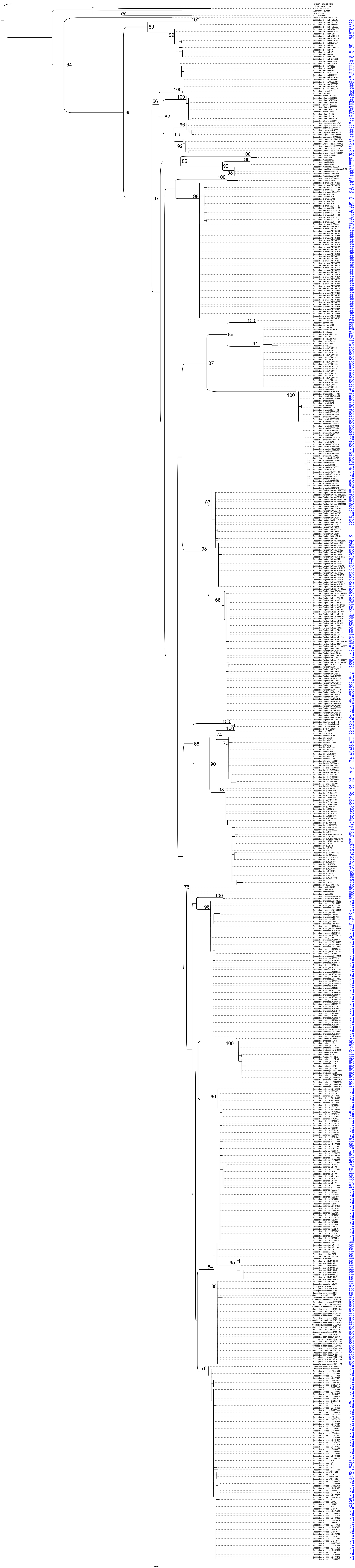

70
100
89
99
64
95
100
56
100
62
96
86
92
86
100
96
99
98
100
98
67
100
86
91
87
100
87
98
68
100
100
74
73
66
90
93
76
100
96
100
96
84
95
88
76
0.02
